# Characterizing the polygenic overlap and shared loci between rheumatoid arthritis and cardiovascular diseases

**DOI:** 10.1186/s12916-024-03376-1

**Published:** 2024-04-08

**Authors:** Xiaohui Sun, Yu Qian, Weiqiu Cheng, Ding Ye, Bin Liu, Dan Zhou, Chengping Wen, Ole A. Andreassen, Yingying Mao

**Affiliations:** 1 Department of Epidemiology, School of Public Health, Zhejiang Chinese Medical University, Hangzhou, 310053 China; 2 School of Life Sciences, Westlake University, Hangzhou, 310024 China; 3 NORMENT, Institute of Clinical Medicine, University of Oslo, Oslo, 0407 Norway; 4 School of Public Health and the Second Affiliated Hospital, Zhejiang University School of Medicine, Hangzhou, China; 5 College of Basic Medical Sciences, Zhejiang Chinese Medical University, Hangzhou, 310053 China

**Keywords:** Rheumatoid arthritis, Cardiovascular diseases, Epidemiological association, Genetic correlation, Pleiotropic loci

## Abstract

**Background:**

Despite substantial research revealing that patients with rheumatoid arthritis (RA) have excessive morbidity and mortality of cardiovascular disease (CVD), the mechanism underlying this association has not been fully known. This study aims to systematically investigate the phenotypic and genetic correlation between RA and CVD.

**Methods:**

Based on UK Biobank, we conducted two cohort studies to evaluate the phenotypic relationships between RA and CVD, including atrial fibrillation (AF), coronary artery disease (CAD), heart failure (HF), and stroke. Next, we used linkage disequilibrium score regression, Local Analysis of [co]Variant Association, and bivariate causal mixture model (MiXeR) methods to examine the genetic correlation and polygenic overlap between RA and CVD, using genome-wide association summary statistics. Furthermore, we explored specific shared genetic loci by conjunctional false discovery rate analysis and association analysis based on subsets.

**Results:**

Compared with the general population, RA patients showed a higher incidence of CVD (hazard ratio [HR] = 1.21, 95% confidence interval [CI]: 1.15–1.28). We observed positive genetic correlations of RA with AF and stroke, and a mixture of negative and positive local genetic correlations underlying the global genetic correlation for CAD and HF, with 13 ~ 33% of shared genetic variants for these trait pairs. We further identified 23 pleiotropic loci associated with RA and at least one CVD, including one novel locus (rs7098414, *TSPAN14*, 10q23.1). Genes mapped to these shared loci were enriched in immune and inflammatory-related pathways, and modifiable risk factors, such as high diastolic blood pressure.

**Conclusions:**

This study revealed the shared genetic architecture of RA and CVD, which may facilitate drug target identification and improved clinical management.

**Supplementary Information:**

The online version contains supplementary material available at 10.1186/s12916-024-03376-1.

## Background

Rheumatoid arthritis (RA) is a systemic autoimmune disease that affects approximately 2% of the global population [1]. Previous studies have characterized RA by excess morbidity and mortality from cardiovascular disease (CVD) [2]. Compared with the general population, patients with RA have a 1.48-fold higher incidence of CVD [3, 4]. Recent research has identified traditional risk factors for CVD comorbidity in RA, such as obesity, hypertension, and dyslipidemia [5]. However, after controlling for these established risk factors, patients with RA still showed an increased risk of developing CVD [6, 7]. Therefore, the pathology underlying the phenotypic linkage between RA and CVD requires further investigation.

As both RA and CVD are complex and heritable disorders [8–10], one hypothesis accounting for their phenotypic linkage is the genetic components [11, 12]. The genetic variants may either influence one trait through their effect on the other (causality) or have independent effects on both traits (pleiotropy). Several studies applying the Mendelian randomization approach, which uses genetic variants as proxies for exposures, showed that genetic liability to RA was associated with an increased risk of coronary artery disease (CAD), intracerebral hemorrhage and myocardial infarction, suggesting the potential causality of RA on CVD risk [13, 14]. Except for the potentially causal effects, the shared genetic variants may also contribute to their associations. Prior studies reported several genes, such as *HLA-DRB1*, *TNFA*, *MTHFR*, and *CCR5*, were associated with the developments of both RA and CVD [15–17]. However, a comprehensive assessment through genome-wide analyses has not been performed. The accumulating amount of genome-wide genetic data enable the utilization of several newly developed methods, such as conjunctional false discovery rate (conjFDR) analysis and association analysis based on subsets (ASSET), to discover novel susceptibility regions affecting multiple traits. Such design may help to examine the genetic contributions to the epidemiological associations and provide novel insights into the underlying biological mechanisms.

**Fig. 1:**
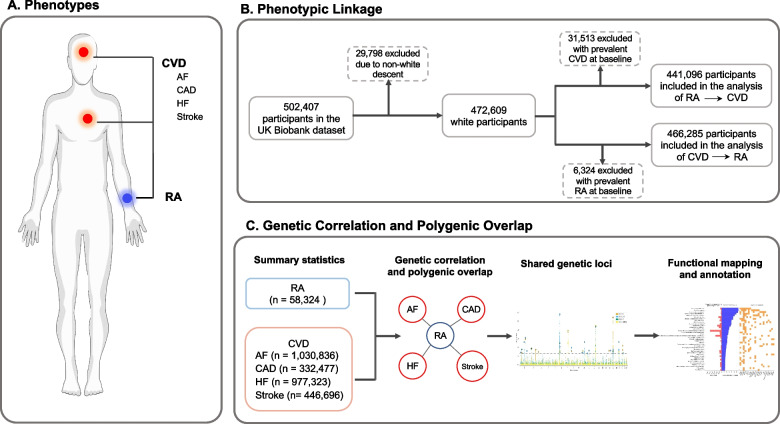
The overall design of present study. **A** Rheumatoid arthritis and four cardiovascular diseases traits, including atrial fibrillation, coronary artery disease, hear failure and stroke, were included in the present study; **B** Phenotypic correlations between rheumatoid arthritis and cardiovascular diseases were assessed in UK Biobank dataset; **C** Genetic correlation and polygenic overlap between rheumatoid arthritis and cardiovascular diseases were estimated using summary statistics. Abbreviations: AF, atrial fibrillation; CAD, coronary artery disease; HF, heart failure; RA, rheumatoid arthritis

To quantify the shared and distinct etiology underlying RA and CVD, we comprehensively analyzed phenotypic linkage and polygenic overlap between RA and four CVD traits, including AF, CAD, HF, and stroke. The overall design of the study is shown in Fig. 1. Specifically, we first assessed the phenotypic linkage between RA and four CVD traits using longitudinal data from the UK Biobank [18–22]. Next, by integrating the large-scale genome-wide association studies (GWAS), we evaluated the genetic correlation and identified shared genetic loci between RA and CVD. We finally carried out functional annotations to delineate the biological impact of shared loci. By investigating the phenotypical and genetic associations between RA and CVD, along with the underlying biological mechanisms, the present study may provide insights into the clinical management of these diseases.

## Methods

### Data sources

#### The UK Biobank sataset and GWAS summary statistics

We included European participants with phenotypic data from the UK Biobank (Project ID:71,610). The diagnoses of RA, AF, CAD, HF, and stroke were defined using the International Classification of Diseases code (both Ninth Revision [ICD-9] and Tenth Revision [ICD-10]) and self-reported illness (Additional file 1: Table S1).

To assess the phenotypic linkage between RA and CVD, we performed two analyses (the RA-CVD cohort and the CVD-RA cohort). Specifically, when assessing the risk of CVD in RA patients, we excluded participants with prevalent CVD at baseline, resulting in a total of 441,096 participants (namely the RA-CVD cohort). To analyze RA risk in CVD patients, we excluded participants diagnosed with RA at baseline, the analytic sample comprised 466,285 participants ( the CVD-RA cohort). The UK Biobank study was approved by the North West Multi-Center Research Ethics Committee, and all participants provided informed consent. More details are displayed in the Methods in the Additional file 1 [23–25].

Summary statistics of RA and four CVD traits were obtained from publicly available GWAS, ranging from 58,324 to 1,030,836 individuals of European ancestry [18–22]. Detailed information is provided in Additional file 1: Table S2 and the Methods in the Additional file 1 [18–22, 26].

### Statistical analyses

#### Observational analyses

To estimate the phenotypic linkage between RA and CVD, we calculated the hazard ratios (HR) with 95% confidence intervals (CI), using the multivariable Cox proportional hazards regression model. The HRs were progressively adjusted for confounders in two models. Model 1 included age and sex as covariates. Model 2 was adjusted for age, sex, body mass index [BMI], smoking status, alcohol drinking frequency, educational attainment, physical activities, hypertension, type 2 diabetes, and total cholesterol, when assessing the associations between RA and CVD risk. When evaluating the relationship between CVD and RA risk, Model 2 only contained age, sex, BMI, smoking status, alcohol drinking frequency, educational attainment and physical activities. We additionally performed propensity score analyses, and a series of sensitivity analyses, including stratifying participants by age and sex, excluding the incident cases in the first follow-up year, and excluding participants with kinship.

### Analyses of genetic correlation and polygenic overlap

We calculated the global and local genetic correlation as well as the polygenic overlap between RA and CVD. We first applied linkage disequilibrium score regression (LDSC) to estimate the global genetic correlation using the 1000 Genomes Project European panel as the reference panel [27]. To investigate whether RA shares genetic overlap with CVD in specific genomic regions, we supplied the Local Analysis of [co]Variant Association (LAVA) method to estimate the local genetic correlation [28]. A bivariate test was performed to estimate these local genetic correlations based on the local genetic covariance between two traits [28]. A total of 2495 default linkage disequilibrium (LD)—independent genomic regions were applied in LAVA [28]. Additionally, we used the bivariate causal mixture model (MiXeR) to quantify the polygenic overlap beyond genetic correlations [29]. MiXeR estimated the number of trait-influencing variants (i.e., variants with non-zero additive genetic effects on the trait) for each trait and the number of shared trait-influencing single-nucleotide polymorphisms (SNPs) based on a bivariate Gaussian mixture model [29]. Model fit was evaluated by the Akaike Information Criterion (AIC) based on the likelihood maximization of signed test statistics (GWAS *z*-scores). A positive AIC value suggested sufficient power of input GWAS data to distinguish the estimated polygenic overlap. More details for the framework of MiXeR are provided in its original publication [29].

### Discovery of shared genetic loci

To discover the shared genetic loci, we performed conjunctional false discovery rate (conjFDR) analysis on corresponding GWAS summary statistics. As an extension of the conditional FDR (condFDR) method, conjFDR is an empirical Bayesian statistical framework and re-ranks the test statistics in a primary phenotype (eg. RA) by conditioning on SNP associations with a secondary phenotype (e.g., CVD) [30, 31]. After switching the roles of the primary and secondary traits, the conjFDR is determined as the maximum of two condFDR values, which provides a conservative estimate of the FDR for the association with both phenotypes [30, 32]. In the present study, we applied a conjFDR threshold of 0.05.

As an alternative approach, we applied the association analysis based on subsets (ASSET) for genome-wide cross-trait meta-analysis to validate the results from conjFDR [33]. We used two-sided ASSET analysis, which allows for subset searches for association signals separately in positive and negative directions and then combines association signals from two directions by chi-square test [33]. The genome-wide significant variants were determined as *P* < 5 × 10^−8^.

To define independent genetic loci, we clumped the results of conjFDR and ASSET (–clump-kb 500; –clump-r2 0.1) using PLINK, separately [34]. For each shared locus found by conjFDR, we examined whether it was physically overlapped with a locus from ASSET, based on their lead SNPs using 500 kb windows. If a conjFDR locus overlapped with a locus from ASSET, we considered it as validated in ASSET, and such locus remained for subsequent analyses. We then assessed if a genetic locus was novel for RA and CVD based on a 500-kb region upstream and downstream of its lead SNP. We regarded a locus to be novel if it had not been previously reported to be associated with RA or CVD at genome-wide significance, according to the GWAS Catalog (latest accessed date on July 10th, 2021) and previously published GWAS [18–22].

#### Functional annotations and gene prioritization

We annotated the regulatory function for the lead SNPs, including enhancers and histone modification sites [35, 36]. The lead SNPs were further mapped to genes by a combination of physical position, expression quantitative trait loci (eQTL), and chromatin interaction mappings. These mapped genes were referred to as candidate-shared genes. Detailed information is displayed in the Methods in the Additional file 1 [35–40].

We prioritized these candidate shared genes by summary-data-based Mendelian randomization (SMR) analyses, which investigate the associations between the expression of shared genes and phenotypes of interest [41]. Cis-eQTLs in eQTLGen Consortium [42] and five tissues (artery aorta, artery coronary, atrial appendage, left ventricle, and whole-blood tissues) from GTEx v8 projects were used as instrumental variables to link the outcome via an exposure (i.e., gene expression) [40]. The analysis was conducted according to default SMR protocols. We further supplied the heterogeneity in dependent instruments (HEIDI) analyses and colocalization analyses to support inference by reducing the likelihood that linkage disequilibrium affected the MR findings.

### Gene functional enrichment analysis

We explored the underlying biological mechanisms for those shared genes by evaluating their enrichment in gene ontology (GO) and Kyoto Encyclopedia of Genes and Genomes (KEGG) pathways using KOBAS 3.0 [43]. Meanwhile, we examined the enrichment of shared genes in any other traits based on the dataset from the GWAS Catalog, using the Gene2Func function of FUMA [44]. The significance threshold of all the above enrichment analyses was set at FDR < 0.05.

## Results

### The phenotypic linkage between RA and CVD

**Fig. 2:**
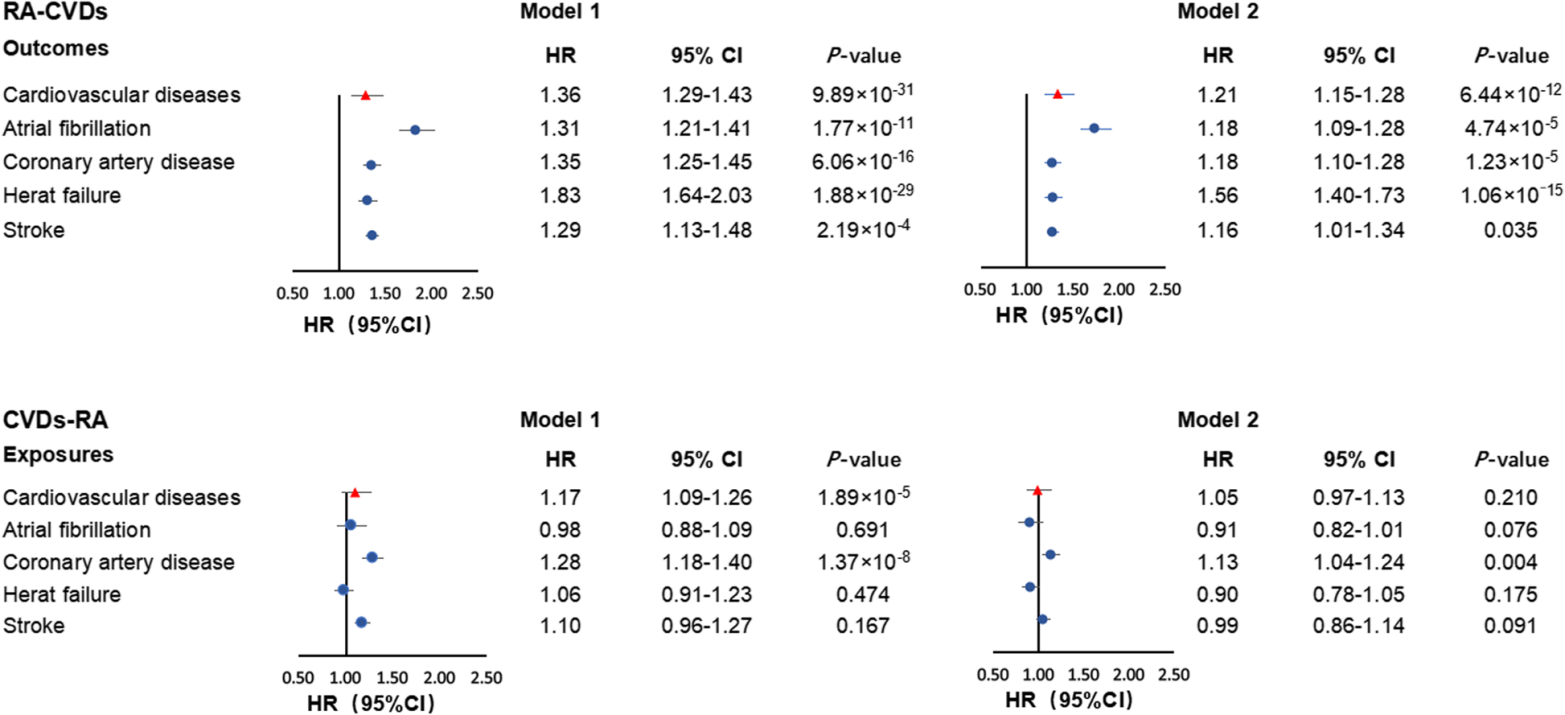
Forest plot of multivariable cox-regression estimates for the bidirectional associations between rheumatoid arthritis and cardiovascular diseases. **A** The associations between rheumatoid arthritis and incident cardiovascular diseases in the UK Biobank; **B** The association between cardiovascular diseases and incident rheumatoid arthritis in the UK Biobank. *Cardiovascular diseases included atrial fibrillation, coronary artery disease, heart failure, and stroke. Abbreviations: CI, confidence intervals; HR, hazard ratio

The characteristics of the participants are displayed in Additional file 1: Table S3. We first assessed the association between RA and the incidence of CVD in the UK Biobank. We observed an increased incidence of CVD in patients with RA in both Model 1 (HR = 1.36, 95% CI: 1.29–1.43) and Model 2 (HR = 1.21, 95% CI: 1.15–1.28). In the fully adjusted model (Model 2), compared with non-RA controls, patients with RA had a 18% (95% CI: 9–28%) higher risk for AF, 18% (95% CI: 10–28%) higher risk for CAD, 56% (95% CI: 40–73%) higher risk for HF, and 16% (95% CI: 1–34%) higher risk for stroke, respectively (Fig. 2). The increased risk of CVD in patients with RA remained statistically significant in additional analyses, including stratification analyses by age and sex, sensitivity analyses excluding incident cases diagnosed in the first follow-up year, and excluding participants with kinship, as well as in propensity score analyses (Additional file 1: Table S4). The results for the associations between RA and four CVDs in the additional analyses can be found in the Additional file 1: Table S4.

Meanwhile, we investigated the relationship between CVD and the incidence of RA (Fig. 2 and Additional file 1: Table S5). After adjustment for age and sex, patients with CVD had a higher risk of RA (HR = 1.17, 95% CI: 1.09–1.26) (Model 1). Among them, patients with CAD had an elevated risk of RA (HR = 1.28, 95% CI: 1.18–1.40). However, in the fully adjusted Cox regression models (Model 2), only patients with CAD had a higher incidence of RA, with HR of 1.13 (95% CI: 1.04–1.24). Sensitivity analyses yielded consistent results (Additional file 1: Table S6). However, we observed a statistically significant association between CVD and RA risk among the younger participants (< 60 years old) (HR: 1.58, 95% CI: 1.38–1.80; *P* = 1.22 × 10^−11^) (Additional file 1: Table S6).

### The genetic correlation and polygenic overlap between RA and CVD

**Fig. 3:**
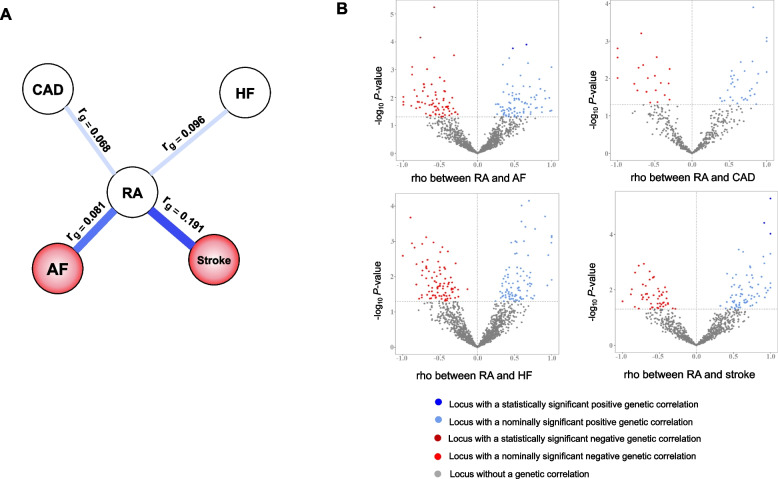
The genetic correlation and polygenic overlap between rheumatoid arthritis and cardiovascular diseases. **A** The genetic correlation between rheumatoid arthritis and cardiovascular diseases using LDSC. The circles filled with colors suggest significant global genetic correlations between the diseases; **B** The volcanic plots of LAVA analyses. Abbreviations: AF, atrial fibrillation; CAD, coronary artery disease; HF, heart failure; RA, rheumatoid arthritis

LDSC analyses revealed that RA has significant positive global genetic correlations with AF (*r*_*g*_ = 0.081, FDR-adjusted *P* = 0.025) and stroke (*r*_*g*_ = 0.191, FDR-adjusted *P* = 0.008). Nominally significant positive global genetic correlations were found for RA with CAD (*r*_*g*_ = 0.068, FDR-adjusted *P* = 0.080) and HF (*r*_*g*_ = 0.096, FDR-adjusted *P* = 0.087) (Fig. 3A). In MiXeR analyses, conditional QQ plots showed cross-trait SNP enrichment for RA and CVD (Additional file 1: Fig S1), suggesting there were polygenic overlaps. Among the 0.8 k variants associated with RA, about 13 ~ 33% of them were also associated with CVD, including 0.1 k shared variants with AF, and 0.3 k with CAD (Additional file 1: Table S8). There were 45% and 46% shared genetic variants between RA and HF, RA and stroke, respectively; however, the model fit suggested the need to increase the sample size of GWAS to provide sufficient power (Fig. S1, and Table S2 in the Additional file 1).

As global genetic correlation estimates an average of genome-wide shared association, these nominal significant correlations may be led by the mixed shared associations [28, 29, 45]. Thus, we further applied LAVA to detect local genetic correlation. The LAVA analysis showed a mixture of negative and positive local genetic correlations underlying the global positive genetic correlation. Specifically, we observed that around 51% to 61% of nominally significant local genetic correlations were positive (Fig. 3B and Additional file 1: Table S7). Moreover, 7 regions, including 4 for RA and AF and 3 for RA and stroke, were significantly correlated. Taken together, the above findings suggested a shared genetic architecture between RA and CVD.

### Shared genetic loci between RA and CVD

**Fig. 4:**
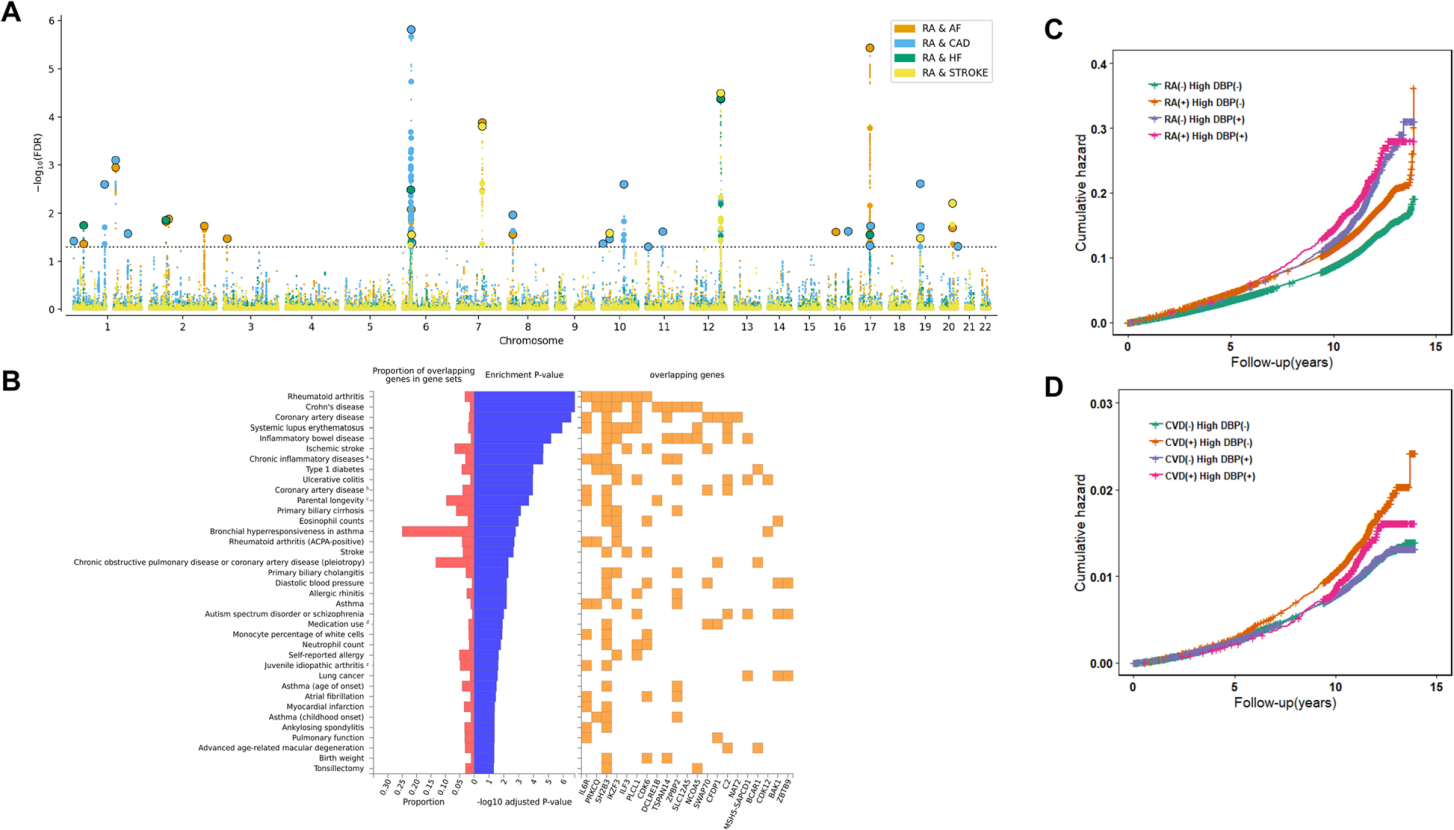
The Manhattan plot of conjFDR results and shared genetic loci enrichment. **A** The Manhattan plot of conjFDR results. The *x*-axis is the position of genetic variant in the genome, and *y*-axis is the -log_10_ (*P*-value) of the corresponding genetic variant; **B** The enriched traits in the GWAS catalog. ^a^ includes the following: ankylosing spondylitis, Crohn’s disease, psoriasis, primary sclerosing cholangitis, ulcerative colitis; ^b^ includes the following: myocardial infarction, percutaneous transluminal coronary angioplasty, coronary artery bypass grafting, angina or chronic ischemic heart disease; ^c^ includes the following: combined parental attained age, Martingale residuals; ^d^ includes the following: agents acting on the renin-angiotensin system; ^e^ includes the following: oligoarticular or rheumatoid factor-negative polyarticular; **C** The cumulative hazard of cardiovascular diseases stratified by rheumatoid arthritis and diastolic blood pressure. Participants were grouped on the basis of their diabolic blood pressure levels (normal [< 90 mmHg] vs. high [≥ 90 mmHg]) and rheumatoid arthritis (no vs. yes): RA( −) DBP( −), RA( +) DBP( −), RA( −) DBP( +) and RA( +) DBP( +). **D** The cumulative hazards of rheumatoid arthritis stratified by cardiovascular diseases and diastolic blood pressure. Participants were grouped on the basis of their diabolic blood pressure levels (normal [< 90 mmHg] vs. high [≥ 90 mmHg]) and cardiovascular disease (no vs. yes): CVD( −) DBP( −), CVD( +) DBP( −), CVD( −) DBP( +) and CVD( +) DBP( +)

In light of the above findings, we performed further analyses to detect the loci shared between RA and CVD. First, by using conjFDR analysis, we identified 12, 19, 5, and 6 loci shared between RA and AF, CAD, HF, and stroke, respectively (Fig. 4, and Additional file 1: Table S9). Next, we assessed these identified shared loci using ASSET analysis (Additional file 1: Table S10), and validated 23 loci, including 10, 13, 5, and 5 shared between RA and AF, CAD, HF, and stroke, respectively (Table 1). A total of 27 candidate-shared genes were mapped by these identified loci (Table 1).

**Table 1:** The detailed information of shared SNPs between rheumatoid arthritis and cardiovascular diseases

Locus	Lead SNP	CVD traits	CHR	Position	Effect allele	Other allele	Annotation	Candidate gene
1	rs2843151	CAD	1	2,245,633	C	T	Intergenic	NA
2	rs12022363	HF	1	38,252,609	G	A	Intergenic	NA
3	rs11552449	CAD	1	114,448,389	T	C	Exonic	DCLRE1B (e)
4	rs12126142	AF; CAD	1	154,425,456	A	G	Intronic	IL6R (c,e)
5	rs62149420	AF; HF	2	61,094,810	G	A	ncRNA_intronic	LINC01185
6	rs4563251	AF	2	70,251,490	C	T	ncRNA_intronic	PCBP1-AS1 (e)
7	rs700677	AF	2	198,702,424	A	C	Intronic	PLCL1 (e)
8	rs2454429	AF	3	12,620,720	G	A	Intronic	MKRN2 (e)
9	rs2905734	HF	6	31,453,711	C	A	ncRNA_intronic	MICB-DT
9	rs409558	AF	6	31,708,147	C	T	ncRNA_intronic	MSH5-SAPCD1
9	rs3130683	CAD	6	31,888,367	C	T	Intronic	C2
10	rs2772373	Stroke	6	33,429,672	G	A	Intergenic	ZBTB9 (e), BAK1 (e)
11	rs42034	AF	7	92,239,144	G	A	3′UTR	CDK6 (c)
11	rs42039	Stroke	7	92,244,422	T	C	3′UTR	CDK6 (c)
12	rs13277738	AF	8	18,241,507	T	C	Intergenic	NAT2 (e)
13	rs4750517	CAD	10	6,520,458	G	A	Intronic	PRKCQ (e)
14	rs4749532	CAD	10	30,418,323	T	C	Intergenic	NA
15	rs7098414	CAD	10	82,214,586	A	C	Intronic	TSPAN14 (e)
16	rs10840298	CAD	11	9,764,832	C	A	Intronic	SWAP70 (c,e)
17	rs10774624	Stroke; CAD; HF	12	111,833,788	G	A	Intergenic	PHETA1 (e), SH2B3 (e)
18	rs12919951	CAD	16	75,306,890	G	A	Intergenic	BCAR1 (e), CFDP1(e)
19	rs8075737	CAD	17	37,727,316	T	C	Intergenic	CDK12 (c,e)
19	rs9747973	AF	17	37,905,107	T	C	Intergenic	IKZF3 (e)
19	rs11658278	HF	17	38,031,164	T	C	Intronic	ZPBP2 (e)
20	rs7248558	Stroke; CAD	19	10,770,292	A	G	Intronic	ILF3 (e)
21	rs2421206	CAD	19	11,262,477	G	T	Intronic	SPC24 (e)
22	rs2297199	AF	20	44,674,743	C	A	Intronic	SLC12A5
23	rs6074012	Stroke	20	44,700,166	T	C	Intronic	NCOA5

The candidate genes with Hi-C evidence were marked by “c”, while those with eQTL evidence were marked by “e”

Abbreviations: *AF*, atrial fibrillation; *CAD*, coronary artery disease; *CVD*, cardiovascular diseases; *HF*, heart failure; *RA*, rheumatoid arthritis

Among the replicated loci, one shared locus (rs7098414, 10q23.1, conjFDR = 0.003; *P*_*ASSET*_ = 3.08 × 10^−9^) for RA and CAD was novel for both traits. The lead SNP rs7098414 is located in the intron of *TSPAN14* and overlapped with 13 histone modification markers (Additional file 1: Table S11). Moreover, eQTL analyses showed that the SNP rs7098414 was associated with the expression of *TSPAN14* (*P* = 1.83 × 10^−41^) in whole blood. Notably, we also found two loci, rs10774624, and rs12126142, shared by multiple traits. rs10774624 (12q24.12), was shared between RA and CAD (conjFDR = 4.24 × 10^−5^; *P*_*ASSET*_ = 5.82 × 10^−15^), stroke (conjFDR = 3.20 × 10^−5^; *P*_*ASSET*_ = 4.58 × 10^−17^) and HF (conjFDR = 4.12 × 10^−5^; *P*_*ASSET*_ = 1.55 × 10^−15^). Within this locus, we observed five histone modification markers (Additional file 1: Table S11). The lead SNP rs10774624 resides between *PHETA1* and *SH2B3* and was associated with expressions of both genes in whole blood.

Moreover, SMR analysis suggested that the expression of *SWAP70* was associated with the risk of RA (OR = 0.79, 95% CI = 0.68–0.91, *P* = 0.001) and two CVD traits (CAD: OR = 0.85, 95% CI = 0.80–0.91, *P* = 1.97 × 10^−7^; and HF: OR = 0.91, 95% CI = 0.86–0.96, *P* = 6.24 × 10^−4^), with no evidence of heterogeneity (*P* > 0.05, Additional file 1: Table S12). eQTL for *SWAP70* in whole blood tissue from eQTLGen Consortium were also colocalized with the GWAS of RA, CAD, and HF at this locus (PPH4: 0.87 for eQTL-CAD trait pair; PPH4: 0.56 for eQTL-RA trait pair; PPH4: 0.52 for eQTL-HF trait pair, Additional file 1: Table S12).

### Enriched biological pathways and shared risk factors between RA and CVD

To determine the over-represented pathways among 27 candidate-shared genes, we carried out GO and KEGG pathway enrichment analyses. As a result, we found that shared genes significantly enriched the regulation of the cell cycle and cyclin binding in the biological process. As to cellular components, they were enriched in the actin cytoskeleton, and cyclin-dependent protein serine/threonine kinase activity. For molecular function, these genes were significantly enriched in the protein homodimerization activity, and protein binding, as well as in the regulation of insulin receptor signaling pathway (Additional file 1: Table S13). There were 21 biological pathways significantly enriched, including the human cytomegalovirus infection, and GABAergic synapse (Additional file 1: Table S14).

Additionally, based on the GWAS catalog, these shared genes were mainly enriched in chronic inflammatory diseases, such as Crohn’s disease, systemic lupus erythematosus, and Type 1 diabetes (Fig. 4B). Besides, the shared genes were also enriched in diastolic blood pressure, one risk factor for CVD (Fig. 4B). Hence, we applied a factorial analysis to evaluate the associations of diastolic blood pressure with the risk of RA and CVD in the UK Biobank. As shown in Fig. 4C, participants with neither RA nor high diastolic blood pressure had the lowest risk of CVD (any of AF, CAD, HF, and stroke). We observed an incremental risk in patients with RA alone (HR = 1.05, 95% CI: 1.02–1.07), followed by patients with high diastolic blood pressure alone (HR = 1.20, 95% CI: 1.13–1.28), and the risk was the highest in patients with both RA and high diastolic blood pressure (HR = 1.22, 95% CI: 1.09–1.36) (Additional file 1: Table S15). However, the combination of high diastolic blood pressure and CVD was not significantly positively associated with a higher incidence of RA with adjustment of age, sex, BMI, smoking status, alcohol drinking frequency, education levels and physical activity (Additional file 1: Table S15).

## Discussion

In the present study, we systematically investigated the phenotypic linkage and the shared genetic architecture between RA and CVD. We identified 23 shared loci, including one novel locus for RA and CVD. Genes mapped by these shared loci suggested the involvement of immune and inflammatory-related pathways as well as modifiable risk factors such as high diastolic blood pressure for their etiology.

Using data including more than 440,000 UK Biobank participants, our results revealed a higher incidence of CVD in RA patients, with HRs ranging from 1.16 to 1.56, which were consistent with previous epidemiological evidence [46, 47]. We noted that the association between RA and CAD exhibited significance among females and younger participants, whereas it diminished to insignificance among males and older individuals. Reasons for the insignificant association in the sensitivity analyses may be diverse. It is possible that the association between RA and CVD may differ by age, sex, or could be influenced by the limited sample size [48]. In this study, we also evaluated the relationship between CVD and the incidence of RA, revealing a significant association solely between CAD and RA. Further investigation may be warranted to validate our findings.

The epidemiological associations between RA and CVD may be the result of pleiotropic effects of genetics components, reflected by the global and local genetic correlations. In the present study, we observed significant positive global genetic correlations of RA with AF and stroke. However, the global genetic correlations of RA with CAD and HF were of nominal significance. Since the global genetic correlation represents the average of genome-wide shared associations, this insignificant global correlation may be due to opposing directions at different regions [45]. Consistently, LAVA analysis revealed a mixed effect direction at the regional level, by showing nearly half of the regions were positively correlated with nominal significance. The shared genetic architecture hypothesis was further supported by MiXeR, which found that RA shares 33.3% genetic variants with CAD and 44.4% with HF and reported cross-trait SNP enrichment in conditional Q-Q plots. Altogether, the above findings supported the presence of polygenic overlap between RA and CVD.

Among the shared loci, one locus on chromosome 10 has not been linked to RA or CVD in previous GWAS studies. The lead SNP of this locus is rs7098414 (10q23.1), an intronic SNP of *TSPAN14. TSPAN14* encodes Tspan14 protein, which belongs to a subfamily of six related proteins TspanC8 group [49, 50]. *TSPAN14* has been found to positively regulate ADAM10-dependent Notch activation [51], which was involved in the pathogenesis of RA [52]. *TSPAN14* was also shown to interact with the inflammatory pathway of atherosclerosis, which has a major role in the development of CAD [53]. Further research is needed to better understand the specific mechanisms through which Tspan14 influences these diseases and to validate the functional significance of *TSPAN14* in RA and CAD. Besides, the other shared loci also provided new insights into the pathogenesis of the association between RA and CVD. For example, rs10774624 (12q24.12), which was jointly associated with RA, CAD, HF, and stroke in the present study, has been demonstrated to be related to blood pressure and peripheral artery disease [54, 55]. Moreover, the identification of rs12126142 (*IL6R*) shared with RA, AF, and CAD may be of specific clinical interest. The *IL6R* gene product has been linked to immunological response [56] and chronic inflammation [57], both of which have been implicated in the pathogenesis of RA and CVD. Consistently, prior studies also reported that genetically determined lower IL-6 level was associated with a decreased risk of coronary heart disease events [58, 59]. The pathway enrichment analysis also suggested the involvement of Th17 cell differentiation, which is regulated by IL-6 [60]. Currently, several drugs, such as Tocilizumab, have been developed to inhibit IL6 signaling for RA treatment [61]. Thus, our findings of shared genetic loci between RA and CVD may shed light on the clinical implications of individualized treatment to reduce the occurrence of CVD in RA patients.

Additionally, GO enrichment analysis suggested a potential involvement of negative regulation of insulin receptor signaling pathways in the comorbidity of RA and CVD. This signaling pathway may be of special clinical interest, given the established role of insulin resistance in the development of CVD [62] and its suggested involvement in the risk of RA in patients with CVD [63]. Moreover, the shared genetic loci were also enriched in diabolic blood pressure, which can be caused by insulin resistance [64, 65]. These data suggested that the metabolic mechanisms may be relevant to the association of RA and CVD.

Some limitations should be considered when interpreting our findings. First, when assessing the associations between RA and CVD in the UK Biobank, though we have included the potential confounding factors in the our analysis, residual confounding may still exist, which may result in an overestimation of the association. Second, the MiXeR analyses for RA-HF, and RA-stroke still suggested inadequate power, though we applied the GWAS data sets with the largest sample size to date for each phenotype assessed. Further validation studies of the present findings using larger GWAS datasets are required. Additionally, due to available GWAS data and multiancestral differences in allele frequency and LD structure, we restricted our analyses to the European population, therefore, these findings may not be generalizable to other populations with different ethnicities. Future investigations should incorporate more diverse population samples, such as non-European cohorts, to validate and extend our results. Finally, several included GWAS datasets did not include the major histocompatibility complex (MHC) region, which precluded us from exploring genetic linkage for RA and CVD in the MHC region. Since the MHC region has a well-established role in immune responses and inflammation, and the present study indicated the involvement of inflammation in the shared genetic loci between RA and CVD, further studies with a focus on MHC are suggested.

## Conclusions

The present study showed phenotypic linkages between RA and CVD, as well as shared genetic components, which might underlie the established epidemiological associations. The shared genes were involved in immune and inflammatory pathways. These findings progress our understanding of shared genetic mechanisms underlying RA and CVD and may provide insights into new therapies for these disorders and improve clinical management.

### Supplementary Information

**Additional file 1: Supplementary Fig. 1.** Polygenic overlap between rheumatoid arthritis and cardiovascular diseases: (A) RA and AF; (B) RA and stroke; (C) RA and HF; and (D) RA and CAD. Each sub-figure contained Venn Diagrams, conditional Q-Q plots, and negative log-likelihood plot, respectively. Venn diagrams showed the polygenic overlap (gray) between RA (blue) and the corresponding CVD phenotype (orange). The numbers in the Venn diagram indicate the estimated number of variants (in thousands) explained 90% of heritability in the corresponding phenotype, followed by the standard error. Conditional Q–Q plots of observed versus expected -log10 *P*-values in the primary trait as a function of significance of association with a secondary trait at the different threshold of *P*-values. The Black dotted line indicate the null hypothesis. In the negative log-likelihood plot, minus log-likelihood is calculated for the bivariate model as a function of parameter. Abbreviations: AF, atrial fibrillation; CAD, coronary artery disease; HF, heart failure; RA, rheumatoid arthritis. **Table S1.** The ICD-9 diagnosis codes, ICD-10 diagnosis codes, and self-reported codes in the UK Biobank for rheumatoid arthritis and cardiovascular diseases. **Table S2.** Detailed information of summary statistics of rheumatoid arthritis and cardiovascular diseases. **Table S3.** Basic characteristics of individuals included in the RA-CVD cohort. **Table S4.** Propensity score and stratified analyses of associations between rheumatoid arthritis and cardiovascular diseases risk. **Table S5.** Basic characteristics of individuals included in the CVD-RA cohort. **Table S6.** Propensity score and stratified analyses of associations between cardiovascular diseases and rheumatoid arthritis risk. **Table S7.** The regions with nominally significant genetic correlation were identified from LAVA analysis. **Table S8.** The results of the model fit in MeXiR analysis. **Table S9.** The independent pleiotropic loci based on conjFDR analyses. **Table S10.** The independent pleiotropic loci based on ASSET analyses. **Table S11.** The results of histone modification and enhancer enrichment for lead SNPs. **Table S12.** The results of summary-based Mendelian randomization analyses and colocalization results. **Table S13.** The enriched GO terms of the mapped genes. **Table S14.** The enriched KEGG pathway terms of the mapped genes. **Table S15.** The effects of different combinations of rheumatoid arthritis/cardiovascular disease status and overlapped modifiable risk factors (diastolic blood pressure).

## Data Availability

The data generated or analyzed during this study are available in this published article and its supplementary information files.

## Abbreviations

AIC: Akaike Information Criterion
BMI: Body mass index
CAD: Coronary artery disease
CI: Confidence intervals
CondFDR: Conditional false discovery rate
ConjFDR: Conjunctional false discovery rate
CVD: Cardiovascular disease
eQTL: Expression quantitative trait loci
GO: Gene ontology
GWAS: Genome-wide association studies
HF: Heart failure
HR: Hazard ratio
ICD: International Classification of Diseases
KEGG: Kyoto Encyclopedia of Genes and Genomes
LAVA: Local Analysis of [co]Variant Association
LD: Linkage disequilibrium
LDSC: Linkage disequilibrium score regression
RA: Rheumatoid arthritis
SMR: Summary-data-based Mendelian randomization
SNPs: Single-nucleotide polymorphisms
