# Supplementary material for: Characterizing the polygenic overlap and shared loci between rheumatoid arthritis and cardiovascular diseases
Source: BMC Med. 2024 Apr 8;22:152. doi: 10.1186/s12916-024-03376-1 (PMC11003061; doi:10.1186/s12916-024-03376-1)
Supplement: Supplementary file 1 — Additional file 1: Supplementary Fig. 1. Polygenic overlap between rheumatoid arthritis and cardiovascular diseases: (A) RA and AF; (B) RA and stroke; (C) RA and HF; and (D) RA and CAD. Each sub-figure contained Venn Diagrams, conditional Q-Q plots, and negative log-likelihood plot, respectively. Venn diagrams showed the polygenic overlap (gray) between RA (blue) and the corresponding CVD phenotype (orange). The numbers in the Venn diagram indicate the estimated number of variants (in thousands) explained 90% of heritability in the corresponding phenotype, followed by the standard error. Conditional Q–Q plots of observed versus expected -log10 P-values in the primary trait as a function of significance of association with a secondary trait at the different threshold of P-values. The Black dotted line indicate the null hypothesis. In the negative log-likelihood plot, minus log-likelihood is calculated for the bivariate model as a function of parameter. Abbreviations: AF, atrial fibrillation; CAD, coronary artery disease; HF, heart failure; RA, rheumatoid arthritis. Table S1. The ICD-9 diagnosis codes, ICD-10 diagnosis codes, and self-reported codes in the UK Biobank for rheumatoid arthritis and cardiovascular diseases. Table S2. Detailed information of summary statistics of rheumatoid arthritis and cardiovascular diseases. Table S3. Basic characteristics of individuals included in the RA-CVD cohort. Table S4. Propensity score and stratified analyses of associations between rheumatoid arthritis and cardiovascular diseases risk. Table S5. Basic characteristics of individuals included in the CVD-RA cohort. Table S6. Propensity score and stratified analyses of associations between cardiovascular diseases and rheumatoid arthritis risk. Table S7. The regions with nominally significant genetic correlation were identified from LAVA analysis. Table S8. The results of the model fit in MeXiR analysis. Table S9. The independent pleiotropic loci based on conjFDR analyses. Table S [file 12916_2024_3376_MOESM1_ESM.docx]

**Additional file 1**

**Supplementary notes**

1. **Data source**

**1.1 *UK Biobank dataset***

UK Biobank is a large population-based prospective study of 502,407 participants aged 40 to 70 years [23]. The participants were recruited from the general population at 22 assessment centers throughout the United Kingdom. Information on lifestyle and other health-related aspects was collected through extensive baseline questionnaires, interviews, and physical measurements. The RA and CVD diagnoses were defined using the International Classification of Diseases code (both Ninth Revision [ICD-9] and Tenth Revision [ICD-10]) and self-reported illness (**Additional file 1 Table S1**). Age, sex, smoking status, alcohol drinking frequency, education level, body mass index ([BMI], kg/m^2^), physical activity, hypertension, diabetes, and total cholesterol were considered as potential confounders and relevant information was obtained from the initial UK Biobank assessment center visit. Here, we classified education levels as high (individuals who obtained a college or university degree and professional qualifications), middle (individuals who obtained A levels/AS levels, O levels/GCSEs, CSEs, NVQ, or HND. For physical activity, it was captured from the baseline questionnaire of frequency and duration of three levels of activity (walking, moderate and vigorous) [24-25]. We used metabolic equivalents (METs) in hours per week to quantify self-reported physical activity.

**1.2 *GWAS summary statistics datasets***

***Rheumatoid arthritis***

We used the most recent genome-wide association study (GWAS) of rheumatoid arthritis (RA), which included 58,324 participants of European descent (14,361 cases and 43,963 controls) [18]. RA patients were diagnosed by a professional rheumatologist or met the 1987 diagnostic criteria of the American College of Rheumatology [26]. The genetic associations controlled for population stratification and relatedness via adjustment for principal components.

***Atrial fibrillation***

Summary statistics of atrial fibrillation (AF) were extracted from the GWAS meta-analysis published by Nielsen and colleagues [19]. In this study, 60,620 cases and 970,216 controls of European descent from six resources (The Nord-TrøndelagHealth Study, deCODE, the Michigan Genomics Initiative, DiscovEHR, United Kingdom Biobank, and the AFGen Consortium) were involved. The diagnosis of AF was the cohort-specific definition, including electronic health records, diagnosis codes for AF present in the hospital, out-patient, and emergency room discharges as previously described.

***Coronary artery disease***

Genetic variants associated with coronary artery disease (CAD) were obtained from a GWAS of 76,014 cases and 264,785 controls published by Nelson *et al* [20]. Case subjects with CAD were determined with a broad definition including myocardial infarction (MI), percutaneous transluminal coronary angioplasty (PTCA) or coronary artery bypass grafting (CABG), chronic ischemic heart disease, and angina [20].

***Heart failure***

The genetic dataset of heart failure (HF) was based on the largest and newest published GWAS meta-analysis that included 47,309 patients and 930,014 controls of European descent [21]. Detailed information on the definition of HF has been reported in the previous study [21]. In brief, the HF patients were determined by self-reported, diagnosis codes in hospital discharge records and death certificates. In GWAS of HF, the analyses of the underlying studies were adjusted for age, sex, and principal components of ancestry.

***Stroke***

Summary-level data for stroke were extracted from the MEGASTROKE consortium. Briefly, a total of 446,696 European individuals (40,585 cases and 40,611 controls) were included in a meta-analysis of 29 GWASs [22]. Case subjects with stroke were diagnosed as rapidly developing signs of neurological deficit, lasting >24 hours with a vascular origin.

**2. Propensity score analyses**

To minimize the effect of bias, propensity score analyses were performed. Propensity scores were calculated for each participant based on a multivariable logistic regression model. The participants were matched based on age, sex and Townsend deprivation index with a ratio 1:4. We performed propensity score matching using the MatchIt package with nearest-neighbor matching [37].

**3. Functional annotation and Gene Mapping**

We obtained the enhancer dataset from Human Enhancer Disease Database [35], and the histone modifications datasets, which were derived by ChIP-seq in human lymphoblastic cells (GM12878) and human umbilical vein endothelial cells (Huvec), were obtained from the Encyclopedia of DNA elements [36]. A total of 16 markers (Ctcf, Ezh239875, H2az, H3k4me1, H3k4me2, H3k4me3, H3k9me3, H3k27ac, H3k9ac, H3k9me1, H3k27ac, H3k27me3, H3k36me3, H3k79me2, H4k20me1, and Pol2b) were assessed. We further supplied the ChIP-seq data of H3K4me3, H3K4me1, H3K27me3, H3K36me3, H3K27ac, H3K9me3 histone modification marker from synovial fibroblast in functional annotations [38]. For the SNPs shared with RA and CVDs, we overlapped them with these potential genome regulatory regions, respectively. All genome data used in this section were based on hg19 genome assembly.

For gene mapping, independent variants were first annotated by using ANNOVAR [39]. We obtained the data for eQTL and chromatin interaction mappings, from the Functional Mapping and Annotation of Genome-wide Association Studies (FUMA, <https://fuma.ctglab.nl/>). Specifically, we selected the gene expression data from eQTLGen Consortium , and five tissue types (i.e., artery coronary, artery tibial, whole blood, atrial appendage, and left ventricle) based on the Genotype-Tissue Expression project (GTEx v8) for eQTL analysis [40]. For chromatin interaction mappings, the long-range interactions (Hi-C) dataset originating from GM12878 was used. We also supplied the Hi-C data from synovial fibroblast [38]. For genetic variants mapping in coding/non-coding regions (i.e., exons and introns), we reported the candidate gene identified by ANNOVAR. If the genetic variant was mapped in the upstream, downstream, or intergenic regions, the most “likely” gene was assigned based on Hi-C (marked by "c”) or eQTLs (marked by "e”) mapping results. If there was no overlap between genes identified by ANNOVAR and genes modulated by eQTLs, genes with positional and chromatin interaction mapping evidence were flagged as putative candidates for the disease.

1. **Supplementary Figures**


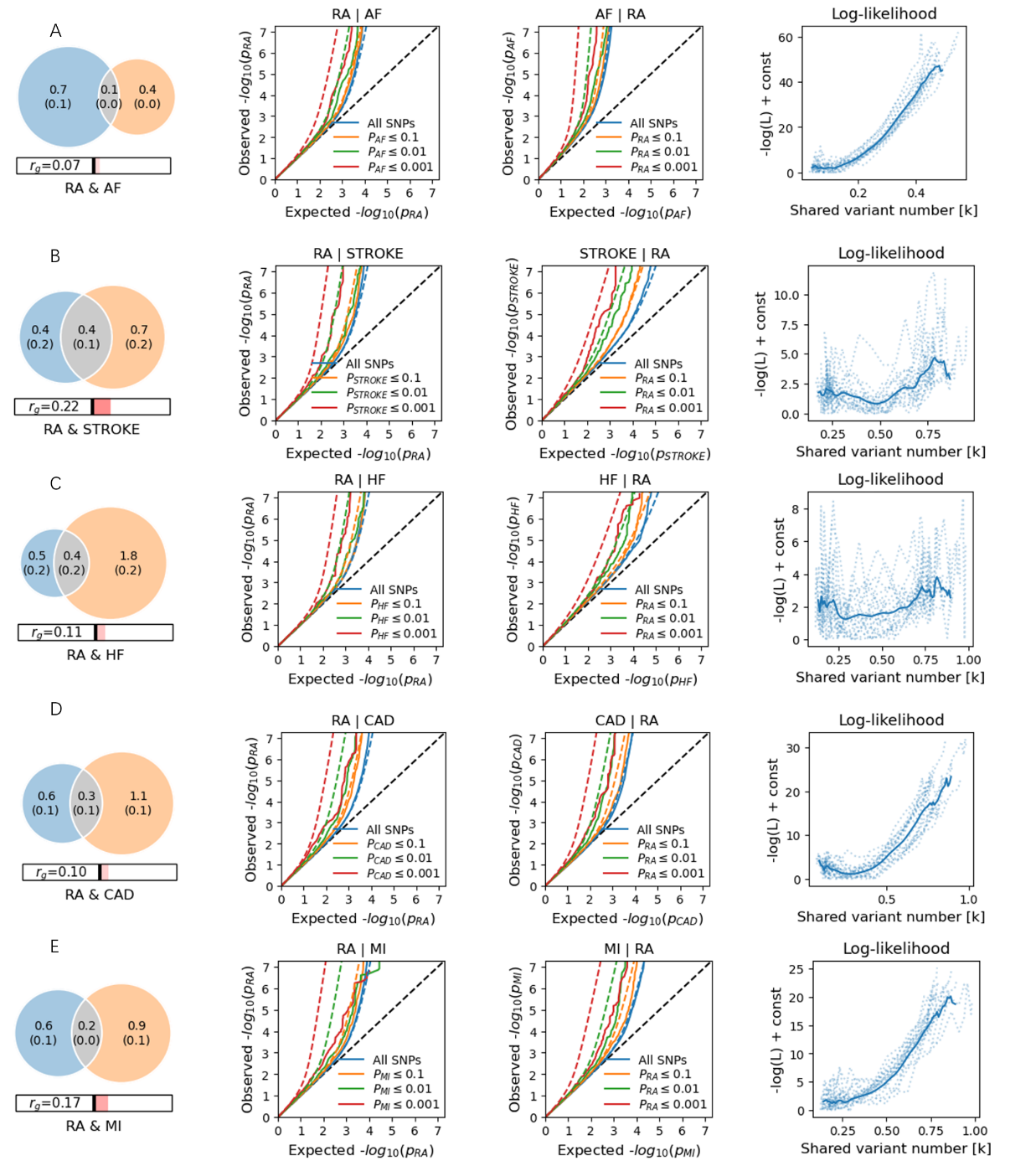


**Supplementary Figure 1. Polygenic overlap between rheumatoid arthritis and cardiovascular diseases: (A) RA and AF; (B) RA and stroke; (C) RA and HF; and (D) RA and CAD .** Each sub-figure contained Venn Diagrams, conditional Q-Q plots, and negative log-likelihood plot, respectively. Venn diagrams showed the polygenic overlap (gray) between RA (blue) and the corresponding CVD phenotype (orange). The numbers in the Venn diagram indicate the estimated number of variants (in thousands) explained 90% of heritability in the corresponding phenotype, followed by the standard error. Conditional Q–Q plots of observed versus expected -log_10_ *P*-values in the primary trait as a function of significance of association with a secondary trait at the different threshold of *P*-values. The Black dotted line indicate the null hypothesis. In the negative log-likelihood plot, minus log-likelihood is calculated for the bivariate model as a function of parameter. Abbreviations: AF, atrial fibrillation; CAD, coronary artery disease; HF, heart failure; RA, rheumatoid arthritis.

**Supplementary Table Caption**

**Table S1.** The ICD-9 diagnosis codes, ICD-10 diagnosis codes and self-reported codes in the UK Biobank for rheumatoid arthritis and cardiovascular diseases.

**Table S2.** Detailed information of summary statistics of rheumatoid arthritis and cardiovascular diseases.

**Table S3.** Basic characteristics of individuals included in the RA-CVD cohort.

**Table S4.** Propensity score and stratified analyses of associations between rheumatoid arthritis and cardiovascular diseases risk.

**Table S5.** Basic characteristics of individuals included in the CVD-RA cohort.

**Table S6.** Propensity score and stratified analyses of associations between cardiovascular diseases and rheumatoid arthritis risk.

**Table S7.** The regions with nominally significant genetic correlation identified from LAVA analysis.

**Table S8.** The results of model fit in MeXiR analysis.

**Table S9.** The independent pleiotropic loci based on conjFDR analyses.

**Table S10.** The independent pleiotropic loci based on ASSET analyses.

**Table S11.** The results of histone modification and enhancer enrichment for lead SNPs.

**Table S12.** The results of summary-based Mendelian randomization analyses and colocalization results.

**Table S13.** The enriched GO terms of the mapped genes.

**Table S14.** The enriched KEGG pathway terms of the mapped genes.

**Table S15.** The effects of different combinations of rheumatoid arthritis/cardiovascular diseases status and overlapped modifiable risk factors (diastolic blood pressure).

**The full list of MEGASTROKE authors**

We gratefully acknowledge the contributing studies and databases (MEGASTROKE project) that made GWAS summary data available. We acknowledge the contributions of Rainer Malik, Ganesh Chauhan, Matthew Traylor, Muralidharan Sargurupremraj, Yukinori Okada, Aniket Mishra, Loes Rutten-Jacobs, Anne-Katrin Giese, Sander W. van der Laan, Solveig Gretarsdottir, Christopher D, Anderson, Michael Chong, Hieab H. H. Adams, Tetsuro Ago, Peter Almgren, Philippe Amouyel, Hakan Ay, Traci M. Bartz, Oscar R. Benavente, Steve Bevan, Giorgio B. Boncoraglio, Robert D. Brown, Jr, Adam S. Butterworth, Caty Carrera, Cara L. Carty, Daniel I. Chasman, Wei-Min Chen, John W. Cole, Adolfo Correa, Ioana Cotlarciuc, Carlos Cruchaga, John Danesh, Paul I. W. de Bakker, Anita L. DeStefano, Marcel den Hoed, Qing Duan, Stefan T. Engelter, Guido J. Falcone, Rebecca F. Gottesman, Raji P. Grewal, Vilmundur Gudnason, Stefan Gustafsson, Jeffrey Haessler, Tamara B. Harris, Ahamad Hassan, Aki S. Havulinna, Susan R. Heckbert, Elizabeth G. Holliday, George Howard, Fang-Chi Hsu, Hyacinth I. Hyacinth, M. Arfan Ikram, Erik ingelsson, Marguerite R. Irvin, Xueqiu Jian, Jordi Jimenez-Conde, Julie A. Johnson, J. Wouter Jukema, Masahiro Kanai, Keith L. Keene, Brett M. Kissela, Dawn O. Kleindorfer, Charles Kooperberg, Michiaki Kubo, Leslie A. Lange, Carl D. Langefeld, Claudia Langenberg, Lenore J. Launer, Jin-Moo Lee, Robin Lemmens, Didier Leys, Cathryn M. Lewis, Wei-Yu Lin, Arne G. Lindgren, Erik Lorentzen, Patrik K. Magnusson, Jane Maguire, Ani Manichaikul, Patrick F. McArdle, James F. Meschia, Braxton D. Mitchell, Thomas H. Mosley, Michael A. Nalls, Toshiharu Ninomiya, Martin J. O’Donnell, Bruce M. Psaty, SaraL. Pulit, Kristiina Rannikmäe, Alexander P. Reiner, Kathryn M. Rexrode,

Kenneth Rice, Stephen S. Rich, Paul M. Ridker, Natalia S. Rost, Peter M. Rothwell, Jerome I. Rotter, Tatjana Rundek, Ralph L. Sacco, Saori Sakaue, Michele M. Sale, Veikko Salomaa, Bishwa R. Sapkota, Reinhold Schmidt, Carsten O. Schmidt, Ulf Schminke, Pankaj Sharma, Agnieszka Slowik, Cathie L. M. Sudlow, Christian Tanislav, Turgut Tatlisumak, Kent D. Taylor, Vincent N. S. Thijs, Gudmar Thorleifsson, Unnur Thorsteinsdottir, Steffen Tiedt, Stella Trompet, Christophe Tzourio, Cornelia M. van Duijn, Matthew Walters, Nicholas J. Wareham, Sylvia Wassertheil-Smoller, James G. Wilson, Kerri L. Wiggins, Qiong Yang, Salim Yusuf, AFGen Consortium, Cohorts for Heart and Aging Research in Genomic Epidemiology (CHARGE) Consortium, International Genomics of Blood Pressure(iGEN-BP) Consortium, INVENT Consortium, STARNET,

Joshua C. Bis, Tomi Pastinen, Arno Ruusalepp, Eric E. Schadt, Simon Koplev, Johan L. M. Björkegren, Veronica Codoni, Mete Civelek, Nicholas L. Smith, David A. Tregouet, Ingrid E. Christophersen, Carolina Roselli, Steven A. Lubitz, Patrick T. Ellinor, E. Shyong Tai, Jaspal S. Kooner, Norihiro Kato, Jiang He, Pim van der Harst, Paul Elliott, John C. Chambers, Fumihiko Takeuchi, Andrew D. Johnson, BioBank Japan Cooperative Hospital Group, COMPASS Consortium, EPiC-CVD Consortium, EPiC-interAct Consortium, International Stroke Genetics Consortium (ISGC), METASTROKE Consortium, Neurology Working Group of the CHARGE Consortium, NiNDS Stroke Genetics Network (SiGN), UK Young Lacunar DNA Study, MEGASTROKEConsortium, Dharambir K. Sanghera, Olle Melander, Christina Jern, Daniel Strbian, Israel Fernandez-Cadenas,W. T. Longstreth, Jr, Arndt Rolfs, Jun Hata, Daniel Woo, Jonathan Rosand, Guillaume Pare, Jemma C. Hopewell, Danish Saleheen, Kari Stefansson, Bradford B. Worrall, Steven J. Kittner, Sudha Seshadri, Myriam Fornage, Hugh S. Markus, Joanna M. M. Howson, Yoichiro Kamatani, Stephanie Debette, and Martin Dichgans

**Table S1**. The ICD-9 diagnosis codes, ICD-10 diagnosis codes and self-reported codes in the UK Biobank for rheumatoid arthritis and cardiovascular diseases.

| Phenotypes | ICD-9 diagnosis codes | ICD-10 diagnosis codes | Self-report codes |
| --- | --- | --- | --- |
| Rheumatoid arthritis | 714.X | M05.X - M06.X; M08.X | 1464 |
| Stroke | 430.X - 434.X, 436.X | I60.X, I61.X, I63.X, I64 | 1081; 1086; 1491; 1583 |
| Coronary artery disease | 410.X, 411.X, 412.X, 414.0, 414.8, 414.9 | I21.X - I24.X, I25.1, I25.2, I25.5, I25.6, I25.8, I25.9 | 1075 |
| Atrial fibrillation | 427.3 | I48 | 1471 |
| Heart failure | 428.X | I50.X | 1076 |

**Table S2.** Detailed information of summary statistics of rheumatoid arthritis and cardiovascular diseases.

| Phenotypes | Ancestry | Population | | Total sample | Number of SNPs in summary data | GWAS reference (PMID) |
| --- | --- | --- | --- | --- | --- | --- |
|  |  | Case | Control |  |  |  |
| Rheumatoid arthritis | European | 14361 | 43963 | 58324 | 8747961 | 24390342 |
| Atrial fibrillation | European | 60620 | 970216 | 1030836 | 29992370 | 30061737 |
| Coronary artery disease | European | 71602 | 260875 | 332477 | 9026567 | 28714975 |
| Heart failure | European | 47309 | 930014 | 977323 | 8281262 | 31919418 |
| Stroke | European | 40585 | 406111 | 446696 | 8255860 | 29531354 |

**Table S3.** Basic characteristics of individuals included in the RA-CVD cohort.

| Characteristics | Individuals without RA | Individuals with RA | *P* |
| --- | --- | --- | --- |
|  | (n = 432055) | (n = 9041) |  |
| CVD events, n | 47851 | 1467 |  |
| Incidence density (95% CI) (1000 person-years) | 10.36 (10.27, 10.45) | 15.33 (14.56, 16.14) |  |
| Age | 56.4±8.0 | 59.0±7.2 | < 2.2×10^-16^ |
| Sex (male) (%) | 190673(44.1) | 2629 (29.1) | < 2.2×10^-16^ |
| BMI (kg/m^2^) (%)^*^ |  |  |  |
| Underweight | 9283 (2.1) | 194 (2.1) | < 2.2×10^-16^ |
| Normal | 140785 (32.6) | 2447 (27.1) |  |
| Overweight | 180292 (41.7) | 3510 (38.8) |  |
| Obesity | 99937 (23.1) | 2808 (31.1) |  |
| Missing | 1758 (0.4) | 82 (0.9) |  |
| Smoking status (%) |  |  |  |
| Never | 237589 (55.0) | 4221 (46.7) | < 2.2×10^-16^ |
| Previous | 148533 (34.4) | 3619 (40.0) |  |
| Current | 44511 (10.3) | 1148 (12.7) |  |
| Missing | 1422 (0.3) | 53 (0.6) |  |
| Alcohol drinking frequency (%) |  |  |  |
| Never | 27524 (6.4) | 1080 (11.9) | < 2.2×10^-16^ |
| Special occasions only | 45971 (10.6) | 1482 (16.4) |  |
| One to three times a month | 48500 (11.2) | 1085 (12.0) |  |
| Once or twice a week | 114351 (26.5) | 2260 (25.0) |  |
| Three or four times a week | 104241 (24.1) | 1648 (18.2) |  |
| Daily or almost daily | 91174 (21.1) | 1482 (16.4) |  |
| Missing | 294 (0.1) | 4 (0.0) |  |
| Education level (%)^#^ |  |  |  |
| High level | 69192 (16.0) | 2467 (27.3) | < 2.2×10^-16^ |
| Middle level | 193251 (44.7) | 3894 (43.1) |  |
| Other qualification | 162734 (37.7) | 2496 (27.6) |  |
| Missing | 6878 (1.6) | 184 (2) |  |
| Hypertension (%) | 101106 (23.4) | 2854 (31.6) | < 2.2×10^-16^ |
| Type 2 Diabetes (%) | 6848 (1.6) | 304 (3.4) | < 2.2×10^-16^ |
| Total physical activity (MET hrs/week), median (IQR) | 25.03 (9.7, 53.8) | 19.3 (4.87, 49.2) | < 2.2×10^-16^ |
| Total cholesterol (log2) | 2.50±0.28 | 2.49±0.30 | 0.0045 |

^#^The educational level was classified as high level (individuals obtained college or university degree and profession qualifications), middle level (individuals obtained A levels/AS levels, O levels/GCSEs, CSEs, NVQ or HND or HNC), other qualification and missing data.umatoid arthritis cohort from the UK Biobank.

Abbreviations: CVD, cardiovascular diseases; RA, rheumatoid arthritis.

**Table S4.** Propensity score and stratified analyses of associations between rheumatoid arthritis and cardiovascular diseases risk.

|  | Model 1* | | |  | Model 2^#^ | | |
| --- | --- | --- | --- | --- | --- | --- | --- |
| Outcomes | HR | 95% CI | *P* |  | HR | 95% CI | *P* |
| **Male** |  |  |  |  |  |  |  |
| Cardiovascular diseases | 1.21 | 1.12-1.32 | 4.78×10^-6^ |  | 1.11 | 1.02-1.21 | 0.020 |
| Atrial fibrillation | 1.28 | 1.14-1.44 | 2.91×10^-5^ |  | 1.19 | 1.05-1.34 | 0.006 |
| Coronary artery disease | 1.15 | 1.02-1.29 | 0.017 |  | 1.05 | 0.93-1.18 | 0.439 |
| Heart failure | 1.63 | 1.38-1.93 | 1.15×10^-8^ |  | 1.40 | 1.18-1.66 | 1.29×10^4^ |
| Stroke | 1.27 | 1.03- 1.58 | 0.029 |  | 1.16 | 0.92-1.45 | 0.203 |
| **Female** |  |  |  |  |  |  |  |
| Cardiovascular diseases | 1.46 | 1.37-1.56 | 1.02×10^-28^ |  | 1.27 | 1.19-1.36 | 1.86×10^-11^ |
| Atrial fibrillation | 1.31 | 1.18-1.46 | 2.66×10^-7^ |  | 1.16 | 1.04-1.30 | 6.56×10^-3^ |
| Coronary artery disease | 1.52 | 1.39-1.67 | 3.00×10^-18^ |  | 1.29 | 1.17-1.42 | 6.33×10^-7^ |
| Heart failure | 1.96 | 1.72-2.25 | 5.72×10^-23^ |  | 1.65 | 1.44-1.90 | 1.50×10^-12^ |
| Stroke | 1.29 | 1.09-1.54 | 3.54×10^-03^ |  | 1.16 | 0.97-1.39 | 0.113 |
| **Age<60** |  |  |  |  |  |  |  |
| Cardiovascular diseases | 1.86 | 1.69-2.05 | 4.01×10^-35^ |  | 1.49 | 1.35-1.65 | 1.70×10^-14^ |
| Atrial fibrillation | 1.84 | 1.56-2.16 | 1.59×10^-13^ |  | 1.51 | 1.27-1.79 | 2.19×10^-6^ |
| Coronary artery disease | 1.83 | 1.60-2.08 | 4.42×10^-19^ |  | 1.45 | 1.26-1.66 | 1.07×10^-7^ |
| Heart failure | 2.78 | 2.25-3.44 | 2.01×10^-21^ |  | 2.16 | 1.74-2.68 | 2.11×10^-12^ |
| Stroke | 1.64 | 1.25-2.14 | 3.60×10^-4^ |  | 1.30 | 0.97-1.73 | 0.077 |
| **Age≥60** |  |  |  |  |  |  |  |
| Cardiovascular diseases | 1.30 | 1.22-1.38 | 3.39×10^-17^ |  | 1.17 | 1.10-1.25 | 1.17×10^-6^ |
| Atrial fibrillation | 1.28 | 1.17-1.39 | 6.21×10^-8^ |  | 1.17 | 1.06-1.28 | 1.06×10^-3^ |
| Coronary artery disease | 1.28 | 1.18- 1.40 | 1.77×10^-8^ |  | 1.13 | 1.03-1.24 | 7.08×10^-3^ |
| Heart failure | 1.74 | 1.54-1.96 | 2.21×10^-19^ |  | 1.48 | 1.30-1.67 | 9.35×10^-10^ |
| Stroke | 1.28 | 1.09-1.49 | 2.08×10^-3^ |  | 1.17 | 1.00-1.38 | 0.052 |
| **Excluding incident cases in the first follow-up year** | |  |  |  |  |  |  |
| Cardiovascular diseases | 1.39 | 1.32-1.47 | 2.90×10^-34^ |  | 1.24 | 1.17-1.31 | 1.73×10^-14^ |
| Atrial fibrillation | 1.32 | 1.22-1.42 | 7.75×10^-12^ |  | 1.20 | 1.10-1.30 | 1.99×10^-5^ |
| Coronary artery disease | 1.39 | 1.29-1.49 | 3.03×10^-18^ |  | 1.22 | 1.13-1.32 | 3.77×10^-7^ |
| Heart failure | 1.85 | 1.67-2.06 | 1.87×10^-30^ |  | 1.58 | 1.42-1.76 | 1.78×10^-16^ |
| Stroke | 1.34 | 1.17-1.53 | 2.42×10^-5^ |  | 1.21 | 1.05-1.39 | 0.008 |
| **Excluding individuals with kinship** |  |  |  |  |  |  |  |
| Cardiovascular diseases | 1.37 | 1.28-1.47 | 4.56×10^-21^ |  | 1.22 | 1.14-1.31 | 8.59×10^-9^ |
| Atrial fibrillation | 1.32 | 1.22-1.42 | 7.75×10^-12^ |  | 1.20 | 1.10-1.30 | 1.99×10^-5^ |
| Coronary artery disease | 1.39 | 1.29-1.49 | 3.03×10^-18^ |  | 1.22 | 1.13-1.32 | 3.77×10^-7^ |
| Heart failure | 1.85 | 1.67-2.06 | 1.87×10^-30^ |  | 1.58 | 1.42-1.76 | 1.78×10^-16^ |
| Stroke | 1.34 | 1.17-1.53 | 2.42×10^-5^ |  | 1.21 | 1.05-1.39 | 0.008 |
| **Propensity score analysis (1:4)** |  |  |  |  |  |  |  |
| Cardiovascular diseases | 1.30 | 1.22-1.38 | 3.20×10^-18^ |  | 1.16 | 1.09-1.24 | 1.45×10^-6^ |
| Atrial fibrillation | 1.21 | 1.11-1.32 | 2.46×10^-5^ |  | 1.09 | 0.99-1.19 | 0.072 |
| Coronary artery disease | 1.29 | 1.19-1.40 | 1.17×10^-9^ |  | 1.14 | 1.05-1.24 | 0.003 |
| Heart failure | 1.74 | 1.54-1.97 | 5.97×10^-19^ |  | 1.49 | 1.31-1.69 | 1.13×10^-9^ |
| Stroke | 1.32 | 1.14-1.54 | 3.33×10^-4^ |  | 1.22 | 1.03-1.43 | 0.018 |

*Model 1: adjusted for sex and/or age.

^#^Model 2: adjusted for sex, age, BMI, smoking status, alcohol drinking frequency, education levels, physical activity, hypertension, type 2 diabetes and total cholesterol.

**Table S5.** Basic characteristics of individuals included in the CVD-RA cohort.

| Characteristics | Individuals without CVD | Individuals with CVD | *P* |
| --- | --- | --- | --- |
|  | (n = 388003) | (n = 78282) |  |
| RA events, n | 3707 | 1012 |  |
| Incidence density (95% CI) (1000 person-years) | 0.86 (0.83, 0.89) | 1.16 (1.09, 1.23) |  |
| Age | 55.8±8.0 | 61.1±6.5 | < 2.2×10^-16^ |
| Sex (male) (%) | 163383 (42.1) | 49828 (63.7) | < 2.2×10^-16^ |
| BMI (kg/m^2^) (%) |  |  |  |
| Underweight | 8364 (2.2) | 1556 (2.0) | < 2.2×10^-16^ |
| Normal | 131020 (33.8) | 16392 (20.9) |  |
| Overweight | 161426 (41.6) | 33305 (42.5) |  |
| Obesity | 85704 (22.1) | 26451 (33.8) |  |
| Missing | 1489 (0.4) | 578 (0.7) |  |
| Smoking status (%) |  |  |  |
| Never | 217605 (56.1) | 33505 (42.8) | < 2.2×10^-16^ |
| Previous | 130549 (33.6) | 34276 (43.8) |  |
| Current | 38637 (10) | 10070 (12.9) |  |
| Missing | 1212 (0.3) | 431 (0.6) |  |
| Alcohol drinking frequency (%) |  |  |  |
| Never | 24012 (6.2) | 7111 (9.1) | < 2.2×10^-16^ |
| Special occasions only | 40738 (10.5) | 9576 (12.2) |  |
| One to three times a month | 44007 (11.3) | 7993 (10.2) |  |
| Once or twice a week | 103468 (26.7) | 19224 (24.6) |  |
| Three or four times a week | 94648 (24.4) | 16745 (21.4) |  |
| Daily or almost daily | 80881 (20.8) | 17542 (22.4) |  |
| Missing | 249 (0.1) | 91 (0.1) |  |
| Education level (%)* |  |  |  |
| High level | 57873 (14.9) | 21405 (27.3) | < 2.2×10^-16^ |
| Middle level | 175223 (45.2) | 31760 (40.6) |  |
| Other qualification | 149003 (38.4) | 23361 (29.8) |  |
| Missing | 5904 (1.5) | 1756 (2.2) |  |
| Hypertension (%) | 83426 (21.5) | 36680 (46.9) | < 2.2×10^-16^ |
| Type 2 Diabetes (%) | 5102 (1.3) | 4933 (6.3) | < 2.2×10^-16^ |
| Total physical activity (MET hrs/week), median (IQR) | 25.1 (9.9, 53.8) | 23.1 (7.7, 53.0) | < 2.2×10^-16^ |
| Total cholesterol (log2) | 2.51±0.28 | 2.38±0.34 | < 2.2×10^-16^ |

*The educational level was classified as high level (individuals obtained college or university degree and profession qualifications), middle level (individuals obtained A levels/AS levels, O levels/GCSEs, CSEs, NVQ or HND or HNC), other qualification and missing data.

Abbreviations: CVD, cardiovascular diseases; RA, rheumatoid arthritis.

**Table S6.** Propensity score and stratified analyses of associations between cardiovascular diseases and rheumatoid arthritis risk.

|  | Model 1 * | | |  | Model 2^#^ | | |
| --- | --- | --- | --- | --- | --- | --- | --- |
| Exposures | HR | 95% CI | *P* |  | HR | 95% CI | *P* |
| **Male** |  |  |  |  |  |  |  |
| Cardiovascular diseases | 1.15 | 1.03-1.28 | 0.011 |  | 1.06 | 0.95-1.18 | 0.317 |
| Atrial fibrillation | 1.06 | 0.92-1.22 | 0.434 |  | 1.00 | 0.87-1.16 | 0.991 |
| Coronary artery disease | 1.20 | 1.06-1.35 | 0.003 |  | 1.10 | 0.97-1.24 | 0.129 |
| Heart failure | 1.04 | 0.85-1.28 | 0.697 |  | 0.93 | 0.75-1.14 | 0.462 |
| Stroke | 1.01 | 0.81-1.25 | 0.951 |  | 0.93 | 0.75-1.15 | 0.499 |
| **Female** |  |  |  |  |  |  |  |
| Cardiovascular diseases | 1.18 | 1.07-1.30 | 0.001 |  | 1.03 | 0.93-1.14 | 0.579 |
| Atrial fibrillation | 0.88 | 0.75-1.03 | 0.121 |  | 0.80 | 0.69-0.94 | 0.006 |
| Coronary artery disease | 1.36 | 1.20-1.54 | 9.73×10^-7^ |  | 1.16 | 1.02-1.31 | 0.020 |
| Heart failure | 1.05 | 0.85-1.31 | 0.646 |  | 0.87 | 0.7-1.08 | 0.206 |
| Stroke | 1.18 | 0.98-1.42 | 0.081 |  | 1.04 | 0.86-1.25 | 0.680 |
| **Age<60** |  |  |  |  |  |  |  |
| Cardiovascular diseases | 1.89 | 1.66-2.16 | 8.79×10^-22^ |  | 1.58 | 1.38-1.80 | 1.22×10^-11^ |
| Atrial fibrillation | 1.77 | 1.43-2.19 | 1.06×10^-7^ |  | 1.55 | 1.26-1.92 | 4.41×10^-5^ |
| Coronary artery disease | 2.10 | 1.79-2.46 | 5.30×10^-20^ |  | 1.71 | 1.45-2.00 | 6.39×10^-11^ |
| Heart failure | 1.70 | 1.23-2.34 | 0.001 |  | 1.30 | 0.94-1.80 | 0.113 |
| Stroke | 1.89 | 1.47-2.43 | 7.16×10^-7^ |  | 1.54 | 1.19-1.98 | 0.001 |
| **Age≥60** |  |  |  |  |  |  |  |
| Cardiovascular diseases | 1.07 | 0.99-1.17 | 0.103 |  | 0.97 | 0.89-1.06 | 0.562 |
| Atrial fibrillation | 0.91 | 0.81-1.03 | 0.137 |  | 0.85 | 0.76-0.96 | 0.011 |
| Coronary artery disease | 1.18 | 1.06-1.30 | 0.002 |  | 1.06 | 0.95-1.17 | 0.289 |
| Heart failure | 1.03 | 0.87-1.22 | 0.735 |  | 0.9 | 0.76-1.06 | 0.200 |
| Stroke | 0.99 | 0.84-1.17 | 0.921 |  | 0.91 | 0.77-1.07 | 0.252 |
| **Excluding incident cases in the first follow-up year** |  |  |  |  |  |  |  |
| Cardiovascular diseases | 1.19 | 1.10-1.28 | 4.35×10^-6^ |  | 1.06 | 0.99-1.15 | 0.100 |
| Atrial fibrillation | 1.00 | 0.90-1.11 | 0.975 |  | 0.93 | 0.84-1.04 | 0.188 |
| Coronary artery disease | 1.30 | 1.19-1.42 | 3.63×10^-9^ |  | 1.15 | 1.05-1.26 | 0.002 |
| Heart failure | 1.08 | 0.93-1.25 | 0.322 |  | 0.92 | 0.79-1.07 | 0.298 |
| Stroke | 1.11 | 0.96-1.28 | 0.147 |  | 1.00 | 0.86-1.15 | 0.964 |
| **Excluding individuals with kinship** |  |  |  |  |  |  |  |
| Cardiovascular diseases | 1.15 | 1.05-1.27 | 0.003 |  | 1.02 | 0.93-1.12 | 0.637 |
| Atrial fibrillation | 0.98 | 0.85-1.12 | 0.737 |  | 0.90 | 0.79-1.03 | 0.138 |
| Coronary artery disease | 1.25 | 1.12-1.4 | 1.06×10^-4^ |  | 1.09 | 0.98-1.23 | 0.114 |
| Heart failure | 0.98 | 0.80-1.19 | 0.817 |  | 0.83 | 0.68-1.01 | 0.060 |
| Stroke | 1.13 | 0.95-1.35 | 0.171 |  | 1.01 | 0.84-1.20 | 0.948 |
| **Propensity score analysis (1:4)** |  |  |  |  |  |  |  |
| Cardiovascular diseases | 1.17 | 1.08-1.25 | 3.44×10^-5^ |  | 1.05 | 0.97-1.13 | 0.205 |
| Atrial fibrillation | 0.98 | 0.88-1.08 | 0.645 |  | 0.91 | 0.82-1.01 | 0.076 |
| Coronary artery disease | 1.28 | 1.17-1.39 | 2.90×10^-8^ |  | 1.13 | 1.04-1.24 | 0.004 |
| Heart failure | 1.05 | 0.91-1.22 | 0.501 |  | 0.90 | 0.78-1.05 | 0.190 |
| Stroke | 1.10 | 0.96-1.27 | 0.178 |  | 0.99 | 0.86-1.15 | 0.944 |

*Model 1: adjusted for sex and/or age.

#Model 2: adjusted for sex, age, body mass index, smoking status, alcohol drinking frequency, education levels and physical activity.

**Table S7.** The regions with nominally significant genetic correlation identified from LAVA analysis.

| CHR | Start | End | No. of SNPs | No. of PCS | Trait 1 | Trait 2 | rho | rho.lower | rho.upper | r^2^ | r^2^.lower | r^2^.upper | *P* | FDR-*P* |
| --- | --- | --- | --- | --- | --- | --- | --- | --- | --- | --- | --- | --- | --- | --- |
| 1 | 22145630 | 22740287 | 1559 | 159 | RA | AF | 0.565 | 0.176 | 1.000 | 0.319 | 0.031 | 1.000 | 0.007 | 0.246 |
| 1 | 41977967 | 43512669 | 2453 | 172 | RA | AF | 0.769 | 0.224 | 1.000 | 0.591 | 0.053 | 1.000 | 0.010 | 0.250 |
| 1 | 53114187 | 54771293 | 3049 | 248 | RA | AF | 0.467 | 0.042 | 1.000 | 0.218 | 0.004 | 1.000 | 0.036 | 0.330 |
| 1 | 62778734 | 64344226 | 2826 | 227 | RA | AF | -0.521 | -1.000 | -0.109 | 0.272 | 0.014 | 1.000 | 0.016 | 0.270 |
| 1 | 113418038 | 114664387 | 1725 | 167 | RA | AF | 0.362 | 0.053 | 1.000 | 0.131 | 0.004 | 1.000 | 0.029 | 0.304 |
| 1 | 114664388 | 116207297 | 2608 | 224 | RA | AF | 0.266 | 0.011 | 0.528 | 0.071 | 0.001 | 0.279 | 0.040 | 0.337 |
| 1 | 117046312 | 118118667 | 1640 | 191 | RA | AF | 0.426 | 0.069 | 0.846 | 0.182 | 0.006 | 0.715 | 0.024 | 0.287 |
| 1 | 119408889 | 120345066 | 1698 | 157 | RA | AF | -0.791 | -1.000 | -0.191 | 0.626 | 0.039 | 1.000 | 0.014 | 0.270 |
| 1 | 149716038 | 151146407 | 1564 | 99 | RA | AF | 0.500 | 0.027 | 1.000 | 0.250 | 0.005 | 1.000 | 0.043 | 0.350 |
| 1 | 171044256 | 172465153 | 2861 | 180 | RA | AF | -0.624 | -1.000 | -0.129 | 0.390 | 0.021 | 1.000 | 0.018 | 0.270 |
| 1 | 173582512 | 175385220 | 2449 | 104 | RA | AF | -0.780 | -1.000 | -0.138 | 0.609 | 0.026 | 1.000 | 0.023 | 0.287 |
| 1 | 202583885 | 204092537 | 2770 | 276 | RA | AF | 0.271 | 0.050 | 0.501 | 0.074 | 0.003 | 0.251 | 0.017 | 0.270 |
| 1 | 212347583 | 213958292 | 2923 | 240 | RA | AF | -0.601 | -1.000 | -0.064 | 0.362 | 0.007 | 1.000 | 0.033 | 0.320 |
| 1 | 233639474 | 234365796 | 1848 | 181 | RA | AF | 0.781 | 0.201 | 1.000 | 0.610 | 0.042 | 1.000 | 0.012 | 0.255 |
| 2 | 3314203 | 4016104 | 1687 | 196 | RA | AF | -0.829 | -1.000 | -0.127 | 0.688 | 0.023 | 1.000 | 0.024 | 0.287 |
| 2 | 20064518 | 21084898 | 2089 | 231 | RA | AF | 0.638 | 0.135 | 1.000 | 0.408 | 0.020 | 1.000 | 0.016 | 0.270 |
| 2 | 36867390 | 37603360 | 1479 | 176 | RA | AF | -0.885 | -1.000 | -0.379 | 0.783 | 0.143 | 1.000 | 0.001 | 0.135 |
| 2 | 57952946 | 59251996 | 1660 | 171 | RA | AF | -0.896 | -1.000 | -0.167 | 0.802 | 0.035 | 1.000 | 0.020 | 0.286 |
| 2 | 65938003 | 67224027 | 2152 | 211 | RA | AF | 0.499 | 0.133 | 0.953 | 0.249 | 0.018 | 0.908 | 0.011 | 0.250 |
| 2 | 119635490 | 121073752 | 2262 | 197 | RA | AF | -0.609 | -1.000 | -0.147 | 0.371 | 0.023 | 1.000 | 0.013 | 0.255 |
| 2 | 141802665 | 142494980 | 1764 | 111 | RA | AF | -1.000 | -1.000 | -0.255 | 1.000 | 0.072 | 1.000 | 0.014 | 0.270 |
| 2 | 152497376 | 153999309 | 2639 | 166 | RA | AF | 0.917 | 0.254 | 1.000 | 0.841 | 0.068 | 1.000 | 0.011 | 0.250 |
| 2 | 215899571 | 217566011 | 3049 | 291 | RA | AF | -0.439 | -0.801 | -0.136 | 0.193 | 0.019 | 0.641 | 0.006 | 0.228 |
| 2 | 219678784 | 220771552 | 1538 | 170 | RA | AF | -0.586 | -0.963 | -0.285 | 0.343 | 0.081 | 0.927 | 0.000 | 0.064 |
| 2 | 229764919 | 230541092 | 1737 | 187 | RA | AF | 0.590 | 0.019 | 1.000 | 0.348 | 0.007 | 1.000 | 0.049 | 0.368 |
| 3 | 4109394 | 4835083 | 1622 | 220 | RA | AF | 0.601 | 0.138 | 1.000 | 0.361 | 0.020 | 1.000 | 0.014 | 0.270 |
| 3 | 12859210 | 14312007 | 3182 | 288 | RA | AF | 0.326 | 0.037 | 0.638 | 0.106 | 0.002 | 0.407 | 0.031 | 0.309 |
| 3 | 68319771 | 69784969 | 3033 | 225 | RA | AF | 0.522 | 0.146 | 1.000 | 0.272 | 0.022 | 1.000 | 0.008 | 0.246 |
| 3 | 124823007 | 125475722 | 1515 | 116 | RA | AF | -0.751 | -1.000 | -0.184 | 0.564 | 0.036 | 1.000 | 0.015 | 0.270 |
| 3 | 148638309 | 149998411 | 2580 | 251 | RA | AF | -0.552 | -1.000 | -0.134 | 0.305 | 0.019 | 1.000 | 0.011 | 0.251 |
| 3 | 186602046 | 187939199 | 2784 | 289 | RA | AF | 0.346 | 0.041 | 0.684 | 0.120 | 0.003 | 0.468 | 0.028 | 0.302 |
| 4 | 1529268 | 2468935 | 1708 | 183 | RA | AF | -0.739 | -1.000 | -0.237 | 0.546 | 0.057 | 1.000 | 0.006 | 0.228 |
| 4 | 40590542 | 41360937 | 1683 | 164 | RA | AF | 0.871 | 0.165 | 1.000 | 0.758 | 0.035 | 1.000 | 0.022 | 0.286 |
| 4 | 121393009 | 121981243 | 1490 | 92 | RA | AF | 1.000 | 0.155 | 1.000 | 1.000 | 0.037 | 1.000 | 0.029 | 0.304 |
| 4 | 126476843 | 127279171 | 1651 | 132 | RA | AF | -1.000 | -1.000 | -0.246 | 1.000 | 0.065 | 1.000 | 0.010 | 0.250 |
| 5 | 14910296 | 16592319 | 2325 | 201 | RA | AF | -0.725 | -1.000 | -0.253 | 0.526 | 0.064 | 1.000 | 0.003 | 0.188 |
| 5 | 60930754 | 62180368 | 1835 | 142 | RA | AF | 0.536 | 0.049 | 1.000 | 0.287 | 0.007 | 1.000 | 0.036 | 0.330 |
| 5 | 95117260 | 96467377 | 2278 | 195 | RA | AF | 0.706 | 0.266 | 1.000 | 0.499 | 0.071 | 1.000 | 0.004 | 0.199 |
| 5 | 132554691 | 134556570 | 3037 | 291 | RA | AF | 0.443 | 0.086 | 0.960 | 0.197 | 0.009 | 0.922 | 0.016 | 0.270 |
| 5 | 173606996 | 174662885 | 2528 | 280 | RA | AF | -0.379 | -0.725 | -0.079 | 0.144 | 0.007 | 0.526 | 0.015 | 0.270 |
| 6 | 3964073 | 4657086 | 1819 | 155 | RA | AF | 0.623 | 0.079 | 1.000 | 0.388 | 0.011 | 1.000 | 0.031 | 0.312 |
| 6 | 28666365 | 29529755 | 1972 | 71 | RA | AF | 0.556 | 0.154 | 1.000 | 0.309 | 0.025 | 1.000 | 0.011 | 0.250 |
| 6 | 30715007 | 31106493 | 2086 | 92 | RA | AF | 0.401 | 0.055 | 1.000 | 0.161 | 0.005 | 1.000 | 0.027 | 0.297 |
| 6 | 31106494 | 31250556 | 1713 | 41 | RA | AF | 0.499 | 0.129 | 1.000 | 0.249 | 0.017 | 1.000 | 0.012 | 0.255 |
| 6 | 31250557 | 31320268 | 1349 | 59 | RA | AF | 0.286 | 0.063 | 0.529 | 0.082 | 0.004 | 0.280 | 0.017 | 0.270 |
| 6 | 31427210 | 32208901 | 2079 | 149 | RA | AF | 0.246 | 0.046 | 0.462 | 0.060 | 0.002 | 0.214 | 0.017 | 0.270 |
| 6 | 32586785 | 32629239 | 575 | 96 | RA | AF | -0.318 | -0.498 | -0.145 | 0.101 | 0.021 | 0.248 | 0.000 | 0.064 |
| 6 | 36346354 | 37570051 | 2512 | 247 | RA | AF | -0.328 | -0.633 | -0.053 | 0.108 | 0.003 | 0.400 | 0.025 | 0.287 |
| 6 | 38688721 | 39785907 | 2609 | 213 | RA | AF | 0.716 | 0.056 | 1.000 | 0.513 | 0.012 | 1.000 | 0.039 | 0.337 |
| 6 | 43770627 | 44596897 | 1673 | 263 | RA | AF | -0.297 | -0.582 | -0.020 | 0.088 | 0.002 | 0.339 | 0.033 | 0.320 |
| 6 | 51826424 | 52598879 | 1643 | 180 | RA | AF | 0.501 | 0.023 | 1.000 | 0.251 | 0.003 | 1.000 | 0.049 | 0.368 |
| 6 | 87516323 | 88499745 | 1889 | 89 | RA | AF | 0.719 | 0.094 | 1.000 | 0.517 | 0.016 | 1.000 | 0.033 | 0.320 |
| 6 | 104951345 | 106053915 | 1653 | 155 | RA | AF | 0.572 | 0.034 | 1.000 | 0.327 | 0.006 | 1.000 | 0.035 | 0.326 |
| 6 | 132890695 | 134313643 | 2741 | 209 | RA | AF | -0.428 | -0.854 | -0.076 | 0.183 | 0.007 | 0.729 | 0.022 | 0.286 |
| 6 | 162254230 | 163336361 | 2708 | 194 | RA | AF | -0.646 | -1.000 | -0.021 | 0.417 | 0.007 | 1.000 | 0.044 | 0.350 |
| 7 | 27351287 | 28890886 | 3081 | 279 | RA | AF | 0.350 | 0.099 | 0.617 | 0.122 | 0.010 | 0.380 | 0.006 | 0.228 |
| 7 | 35354136 | 36507690 | 2345 | 201 | RA | AF | 0.623 | 0.052 | 1.000 | 0.388 | 0.009 | 1.000 | 0.036 | 0.328 |
| 7 | 53176524 | 54086987 | 2159 | 120 | RA | AF | 0.850 | 0.315 | 1.000 | 0.723 | 0.099 | 1.000 | 0.005 | 0.219 |
| 7 | 90660832 | 92530165 | 1985 | 184 | RA | AF | -0.456 | -1.000 | -0.071 | 0.208 | 0.007 | 1.000 | 0.027 | 0.300 |
| 7 | 99465541 | 100849307 | 1568 | 165 | RA | AF | -0.820 | -1.000 | -0.252 | 0.672 | 0.065 | 1.000 | 0.009 | 0.249 |
| 7 | 119568237 | 121042050 | 1714 | 112 | RA | AF | 0.981 | 0.151 | 1.000 | 0.962 | 0.033 | 1.000 | 0.031 | 0.309 |
| 7 | 133807217 | 134943221 | 2421 | 158 | RA | AF | -0.652 | -1.000 | -0.138 | 0.425 | 0.022 | 1.000 | 0.019 | 0.274 |
| 7 | 138755108 | 140217630 | 2368 | 252 | RA | AF | 0.661 | 0.349 | 1.000 | 0.437 | 0.122 | 1.000 | 0.000 | 0.049 |
| 7 | 146574328 | 147352171 | 1992 | 133 | RA | AF | 0.909 | 0.301 | 1.000 | 0.827 | 0.091 | 1.000 | 0.006 | 0.228 |
| 7 | 147352172 | 148622913 | 2737 | 248 | RA | AF | 0.669 | 0.125 | 1.000 | 0.448 | 0.019 | 1.000 | 0.017 | 0.270 |
| 7 | 153241229 | 154407487 | 2032 | 234 | RA | AF | 0.383 | 0.013 | 0.921 | 0.147 | 0.003 | 0.850 | 0.043 | 0.350 |
| 8 | 21650454 | 22895018 | 2084 | 264 | RA | AF | 0.421 | 0.039 | 0.886 | 0.177 | 0.004 | 0.785 | 0.030 | 0.309 |
| 8 | 72013185 | 72917489 | 1885 | 216 | RA | AF | 0.803 | 0.124 | 1.000 | 0.645 | 0.023 | 1.000 | 0.023 | 0.287 |
| 8 | 89081178 | 90364149 | 2219 | 121 | RA | AF | -0.617 | -1.000 | -0.141 | 0.381 | 0.022 | 1.000 | 0.018 | 0.270 |
| 8 | 92876621 | 94999066 | 2857 | 238 | RA | AF | 0.724 | 0.211 | 1.000 | 0.524 | 0.047 | 1.000 | 0.010 | 0.250 |
| 8 | 128073655 | 129291118 | 2713 | 298 | RA | AF | -0.509 | -0.912 | -0.201 | 0.259 | 0.040 | 0.832 | 0.002 | 0.149 |
| 8 | 134509449 | 135313673 | 2195 | 235 | RA | AF | -0.562 | -1.000 | -0.072 | 0.316 | 0.009 | 1.000 | 0.029 | 0.303 |
| 9 | 17990566 | 18754196 | 1745 | 205 | RA | AF | 0.625 | 0.102 | 1.000 | 0.391 | 0.015 | 1.000 | 0.026 | 0.296 |
| 9 | 33194893 | 34704544 | 2478 | 154 | RA | AF | 0.443 | 0.063 | 0.917 | 0.196 | 0.006 | 0.842 | 0.025 | 0.287 |
| 9 | 37687113 | 38822977 | 2051 | 228 | RA | AF | -0.489 | -1.000 | 0.002 | 0.239 | 0.000 | 1.000 | 0.049 | 0.368 |
| 9 | 76197745 | 77862308 | 2669 | 211 | RA | AF | 1.000 | 0.412 | 1.000 | 1.000 | 0.171 | 1.000 | 0.001 | 0.086 |
| 9 | 86769887 | 87815449 | 2317 | 182 | RA | AF | -0.569 | -1.000 | -0.040 | 0.324 | 0.005 | 1.000 | 0.039 | 0.337 |
| 9 | 93441051 | 94175374 | 1572 | 180 | RA | AF | 0.871 | 0.083 | 1.000 | 0.758 | 0.020 | 1.000 | 0.034 | 0.320 |
| 9 | 136770927 | 137593527 | 1746 | 235 | RA | AF | 0.449 | 0.063 | 0.951 | 0.202 | 0.006 | 0.905 | 0.027 | 0.300 |
| 9 | 138202113 | 138995791 | 1770 | 259 | RA | AF | 0.581 | 0.235 | 1.000 | 0.338 | 0.055 | 1.000 | 0.002 | 0.135 |
| 10 | 3880323 | 4813182 | 2086 | 230 | RA | AF | 0.526 | 0.140 | 1.000 | 0.277 | 0.020 | 1.000 | 0.011 | 0.250 |
| 10 | 7659873 | 8771920 | 2794 | 273 | RA | AF | -0.557 | -1.000 | -0.074 | 0.310 | 0.008 | 1.000 | 0.028 | 0.303 |
| 10 | 77144962 | 78665481 | 2164 | 184 | RA | AF | 0.355 | 0.020 | 0.725 | 0.126 | 0.002 | 0.526 | 0.039 | 0.337 |
| 10 | 88443653 | 89971628 | 1907 | 182 | RA | AF | -0.853 | -1.000 | -0.287 | 0.728 | 0.083 | 1.000 | 0.005 | 0.228 |
| 10 | 92911507 | 94503299 | 2175 | 135 | RA | AF | -0.458 | -0.845 | -0.119 | 0.209 | 0.014 | 0.714 | 0.011 | 0.250 |
| 10 | 99367801 | 100337830 | 1772 | 204 | RA | AF | -0.622 | -1.000 | -0.226 | 0.386 | 0.051 | 1.000 | 0.003 | 0.188 |
| 10 | 116845214 | 118416049 | 2556 | 160 | RA | AF | -0.709 | -1.000 | -0.168 | 0.502 | 0.031 | 1.000 | 0.017 | 0.270 |
| 11 | 1857846 | 2477449 | 1486 | 192 | RA | AF | 0.362 | 0.027 | 0.746 | 0.131 | 0.003 | 0.557 | 0.035 | 0.328 |
| 11 | 15126768 | 16383386 | 2045 | 170 | RA | AF | -0.600 | -1.000 | -0.126 | 0.360 | 0.017 | 1.000 | 0.016 | 0.270 |
| 11 | 19780785 | 20686336 | 2103 | 269 | RA | AF | 0.424 | 0.122 | 0.811 | 0.180 | 0.015 | 0.657 | 0.008 | 0.246 |
| 11 | 36331190 | 37119024 | 1602 | 143 | RA | AF | -0.566 | -1.000 | -0.028 | 0.320 | 0.007 | 1.000 | 0.041 | 0.346 |
| 11 | 94384536 | 95327210 | 1608 | 183 | RA | AF | 0.472 | 0.140 | 0.867 | 0.223 | 0.020 | 0.752 | 0.007 | 0.234 |
| 12 | 23923799 | 25058714 | 2327 | 248 | RA | AF | 0.428 | 0.201 | 0.680 | 0.183 | 0.040 | 0.462 | 0.000 | 0.064 |
| 12 | 46037745 | 47499603 | 1958 | 158 | RA | AF | 0.645 | 0.154 | 1.000 | 0.416 | 0.026 | 1.000 | 0.015 | 0.270 |
| 12 | 68006683 | 68839661 | 1647 | 198 | RA | AF | 0.428 | 0.091 | 0.814 | 0.183 | 0.009 | 0.662 | 0.015 | 0.270 |
| 12 | 102966634 | 104140831 | 2225 | 217 | RA | AF | -0.527 | -1.000 | -0.062 | 0.278 | 0.008 | 1.000 | 0.028 | 0.301 |
| 12 | 121817510 | 123396634 | 2116 | 145 | RA | AF | 0.496 | 0.070 | 1.000 | 0.246 | 0.008 | 1.000 | 0.025 | 0.287 |
| 12 | 132957946 | 133841814 | 1438 | 128 | RA | AF | -0.517 | -1.000 | -0.028 | 0.267 | 0.005 | 1.000 | 0.041 | 0.344 |
| 13 | 24818334 | 25977140 | 2871 | 223 | RA | AF | -0.633 | -1.000 | -0.033 | 0.401 | 0.008 | 1.000 | 0.045 | 0.355 |
| 13 | 30068526 | 31433218 | 3036 | 275 | RA | AF | -0.419 | -0.889 | -0.065 | 0.176 | 0.005 | 0.791 | 0.021 | 0.286 |
| 13 | 40091624 | 41003943 | 1847 | 218 | RA | AF | -0.539 | -1.000 | -0.071 | 0.291 | 0.008 | 1.000 | 0.024 | 0.287 |
| 13 | 41003944 | 42037054 | 1523 | 132 | RA | AF | -0.409 | -0.811 | -0.062 | 0.167 | 0.005 | 0.658 | 0.022 | 0.286 |
| 13 | 43931932 | 45476446 | 2587 | 219 | RA | AF | -0.382 | -0.831 | -0.020 | 0.146 | 0.003 | 0.690 | 0.044 | 0.350 |
| 13 | 74437636 | 75882384 | 2781 | 241 | RA | AF | 0.352 | 0.011 | 0.745 | 0.124 | 0.002 | 0.555 | 0.042 | 0.350 |
| 13 | 98527336 | 99402294 | 1974 | 246 | RA | AF | -0.462 | -0.915 | -0.106 | 0.213 | 0.012 | 0.837 | 0.013 | 0.255 |
| 13 | 103050418 | 104378435 | 3128 | 248 | RA | AF | 0.504 | 0.083 | 1.000 | 0.254 | 0.010 | 1.000 | 0.023 | 0.287 |
| 13 | 109156766 | 109813576 | 1642 | 168 | RA | AF | -0.720 | -1.000 | -0.256 | 0.518 | 0.066 | 1.000 | 0.004 | 0.203 |
| 14 | 29029225 | 30831154 | 2402 | 246 | RA | AF | 0.682 | 0.328 | 1.000 | 0.465 | 0.108 | 1.000 | 0.001 | 0.085 |
| 14 | 68976913 | 69794542 | 1670 | 202 | RA | AF | -0.673 | -1.000 | -0.190 | 0.452 | 0.038 | 1.000 | 0.009 | 0.249 |
| 14 | 75699502 | 76694201 | 1575 | 203 | RA | AF | 0.970 | 0.277 | 1.000 | 0.941 | 0.083 | 1.000 | 0.010 | 0.250 |
| 14 | 89482884 | 90662836 | 2237 | 251 | RA | AF | -0.727 | -1.000 | -0.276 | 0.528 | 0.077 | 1.000 | 0.003 | 0.188 |
| 14 | 90662837 | 92101228 | 2596 | 217 | RA | AF | -0.580 | -1.000 | -0.097 | 0.337 | 0.012 | 1.000 | 0.021 | 0.286 |
| 15 | 32177321 | 33484267 | 1965 | 226 | RA | AF | -0.395 | -0.783 | -0.051 | 0.156 | 0.004 | 0.613 | 0.025 | 0.287 |
| 15 | 36583328 | 37962915 | 2575 | 261 | RA | AF | -0.268 | -0.536 | -0.009 | 0.072 | 0.001 | 0.287 | 0.040 | 0.337 |
| 15 | 37962916 | 39238840 | 2415 | 241 | RA | AF | 0.340 | 0.054 | 0.651 | 0.116 | 0.004 | 0.424 | 0.022 | 0.286 |
| 15 | 40604781 | 42332685 | 2443 | 193 | RA | AF | 0.471 | 0.129 | 0.873 | 0.222 | 0.017 | 0.762 | 0.009 | 0.249 |
| 15 | 47693317 | 49260006 | 2001 | 186 | RA | AF | -0.450 | -0.809 | -0.136 | 0.203 | 0.019 | 0.655 | 0.006 | 0.228 |
| 15 | 69089816 | 70767983 | 2776 | 282 | RA | AF | 0.343 | 0.156 | 0.537 | 0.118 | 0.024 | 0.289 | 0.001 | 0.086 |
| 15 | 98828262 | 99925972 | 2503 | 276 | RA | AF | -0.568 | -1.000 | -0.171 | 0.322 | 0.030 | 1.000 | 0.006 | 0.228 |
| 16 | 3379997 | 4816145 | 2251 | 264 | RA | AF | 0.403 | 0.146 | 0.684 | 0.163 | 0.021 | 0.468 | 0.003 | 0.188 |
| 16 | 12793150 | 13893407 | 2826 | 232 | RA | AF | -1.000 | -1.000 | -0.174 | 1.000 | 0.042 | 1.000 | 0.018 | 0.270 |
| 16 | 29043178 | 31384210 | 1817 | 177 | RA | AF | -0.441 | -1.000 | -0.029 | 0.194 | 0.004 | 1.000 | 0.042 | 0.350 |
| 16 | 54866096 | 55907389 | 2380 | 225 | RA | AF | 0.388 | 0.014 | 0.826 | 0.151 | 0.003 | 0.683 | 0.044 | 0.350 |
| 16 | 58508979 | 59885499 | 3086 | 196 | RA | AF | 0.836 | 0.335 | 1.000 | 0.699 | 0.113 | 1.000 | 0.002 | 0.160 |
| 16 | 62607091 | 64207176 | 2590 | 199 | RA | AF | 0.545 | 0.012 | 1.000 | 0.297 | 0.004 | 1.000 | 0.046 | 0.359 |
| 16 | 71062713 | 72089511 | 1455 | 103 | RA | AF | 0.425 | 0.028 | 0.923 | 0.181 | 0.003 | 0.852 | 0.040 | 0.337 |
| 16 | 83778372 | 84440154 | 2294 | 311 | RA | AF | -0.682 | -1.000 | -0.293 | 0.465 | 0.086 | 1.000 | 0.001 | 0.093 |
| 16 | 86058598 | 86748867 | 2333 | 326 | RA | AF | -0.342 | -0.619 | -0.090 | 0.117 | 0.008 | 0.383 | 0.010 | 0.250 |
| 17 | 9619357 | 10572617 | 2276 | 261 | RA | AF | -0.563 | -1.000 | -0.102 | 0.317 | 0.012 | 1.000 | 0.021 | 0.286 |
| 17 | 10572618 | 11778164 | 2295 | 257 | RA | AF | -0.474 | -1.000 | -0.078 | 0.224 | 0.008 | 1.000 | 0.022 | 0.286 |
| 17 | 32356062 | 33614189 | 2367 | 239 | RA | AF | -0.566 | -1.000 | -0.155 | 0.320 | 0.025 | 1.000 | 0.008 | 0.247 |
| 17 | 37361179 | 38880481 | 1843 | 163 | RA | AF | -0.350 | -0.712 | -0.023 | 0.122 | 0.002 | 0.508 | 0.038 | 0.337 |
| 17 | 58652810 | 60453120 | 1835 | 193 | RA | AF | -0.469 | -1.000 | -0.037 | 0.220 | 0.004 | 1.000 | 0.036 | 0.330 |
| 17 | 70495120 | 71466953 | 2111 | 299 | RA | AF | -0.304 | -0.599 | -0.022 | 0.093 | 0.002 | 0.358 | 0.034 | 0.320 |
| 18 | 10719 | 848350 | 1857 | 254 | RA | AF | 0.693 | 0.121 | 1.000 | 0.480 | 0.018 | 1.000 | 0.022 | 0.286 |
| 18 | 11872227 | 12735558 | 1895 | 158 | RA | AF | -0.811 | -1.000 | -0.197 | 0.657 | 0.041 | 1.000 | 0.014 | 0.270 |
| 18 | 24088164 | 25305260 | 2169 | 237 | RA | AF | -0.772 | -1.000 | -0.414 | 0.596 | 0.171 | 1.000 | 0.000 | 0.041 |
| 18 | 28792735 | 30401128 | 2889 | 222 | RA | AF | 0.423 | 0.022 | 1.000 | 0.179 | 0.003 | 1.000 | 0.046 | 0.359 |
| 18 | 55809316 | 56832312 | 2211 | 230 | RA | AF | -0.586 | -0.844 | -0.357 | 0.343 | 0.127 | 0.712 | 0.000 | 0.007 |
| 18 | 57635822 | 58527236 | 1799 | 128 | RA | AF | -0.468 | -1.000 | -0.010 | 0.219 | 0.003 | 1.000 | 0.048 | 0.368 |
| 18 | 62074994 | 63109755 | 2234 | 148 | RA | AF | 0.797 | 0.237 | 1.000 | 0.635 | 0.058 | 1.000 | 0.008 | 0.247 |
| 18 | 72785207 | 73568260 | 1806 | 217 | RA | AF | 0.561 | 0.132 | 1.000 | 0.315 | 0.019 | 1.000 | 0.012 | 0.255 |
| 19 | 2253175 | 3085446 | 1929 | 257 | RA | AF | 0.622 | 0.210 | 1.000 | 0.386 | 0.044 | 1.000 | 0.004 | 0.199 |
| 19 | 5478685 | 6455428 | 1661 | 212 | RA | AF | 0.500 | 0.050 | 1.000 | 0.250 | 0.006 | 1.000 | 0.032 | 0.315 |
| 19 | 7249360 | 8199016 | 2008 | 228 | RA | AF | -0.399 | -0.843 | -0.067 | 0.159 | 0.006 | 0.710 | 0.021 | 0.286 |
| 19 | 10028841 | 11681978 | 2629 | 272 | RA | AF | 0.478 | 0.238 | 0.751 | 0.228 | 0.057 | 0.565 | 0.000 | 0.050 |
| 19 | 33785836 | 34633274 | 1713 | 224 | RA | AF | 0.719 | 0.156 | 1.000 | 0.517 | 0.027 | 1.000 | 0.017 | 0.270 |
| 19 | 48573066 | 49415189 | 1969 | 204 | RA | AF | 0.417 | -0.001 | 0.951 | 0.174 | 0.000 | 0.906 | 0.048 | 0.368 |
| 19 | 50451926 | 51259178 | 1315 | 208 | RA | AF | -0.449 | -0.781 | -0.169 | 0.201 | 0.029 | 0.610 | 0.003 | 0.176 |
| 19 | 54602371 | 55182973 | 1488 | 253 | RA | AF | -0.380 | -0.960 | -0.041 | 0.145 | 0.003 | 0.922 | 0.031 | 0.309 |
| 19 | 55182974 | 55714085 | 1198 | 182 | RA | AF | -0.884 | -1.000 | -0.402 | 0.781 | 0.162 | 1.000 | 0.001 | 0.086 |
| 20 | 60795 | 884241 | 2114 | 314 | RA | AF | 0.475 | 0.164 | 0.851 | 0.226 | 0.027 | 0.724 | 0.004 | 0.199 |
| 20 | 17359291 | 18058524 | 1702 | 203 | RA | AF | -0.698 | -1.000 | -0.038 | 0.487 | 0.009 | 1.000 | 0.038 | 0.337 |
| 20 | 51533751 | 52411532 | 1685 | 254 | RA | AF | 0.385 | 0.016 | 0.817 | 0.148 | 0.002 | 0.668 | 0.046 | 0.359 |
| 20 | 56951774 | 57924037 | 1606 | 244 | RA | AF | 0.311 | 0.055 | 0.594 | 0.097 | 0.004 | 0.353 | 0.022 | 0.286 |
| 21 | 40480832 | 41344742 | 2213 | 188 | RA | AF | 0.786 | 0.128 | 1.000 | 0.619 | 0.024 | 1.000 | 0.024 | 0.287 |
| 1 | 20556203 | 21416121 | 1614 | 153 | RA | CAD | 0.692 | 0.049 | 1.000 | 0.478 | 0.011 | 1.000 | 0.037 | 0.384 |
| 1 | 68633861 | 69438909 | 1562 | 120 | RA | CAD | 0.860 | 0.040 | 1.000 | 0.739 | 0.014 | 1.000 | 0.049 | 0.433 |
| 1 | 87667573 | 88656689 | 1617 | 212 | RA | CAD | -0.613 | -1.000 | -0.093 | 0.376 | 0.012 | 1.000 | 0.023 | 0.296 |
| 1 | 215825792 | 216658126 | 1479 | 168 | RA | CAD | 0.666 | 0.088 | 1.000 | 0.444 | 0.014 | 1.000 | 0.033 | 0.375 |
| 2 | 26894103 | 28819510 | 2446 | 199 | RA | CAD | 0.611 | 0.167 | 1.000 | 0.374 | 0.030 | 1.000 | 0.011 | 0.227 |
| 2 | 71385814 | 72160302 | 1531 | 177 | RA | CAD | 0.887 | 0.267 | 1.000 | 0.786 | 0.072 | 1.000 | 0.007 | 0.221 |
| 2 | 103305258 | 105120578 | 2455 | 181 | RA | CAD | 0.714 | 0.079 | 1.000 | 0.510 | 0.013 | 1.000 | 0.035 | 0.380 |
| 2 | 203639687 | 205038119 | 1856 | 130 | RA | CAD | -0.302 | -0.599 | -0.025 | 0.091 | 0.002 | 0.359 | 0.037 | 0.384 |
| 3 | 59998584 | 60571139 | 1785 | 213 | RA | CAD | 0.782 | 0.103 | 1.000 | 0.612 | 0.022 | 1.000 | 0.029 | 0.346 |
| 3 | 123518508 | 124823006 | 2609 | 197 | RA | CAD | 0.399 | 0.026 | 0.861 | 0.159 | 0.003 | 0.741 | 0.041 | 0.392 |
| 3 | 171134850 | 172004760 | 1717 | 165 | RA | CAD | 0.704 | 0.235 | 1.000 | 0.496 | 0.055 | 1.000 | 0.006 | 0.216 |
| 3 | 196383153 | 197147990 | 1476 | 189 | RA | CAD | 0.434 | 0.019 | 1.000 | 0.188 | 0.004 | 1.000 | 0.039 | 0.384 |
| 4 | 7726035 | 8309826 | 1704 | 229 | RA | CAD | 0.821 | 0.431 | 1.000 | 0.675 | 0.185 | 1.000 | 0.000 | 0.061 |
| 4 | 32260118 | 32746820 | 1653 | 71 | RA | CAD | 0.775 | 0.165 | 1.000 | 0.600 | 0.035 | 1.000 | 0.021 | 0.292 |
| 4 | 61971162 | 63337954 | 2237 | 181 | RA | CAD | -0.776 | -1.000 | -0.202 | 0.602 | 0.042 | 1.000 | 0.014 | 0.231 |
| 4 | 151014323 | 152162098 | 1457 | 89 | RA | CAD | 0.606 | 0.032 | 1.000 | 0.368 | 0.007 | 1.000 | 0.046 | 0.421 |
| 5 | 124109486 | 125131720 | 2253 | 227 | RA | CAD | 0.872 | 0.209 | 1.000 | 0.760 | 0.048 | 1.000 | 0.013 | 0.231 |
| 6 | 1380680 | 2746531 | 2987 | 286 | RA | CAD | 0.800 | 0.164 | 1.000 | 0.640 | 0.030 | 1.000 | 0.019 | 0.292 |
| 6 | 128763381 | 129850178 | 1839 | 145 | RA | CAD | -0.733 | -1.000 | -0.152 | 0.538 | 0.027 | 1.000 | 0.021 | 0.292 |
| 6 | 132890695 | 134313643 | 2797 | 208 | RA | CAD | -0.499 | -0.947 | -0.136 | 0.249 | 0.018 | 0.897 | 0.008 | 0.221 |
| 6 | 160583919 | 161371014 | 1755 | 121 | RA | CAD | -0.315 | -0.588 | -0.070 | 0.099 | 0.005 | 0.346 | 0.014 | 0.231 |
| 7 | 99465541 | 100849307 | 1544 | 161 | RA | CAD | -1.000 | -1.000 | -0.387 | 1.000 | 0.150 | 1.000 | 0.002 | 0.151 |
| 7 | 137657460 | 138755107 | 2228 | 267 | RA | CAD | 0.652 | 0.231 | 1.000 | 0.425 | 0.054 | 1.000 | 0.004 | 0.194 |
| 8 | 125453323 | 126766827 | 2520 | 243 | RA | CAD | 0.378 | 0.033 | 0.773 | 0.143 | 0.003 | 0.598 | 0.034 | 0.375 |
| 9 | 21674033 | 23091193 | 2657 | 236 | RA | CAD | -0.301 | -0.526 | -0.087 | 0.090 | 0.008 | 0.277 | 0.006 | 0.216 |
| 9 | 140097760 | 141146682 | 774 | 114 | RA | CAD | -0.594 | -1.000 | -0.156 | 0.353 | 0.025 | 1.000 | 0.010 | 0.224 |
| 10 | 3225337 | 3880322 | 1651 | 201 | RA | CAD | -0.611 | -1.000 | -0.106 | 0.373 | 0.014 | 1.000 | 0.023 | 0.296 |
| 10 | 7659873 | 8771920 | 2779 | 273 | RA | CAD | -0.561 | -1.000 | -0.025 | 0.315 | 0.005 | 1.000 | 0.044 | 0.408 |
| 10 | 85463485 | 86459980 | 2303 | 166 | RA | CAD | 0.838 | 0.137 | 1.000 | 0.702 | 0.026 | 1.000 | 0.026 | 0.335 |
| 10 | 91021322 | 91960435 | 2145 | 158 | RA | CAD | 0.520 | 0.151 | 0.989 | 0.270 | 0.023 | 0.977 | 0.009 | 0.221 |
| 10 | 123856185 | 124894142 | 2235 | 211 | RA | CAD | -0.367 | -0.727 | -0.052 | 0.135 | 0.004 | 0.528 | 0.028 | 0.344 |
| 11 | 11632651 | 12470449 | 2078 | 267 | RA | CAD | 0.476 | 0.077 | 1.000 | 0.227 | 0.007 | 1.000 | 0.021 | 0.292 |
| 11 | 13364728 | 15126767 | 2645 | 174 | RA | CAD | 1.000 | 0.422 | 1.000 | 1.000 | 0.178 | 1.000 | 0.001 | 0.121 |
| 11 | 51098367 | 51581595 | 1085 | 7 | RA | CAD | -1.000 | -1.000 | -0.416 | 1.000 | 0.174 | 1.000 | 0.010 | 0.224 |
| 11 | 51581596 | 55477633 | 1644 | 16 | RA | CAD | -1.000 | -1.000 | -0.502 | 1.000 | 0.252 | 1.000 | 0.003 | 0.189 |
| 11 | 60515106 | 61717117 | 1539 | 214 | RA | CAD | 0.538 | 0.144 | 1.000 | 0.290 | 0.021 | 1.000 | 0.010 | 0.224 |
| 12 | 1942427 | 3069506 | 2074 | 253 | RA | CAD | -0.487 | -1.000 | -0.089 | 0.237 | 0.010 | 1.000 | 0.021 | 0.292 |
| 12 | 64403858 | 66114643 | 2109 | 197 | RA | CAD | 0.568 | 0.121 | 1.000 | 0.323 | 0.016 | 1.000 | 0.017 | 0.268 |
| 12 | 96828315 | 97814021 | 2108 | 165 | RA | CAD | 1.000 | 0.310 | 1.000 | 1.000 | 0.101 | 1.000 | 0.007 | 0.216 |
| 13 | 73114510 | 74437635 | 2775 | 232 | RA | CAD | 0.810 | 0.312 | 1.000 | 0.656 | 0.098 | 1.000 | 0.004 | 0.194 |
| 13 | 74437636 | 75882384 | 2862 | 239 | RA | CAD | 0.546 | 0.039 | 1.000 | 0.298 | 0.006 | 1.000 | 0.038 | 0.384 |
| 14 | 22760701 | 23985936 | 2434 | 286 | RA | CAD | -0.682 | -1.000 | -0.318 | 0.465 | 0.101 | 1.000 | 0.001 | 0.121 |
| 14 | 93386329 | 94892240 | 3038 | 268 | RA | CAD | 1.000 | 0.317 | 1.000 | 1.000 | 0.102 | 1.000 | 0.001 | 0.121 |
| 14 | 97174315 | 98268391 | 1987 | 218 | RA | CAD | 0.523 | 0.125 | 1.000 | 0.274 | 0.017 | 1.000 | 0.014 | 0.231 |
| 16 | 6446082 | 7051429 | 2242 | 237 | RA | CAD | 0.513 | 0.047 | 1.000 | 0.263 | 0.007 | 1.000 | 0.032 | 0.372 |
| 16 | 25235252 | 26154122 | 2194 | 206 | RA | CAD | -0.464 | -1.000 | -0.011 | 0.215 | 0.003 | 1.000 | 0.043 | 0.406 |
| 16 | 81437862 | 82310204 | 2456 | 290 | RA | CAD | 0.544 | 0.129 | 1.000 | 0.296 | 0.017 | 1.000 | 0.013 | 0.231 |
| 16 | 85146806 | 86058597 | 1995 | 316 | RA | CAD | -0.420 | -0.946 | -0.096 | 0.176 | 0.009 | 0.894 | 0.013 | 0.231 |
| 17 | 78438333 | 79333774 | 2448 | 211 | RA | CAD | 0.739 | 0.071 | 1.000 | 0.546 | 0.015 | 1.000 | 0.038 | 0.384 |
| 18 | 20009697 | 21622716 | 2321 | 166 | RA | CAD | 0.562 | 0.159 | 1.000 | 0.315 | 0.026 | 1.000 | 0.008 | 0.221 |
| 19 | 4741719 | 5478684 | 1675 | 244 | RA | CAD | -0.718 | -1.000 | -0.247 | 0.516 | 0.062 | 1.000 | 0.005 | 0.216 |
| 19 | 51259179 | 51903804 | 1543 | 245 | RA | CAD | -0.656 | -1.000 | -0.222 | 0.431 | 0.050 | 1.000 | 0.004 | 0.210 |
| 20 | 56951774 | 57924037 | 1593 | 241 | RA | CAD | 0.531 | 0.161 | 1.000 | 0.282 | 0.027 | 1.000 | 0.006 | 0.216 |
| 21 | 34383795 | 35729261 | 2264 | 255 | RA | CAD | -0.474 | -0.825 | -0.174 | 0.225 | 0.030 | 0.681 | 0.003 | 0.189 |
| 1 | 3582048 | 4281476 | 1595 | 177 | RA | HF | 0.429 | 0.038 | 0.966 | 0.184 | 0.004 | 0.933 | 0.036 | 0.350 |
| 1 | 5362405 | 6136814 | 1646 | 211 | RA | HF | -0.537 | -1.000 | -0.091 | 0.288 | 0.010 | 1.000 | 0.020 | 0.297 |
| 1 | 7711795 | 8580987 | 1660 | 171 | RA | HF | 0.594 | 0.305 | 0.939 | 0.353 | 0.093 | 0.881 | 0.000 | 0.076 |
| 1 | 10753428 | 11709173 | 1654 | 192 | RA | HF | -0.651 | -1.000 | -0.208 | 0.424 | 0.044 | 1.000 | 0.007 | 0.212 |
| 1 | 17557747 | 18427820 | 1884 | 197 | RA | HF | -0.346 | -0.668 | -0.055 | 0.120 | 0.004 | 0.446 | 0.022 | 0.302 |
| 1 | 22145630 | 22740287 | 1522 | 156 | RA | HF | 0.383 | 0.021 | 0.783 | 0.147 | 0.003 | 0.614 | 0.039 | 0.355 |
| 1 | 35201627 | 36855139 | 1310 | 136 | RA | HF | 0.400 | 0.059 | 0.834 | 0.160 | 0.006 | 0.696 | 0.025 | 0.320 |
| 1 | 38474037 | 40200950 | 2722 | 255 | RA | HF | -0.579 | -1.000 | -0.172 | 0.336 | 0.030 | 1.000 | 0.007 | 0.220 |
| 1 | 53114187 | 54771293 | 3029 | 246 | RA | HF | 0.554 | 0.089 | 1.000 | 0.307 | 0.010 | 1.000 | 0.024 | 0.316 |
| 1 | 83461216 | 84728347 | 1996 | 142 | RA | HF | 0.308 | 0.036 | 0.584 | 0.095 | 0.002 | 0.341 | 0.027 | 0.330 |
| 1 | 101188316 | 102424611 | 2469 | 191 | RA | HF | -0.594 | -1.000 | -0.044 | 0.353 | 0.006 | 1.000 | 0.034 | 0.350 |
| 1 | 110224231 | 111134062 | 1552 | 229 | RA | HF | 0.388 | 0.115 | 0.681 | 0.151 | 0.013 | 0.464 | 0.006 | 0.210 |
| 1 | 112489249 | 113418037 | 1556 | 164 | RA | HF | 0.694 | 0.376 | 1.000 | 0.482 | 0.142 | 1.000 | 0.000 | 0.067 |
| 1 | 161945443 | 163141862 | 2452 | 227 | RA | HF | -0.546 | -1.000 | -0.146 | 0.298 | 0.022 | 1.000 | 0.010 | 0.240 |
| 1 | 197935822 | 199263389 | 2292 | 171 | RA | HF | -0.689 | -1.000 | -0.232 | 0.475 | 0.055 | 1.000 | 0.006 | 0.202 |
| 1 | 208162952 | 209711169 | 2749 | 289 | RA | HF | -0.505 | -0.955 | -0.160 | 0.255 | 0.026 | 0.912 | 0.005 | 0.202 |
| 1 | 218563961 | 220073132 | 2896 | 176 | RA | HF | -0.844 | -1.000 | -0.365 | 0.713 | 0.133 | 1.000 | 0.001 | 0.104 |
| 1 | 233639474 | 234365796 | 1825 | 178 | RA | HF | 0.777 | 0.375 | 1.000 | 0.604 | 0.140 | 1.000 | 0.000 | 0.088 |
| 2 | 4016105 | 4766969 | 1324 | 161 | RA | HF | 0.728 | 0.149 | 1.000 | 0.529 | 0.025 | 1.000 | 0.016 | 0.285 |
| 2 | 29627933 | 30575619 | 1887 | 190 | RA | HF | -0.556 | -1.000 | -0.038 | 0.309 | 0.006 | 1.000 | 0.038 | 0.355 |
| 2 | 48171059 | 49114132 | 1663 | 166 | RA | HF | -0.612 | -1.000 | -0.090 | 0.374 | 0.011 | 1.000 | 0.026 | 0.330 |
| 2 | 68877775 | 70756283 | 3061 | 231 | RA | HF | -0.496 | -0.928 | -0.142 | 0.246 | 0.020 | 0.860 | 0.008 | 0.234 |
| 2 | 73544291 | 75148103 | 1940 | 171 | RA | HF | 1.000 | 0.201 | 1.000 | 1.000 | 0.046 | 1.000 | 0.012 | 0.267 |
| 2 | 207726595 | 208674588 | 1573 | 171 | RA | HF | 0.630 | 0.151 | 1.000 | 0.396 | 0.024 | 1.000 | 0.013 | 0.275 |
| 2 | 221445192 | 222327713 | 1582 | 172 | RA | HF | -0.435 | -0.975 | -0.001 | 0.189 | 0.003 | 0.950 | 0.048 | 0.383 |
| 2 | 227557842 | 228812485 | 2950 | 223 | RA | HF | -0.510 | -1.000 | -0.045 | 0.260 | 0.006 | 1.000 | 0.035 | 0.350 |
| 3 | 11226368 | 11997658 | 1543 | 186 | RA | HF | 0.647 | 0.313 | 1.000 | 0.419 | 0.098 | 1.000 | 0.000 | 0.088 |
| 3 | 17182495 | 19030988 | 2778 | 158 | RA | HF | 0.766 | 0.079 | 1.000 | 0.587 | 0.018 | 1.000 | 0.035 | 0.350 |
| 3 | 19030989 | 20754842 | 2853 | 182 | RA | HF | 0.580 | 0.052 | 1.000 | 0.337 | 0.008 | 1.000 | 0.038 | 0.354 |
| 3 | 20754843 | 21790867 | 2623 | 221 | RA | HF | -0.462 | -0.969 | -0.076 | 0.214 | 0.007 | 0.939 | 0.022 | 0.302 |
| 3 | 25516194 | 26905340 | 2406 | 170 | RA | HF | 0.706 | 0.159 | 1.000 | 0.498 | 0.029 | 1.000 | 0.015 | 0.285 |
| 3 | 35913034 | 36840136 | 1839 | 162 | RA | HF | -0.649 | -1.000 | -0.086 | 0.421 | 0.012 | 1.000 | 0.027 | 0.330 |
| 3 | 36840137 | 38729767 | 2613 | 231 | RA | HF | 0.616 | 0.018 | 1.000 | 0.380 | 0.007 | 1.000 | 0.043 | 0.355 |
| 3 | 56954879 | 58557718 | 2845 | 162 | RA | HF | -0.879 | -1.000 | -0.390 | 0.773 | 0.152 | 1.000 | 0.001 | 0.090 |
| 3 | 65326752 | 66702084 | 2023 | 248 | RA | HF | 0.596 | 0.315 | 0.922 | 0.355 | 0.099 | 0.849 | 0.000 | 0.067 |
| 3 | 68319771 | 69784969 | 2981 | 223 | RA | HF | 0.381 | 0.029 | 0.784 | 0.145 | 0.003 | 0.615 | 0.038 | 0.355 |
| 3 | 69784970 | 71223281 | 1991 | 202 | RA | HF | -0.340 | -0.628 | -0.069 | 0.115 | 0.005 | 0.394 | 0.015 | 0.285 |
| 3 | 113657666 | 115649909 | 2529 | 256 | RA | HF | 0.348 | 0.028 | 0.712 | 0.121 | 0.003 | 0.507 | 0.035 | 0.350 |
| 3 | 130242361 | 131373470 | 1529 | 109 | RA | HF | -0.782 | -1.000 | -0.048 | 0.612 | 0.012 | 1.000 | 0.042 | 0.355 |
| 3 | 145903839 | 147358243 | 2726 | 182 | RA | HF | 0.723 | 0.060 | 1.000 | 0.522 | 0.011 | 1.000 | 0.040 | 0.355 |
| 3 | 148638309 | 149998411 | 2572 | 250 | RA | HF | -0.569 | -1.000 | -0.043 | 0.324 | 0.007 | 1.000 | 0.034 | 0.350 |
| 3 | 149998412 | 151131307 | 1843 | 199 | RA | HF | 0.702 | 0.178 | 1.000 | 0.493 | 0.033 | 1.000 | 0.011 | 0.249 |
| 3 | 176931164 | 178110322 | 1865 | 208 | RA | HF | 0.620 | 0.195 | 1.000 | 0.384 | 0.038 | 1.000 | 0.007 | 0.212 |
| 4 | 812416 | 1529267 | 1506 | 131 | RA | HF | 0.507 | 0.194 | 0.868 | 0.257 | 0.038 | 0.754 | 0.002 | 0.129 |
| 4 | 15910802 | 17290236 | 2835 | 261 | RA | HF | -0.504 | -1.000 | -0.090 | 0.254 | 0.010 | 1.000 | 0.018 | 0.294 |
| 4 | 21416381 | 22835185 | 3036 | 174 | RA | HF | 0.561 | 0.020 | 1.000 | 0.315 | 0.005 | 1.000 | 0.050 | 0.383 |
| 4 | 24764725 | 25832018 | 2414 | 241 | RA | HF | 0.995 | 0.450 | 1.000 | 0.991 | 0.203 | 1.000 | 0.001 | 0.090 |
| 4 | 37880861 | 38984838 | 2623 | 252 | RA | HF | -0.444 | -0.794 | -0.142 | 0.197 | 0.020 | 0.630 | 0.005 | 0.202 |
| 4 | 43962562 | 45186767 | 2618 | 130 | RA | HF | -0.820 | -1.000 | -0.208 | 0.673 | 0.047 | 1.000 | 0.017 | 0.285 |
| 4 | 57103101 | 58011085 | 2218 | 194 | RA | HF | 0.533 | 0.019 | 1.000 | 0.284 | 0.005 | 1.000 | 0.047 | 0.375 |
| 4 | 69603369 | 70516836 | 2434 | 59 | RA | HF | -0.409 | -0.887 | -0.014 | 0.167 | 0.003 | 0.787 | 0.047 | 0.377 |
| 4 | 84003063 | 85437805 | 2425 | 186 | RA | HF | -0.443 | -0.969 | -0.051 | 0.196 | 0.005 | 0.940 | 0.034 | 0.350 |
| 4 | 109978983 | 111733579 | 2874 | 227 | RA | HF | -0.434 | -0.796 | -0.140 | 0.188 | 0.020 | 0.633 | 0.006 | 0.203 |
| 4 | 127279172 | 128321876 | 1820 | 125 | RA | HF | 0.955 | 0.390 | 1.000 | 0.911 | 0.154 | 1.000 | 0.002 | 0.131 |
| 4 | 168623791 | 169555114 | 1899 | 148 | RA | HF | 0.709 | 0.160 | 1.000 | 0.503 | 0.029 | 1.000 | 0.019 | 0.294 |
| 4 | 182065647 | 182946258 | 2473 | 261 | RA | HF | -0.432 | -0.852 | -0.097 | 0.187 | 0.010 | 0.726 | 0.011 | 0.252 |
| 4 | 185483263 | 186232882 | 1946 | 199 | RA | HF | 0.535 | 0.176 | 1.000 | 0.286 | 0.031 | 1.000 | 0.005 | 0.202 |
| 5 | 32727915 | 33971056 | 2557 | 185 | RA | HF | 0.574 | 0.042 | 1.000 | 0.330 | 0.007 | 1.000 | 0.040 | 0.355 |
| 5 | 33971057 | 35157146 | 2063 | 177 | RA | HF | 0.423 | 0.048 | 0.863 | 0.179 | 0.004 | 0.745 | 0.027 | 0.330 |
| 5 | 65606871 | 67096191 | 2660 | 207 | RA | HF | 0.575 | 0.084 | 1.000 | 0.330 | 0.012 | 1.000 | 0.025 | 0.320 |
| 5 | 102903986 | 103788460 | 1531 | 171 | RA | HF | 1.000 | 0.427 | 1.000 | 1.000 | 0.183 | 1.000 | 0.001 | 0.090 |
| 5 | 117348498 | 118605251 | 2331 | 181 | RA | HF | 0.357 | 0.008 | 0.763 | 0.127 | 0.002 | 0.583 | 0.050 | 0.383 |
| 5 | 135344944 | 136949853 | 2708 | 192 | RA | HF | -0.675 | -1.000 | -0.063 | 0.456 | 0.009 | 1.000 | 0.033 | 0.350 |
| 5 | 141253831 | 142532956 | 2471 | 250 | RA | HF | -0.400 | -0.779 | -0.075 | 0.160 | 0.007 | 0.607 | 0.019 | 0.294 |
| 5 | 147180172 | 148662214 | 2700 | 193 | RA | HF | -0.688 | -1.000 | -0.228 | 0.473 | 0.052 | 1.000 | 0.005 | 0.202 |
| 5 | 161478178 | 162386027 | 1423 | 127 | RA | HF | 0.432 | 0.020 | 0.947 | 0.187 | 0.003 | 0.896 | 0.043 | 0.355 |
| 5 | 166863414 | 168342743 | 2569 | 287 | RA | HF | -0.688 | -1.000 | -0.308 | 0.473 | 0.095 | 1.000 | 0.001 | 0.090 |
| 6 | 3964073 | 4657086 | 1793 | 152 | RA | HF | 0.665 | 0.074 | 1.000 | 0.442 | 0.011 | 1.000 | 0.035 | 0.350 |
| 6 | 10416551 | 11790671 | 2714 | 252 | RA | HF | -0.337 | -0.660 | -0.045 | 0.113 | 0.003 | 0.435 | 0.028 | 0.330 |
| 6 | 13352327 | 15069421 | 2933 | 273 | RA | HF | 0.432 | 0.031 | 0.953 | 0.187 | 0.003 | 0.907 | 0.037 | 0.354 |
| 6 | 23939307 | 24950379 | 2440 | 217 | RA | HF | -0.560 | -1.000 | -0.035 | 0.314 | 0.007 | 1.000 | 0.040 | 0.355 |
| 6 | 31250557 | 31320268 | 1304 | 53 | RA | HF | -0.133 | -0.245 | -0.018 | 0.018 | 0.000 | 0.060 | 0.023 | 0.308 |
| 6 | 38688721 | 39785907 | 2558 | 211 | RA | HF | -0.608 | -1.000 | -0.110 | 0.369 | 0.015 | 1.000 | 0.021 | 0.299 |
| 6 | 160583919 | 161371014 | 1736 | 119 | RA | HF | -0.506 | -0.921 | -0.157 | 0.256 | 0.025 | 0.848 | 0.007 | 0.210 |
| 6 | 168707339 | 169667886 | 2969 | 214 | RA | HF | -0.503 | -1.000 | -0.097 | 0.253 | 0.012 | 1.000 | 0.019 | 0.294 |
| 7 | 17596579 | 19131026 | 2627 | 276 | RA | HF | -0.574 | -1.000 | -0.129 | 0.330 | 0.018 | 1.000 | 0.013 | 0.280 |
| 7 | 24894120 | 25671575 | 1791 | 211 | RA | HF | -0.738 | -1.000 | -0.242 | 0.545 | 0.059 | 1.000 | 0.007 | 0.210 |
| 7 | 36507691 | 37981936 | 2981 | 256 | RA | HF | 0.683 | 0.262 | 1.000 | 0.467 | 0.069 | 1.000 | 0.002 | 0.131 |
| 7 | 55161395 | 56303513 | 2410 | 178 | RA | HF | -0.428 | -1.000 | -0.011 | 0.183 | 0.003 | 1.000 | 0.044 | 0.357 |
| 7 | 81533567 | 82759283 | 2382 | 196 | RA | HF | 0.412 | 0.113 | 0.759 | 0.170 | 0.013 | 0.576 | 0.010 | 0.249 |
| 7 | 137657460 | 138755107 | 2217 | 266 | RA | HF | -0.481 | -1.000 | -0.024 | 0.231 | 0.004 | 1.000 | 0.043 | 0.355 |
| 7 | 138755108 | 140217630 | 2329 | 246 | RA | HF | -0.327 | -0.629 | -0.053 | 0.107 | 0.003 | 0.395 | 0.022 | 0.306 |
| 7 | 147352172 | 148622913 | 2682 | 242 | RA | HF | -0.414 | -0.894 | -0.035 | 0.171 | 0.004 | 0.799 | 0.035 | 0.350 |
| 7 | 157199332 | 158031247 | 2199 | 228 | RA | HF | -0.362 | -0.752 | -0.015 | 0.131 | 0.002 | 0.566 | 0.041 | 0.355 |
| 8 | 6019164 | 6663427 | 2482 | 262 | RA | HF | -0.383 | -0.723 | -0.083 | 0.147 | 0.007 | 0.522 | 0.015 | 0.285 |
| 8 | 8589771 | 9167795 | 1597 | 199 | RA | HF | 0.384 | 0.087 | 0.705 | 0.147 | 0.008 | 0.497 | 0.014 | 0.283 |
| 8 | 19488889 | 20135628 | 1648 | 200 | RA | HF | 0.418 | 0.040 | 0.876 | 0.175 | 0.004 | 0.767 | 0.031 | 0.350 |
| 8 | 21650454 | 22895018 | 2061 | 259 | RA | HF | 0.558 | 0.098 | 1.000 | 0.312 | 0.012 | 1.000 | 0.020 | 0.297 |
| 8 | 66960439 | 68683823 | 1751 | 156 | RA | HF | -0.670 | -1.000 | -0.249 | 0.449 | 0.062 | 1.000 | 0.003 | 0.164 |
| 8 | 70004666 | 70872088 | 1635 | 177 | RA | HF | 0.335 | 0.028 | 0.659 | 0.112 | 0.002 | 0.435 | 0.029 | 0.337 |
| 8 | 142611890 | 143611461 | 2100 | 210 | RA | HF | 0.335 | 0.064 | 0.612 | 0.112 | 0.005 | 0.374 | 0.016 | 0.285 |
| 9 | 3079046 | 4025355 | 1804 | 246 | RA | HF | 0.746 | 0.026 | 1.000 | 0.556 | 0.010 | 1.000 | 0.042 | 0.355 |
| 9 | 9920259 | 10675474 | 2185 | 204 | RA | HF | -0.554 | -1.000 | -0.111 | 0.306 | 0.014 | 1.000 | 0.014 | 0.285 |
| 9 | 94175375 | 94924482 | 1692 | 124 | RA | HF | -0.434 | -0.974 | -0.008 | 0.188 | 0.003 | 0.948 | 0.049 | 0.383 |
| 9 | 97418634 | 98885862 | 2239 | 188 | RA | HF | 0.594 | 0.036 | 1.000 | 0.353 | 0.007 | 1.000 | 0.043 | 0.356 |
| 9 | 132999453 | 134141936 | 1936 | 268 | RA | HF | 0.462 | 0.014 | 1.000 | 0.213 | 0.004 | 1.000 | 0.042 | 0.355 |
| 9 | 136042491 | 136770926 | 1929 | 219 | RA | HF | -0.300 | -0.604 | -0.014 | 0.090 | 0.001 | 0.365 | 0.040 | 0.355 |
| 10 | 3225337 | 3880322 | 1644 | 200 | RA | HF | 0.714 | 0.222 | 1.000 | 0.509 | 0.050 | 1.000 | 0.008 | 0.221 |
| 10 | 6234719 | 6897037 | 1637 | 240 | RA | HF | -0.254 | -0.498 | -0.014 | 0.065 | 0.001 | 0.248 | 0.037 | 0.354 |
| 10 | 10081280 | 10969481 | 1967 | 196 | RA | HF | -0.683 | -1.000 | -0.213 | 0.467 | 0.046 | 1.000 | 0.006 | 0.202 |
| 10 | 13208062 | 14008135 | 2014 | 264 | RA | HF | -0.535 | -1.000 | -0.080 | 0.286 | 0.009 | 1.000 | 0.022 | 0.302 |
| 10 | 17065861 | 18529742 | 2681 | 231 | RA | HF | 0.379 | 0.032 | 0.771 | 0.144 | 0.003 | 0.594 | 0.033 | 0.350 |
| 10 | 61470384 | 62626465 | 2517 | 174 | RA | HF | -0.746 | -1.000 | -0.066 | 0.557 | 0.014 | 1.000 | 0.033 | 0.350 |
| 10 | 88443653 | 89971628 | 1896 | 178 | RA | HF | -0.499 | -0.848 | -0.198 | 0.249 | 0.039 | 0.720 | 0.002 | 0.126 |
| 10 | 120592840 | 121874445 | 2652 | 216 | RA | HF | 0.493 | 0.009 | 1.000 | 0.243 | 0.004 | 1.000 | 0.049 | 0.383 |
| 10 | 123856185 | 124894142 | 2196 | 206 | RA | HF | -0.375 | -0.766 | -0.029 | 0.141 | 0.002 | 0.587 | 0.034 | 0.350 |
| 10 | 129134739 | 129831969 | 2268 | 237 | RA | HF | 0.441 | 0.018 | 1.000 | 0.194 | 0.003 | 1.000 | 0.044 | 0.357 |
| 10 | 133818037 | 134856054 | 2425 | 255 | RA | HF | -0.601 | -1.000 | -0.054 | 0.361 | 0.008 | 1.000 | 0.034 | 0.350 |
| 11 | 87150 | 1061399 | 1937 | 221 | RA | HF | 0.627 | 0.274 | 1.000 | 0.393 | 0.075 | 1.000 | 0.001 | 0.090 |
| 11 | 1857846 | 2477449 | 1459 | 188 | RA | HF | 0.462 | 0.064 | 1.000 | 0.213 | 0.006 | 1.000 | 0.029 | 0.336 |
| 11 | 3362509 | 4405707 | 1638 | 141 | RA | HF | -0.460 | -0.915 | -0.086 | 0.211 | 0.009 | 0.837 | 0.019 | 0.294 |
| 11 | 11632651 | 12470449 | 2062 | 263 | RA | HF | 0.245 | 0.004 | 0.496 | 0.060 | 0.001 | 0.247 | 0.049 | 0.383 |
| 11 | 16383387 | 17583948 | 2067 | 166 | RA | HF | -0.744 | -1.000 | -0.218 | 0.554 | 0.048 | 1.000 | 0.009 | 0.240 |
| 11 | 35500368 | 36331189 | 1581 | 208 | RA | HF | -0.348 | -0.678 | -0.050 | 0.121 | 0.004 | 0.460 | 0.024 | 0.310 |
| 11 | 132584402 | 133521642 | 1916 | 277 | RA | HF | -0.741 | -1.000 | -0.303 | 0.550 | 0.092 | 1.000 | 0.001 | 0.090 |
| 11 | 133521643 | 134351064 | 1568 | 236 | RA | HF | -0.446 | -0.938 | -0.097 | 0.199 | 0.010 | 0.879 | 0.016 | 0.285 |
| 12 | 1942427 | 3069506 | 2047 | 250 | RA | HF | 0.372 | 0.103 | 0.674 | 0.138 | 0.011 | 0.455 | 0.010 | 0.240 |
| 12 | 74080748 | 75563968 | 2481 | 117 | RA | HF | 0.558 | 0.101 | 1.000 | 0.311 | 0.013 | 1.000 | 0.020 | 0.297 |
| 12 | 75563969 | 76524573 | 1669 | 189 | RA | HF | 0.515 | 0.052 | 1.000 | 0.265 | 0.006 | 1.000 | 0.033 | 0.350 |
| 12 | 108183189 | 109031819 | 1963 | 168 | RA | HF | -0.526 | -1.000 | -0.100 | 0.277 | 0.012 | 1.000 | 0.019 | 0.294 |
| 12 | 111592382 | 113947983 | 2508 | 193 | RA | HF | 0.425 | 0.094 | 0.839 | 0.180 | 0.009 | 0.704 | 0.016 | 0.285 |
| 12 | 114685671 | 115439451 | 1610 | 206 | RA | HF | 0.512 | 0.219 | 0.845 | 0.262 | 0.048 | 0.714 | 0.001 | 0.090 |
| 13 | 21540734 | 22795188 | 2745 | 289 | RA | HF | 0.337 | 0.022 | 0.681 | 0.113 | 0.002 | 0.463 | 0.037 | 0.352 |
| 13 | 22795189 | 23729049 | 2593 | 257 | RA | HF | 0.429 | 0.140 | 0.759 | 0.184 | 0.019 | 0.577 | 0.005 | 0.202 |
| 13 | 27392006 | 28587125 | 2724 | 261 | RA | HF | -0.899 | -1.000 | -0.438 | 0.809 | 0.192 | 1.000 | 0.000 | 0.073 |
| 13 | 36579414 | 37499810 | 1657 | 147 | RA | HF | 0.942 | 0.203 | 1.000 | 0.887 | 0.048 | 1.000 | 0.018 | 0.294 |
| 13 | 46493237 | 47433528 | 1708 | 167 | RA | HF | 0.544 | 0.136 | 1.000 | 0.296 | 0.019 | 1.000 | 0.014 | 0.280 |
| 13 | 74437636 | 75882384 | 2756 | 236 | RA | HF | 0.582 | 0.160 | 1.000 | 0.338 | 0.026 | 1.000 | 0.010 | 0.240 |
| 13 | 94925137 | 96132647 | 3056 | 271 | RA | HF | 0.517 | 0.008 | 1.000 | 0.268 | 0.005 | 1.000 | 0.046 | 0.374 |
| 13 | 99402295 | 100470868 | 2245 | 161 | RA | HF | 0.482 | 0.027 | 1.000 | 0.232 | 0.004 | 1.000 | 0.043 | 0.355 |
| 13 | 100470869 | 101573736 | 1869 | 196 | RA | HF | -0.428 | -0.880 | -0.043 | 0.184 | 0.004 | 0.775 | 0.029 | 0.336 |
| 13 | 109156766 | 109813576 | 1628 | 167 | RA | HF | -0.500 | -1.000 | -0.117 | 0.250 | 0.015 | 1.000 | 0.016 | 0.285 |
| 13 | 109813577 | 110995432 | 2749 | 292 | RA | HF | -0.396 | -0.658 | -0.150 | 0.157 | 0.023 | 0.433 | 0.002 | 0.126 |
| 14 | 22760701 | 23985936 | 2413 | 283 | RA | HF | -0.390 | -0.752 | -0.077 | 0.152 | 0.007 | 0.566 | 0.016 | 0.285 |
| 14 | 29029225 | 30831154 | 2376 | 242 | RA | HF | 0.530 | 0.232 | 0.890 | 0.281 | 0.054 | 0.793 | 0.001 | 0.090 |
| 14 | 32382246 | 33591113 | 2395 | 275 | RA | HF | 0.316 | 0.016 | 0.639 | 0.100 | 0.002 | 0.408 | 0.040 | 0.355 |
| 14 | 57460782 | 58447798 | 1765 | 153 | RA | HF | 0.583 | 0.187 | 1.000 | 0.340 | 0.035 | 1.000 | 0.006 | 0.202 |
| 14 | 61652817 | 62717985 | 1813 | 209 | RA | HF | 0.911 | 0.466 | 1.000 | 0.831 | 0.217 | 1.000 | 0.000 | 0.073 |
| 14 | 64033238 | 65229391 | 1855 | 123 | RA | HF | -0.746 | -1.000 | -0.069 | 0.557 | 0.012 | 1.000 | 0.039 | 0.355 |
| 14 | 77425454 | 78374053 | 2276 | 184 | RA | HF | -0.401 | -0.866 | -0.021 | 0.161 | 0.003 | 0.751 | 0.042 | 0.355 |
| 14 | 93386329 | 94892240 | 3037 | 267 | RA | HF | -1.000 | -1.000 | -0.357 | 1.000 | 0.127 | 1.000 | 0.003 | 0.131 |
| 15 | 22799927 | 24099946 | 1411 | 215 | RA | HF | 0.336 | 0.059 | 0.630 | 0.113 | 0.004 | 0.397 | 0.018 | 0.294 |
| 15 | 26392948 | 27563172 | 2240 | 252 | RA | HF | 0.382 | 0.043 | 0.758 | 0.146 | 0.004 | 0.574 | 0.028 | 0.330 |
| 15 | 35118928 | 36583327 | 2377 | 214 | RA | HF | 0.524 | 0.043 | 1.000 | 0.275 | 0.006 | 1.000 | 0.034 | 0.350 |
| 15 | 59901117 | 61130595 | 2185 | 232 | RA | HF | -0.737 | -1.000 | -0.263 | 0.543 | 0.069 | 1.000 | 0.004 | 0.175 |
| 15 | 69089816 | 70767983 | 2723 | 281 | RA | HF | 0.537 | 0.155 | 1.000 | 0.288 | 0.025 | 1.000 | 0.008 | 0.229 |
| 15 | 86341599 | 87158587 | 1931 | 181 | RA | HF | 0.505 | 0.047 | 1.000 | 0.255 | 0.006 | 1.000 | 0.036 | 0.350 |
| 15 | 100742118 | 101550750 | 1968 | 222 | RA | HF | 0.671 | 0.109 | 1.000 | 0.451 | 0.015 | 1.000 | 0.025 | 0.320 |
| 15 | 101550751 | 102520975 | 1933 | 264 | RA | HF | -0.453 | -1.000 | -0.042 | 0.206 | 0.005 | 1.000 | 0.033 | 0.350 |
| 16 | 7704355 | 8341012 | 2224 | 218 | RA | HF | -0.638 | -1.000 | -0.026 | 0.407 | 0.006 | 1.000 | 0.042 | 0.355 |
| 16 | 25235252 | 26154122 | 2188 | 206 | RA | HF | 0.441 | 0.052 | 0.954 | 0.195 | 0.005 | 0.910 | 0.032 | 0.350 |
| 16 | 57119890 | 58508978 | 2937 | 280 | RA | HF | -0.533 | -1.000 | -0.064 | 0.284 | 0.008 | 1.000 | 0.028 | 0.334 |
| 16 | 75525960 | 76635467 | 2323 | 174 | RA | HF | -0.484 | -1.000 | -0.055 | 0.234 | 0.006 | 1.000 | 0.036 | 0.350 |
| 16 | 83077870 | 83778371 | 2310 | 267 | RA | HF | -0.656 | -1.000 | -0.130 | 0.431 | 0.019 | 1.000 | 0.018 | 0.294 |
| 17 | 2699136 | 3430440 | 1676 | 196 | RA | HF | -0.589 | -1.000 | -0.254 | 0.347 | 0.065 | 1.000 | 0.001 | 0.090 |
| 17 | 12676354 | 13648446 | 2485 | 261 | RA | HF | 0.478 | 0.096 | 0.969 | 0.229 | 0.011 | 0.939 | 0.015 | 0.285 |
| 17 | 14508611 | 15308722 | 2168 | 223 | RA | HF | -0.621 | -1.000 | -0.056 | 0.386 | 0.009 | 1.000 | 0.041 | 0.355 |
| 17 | 15308723 | 16405207 | 1321 | 123 | RA | HF | -0.358 | -0.640 | -0.086 | 0.128 | 0.008 | 0.409 | 0.011 | 0.249 |
| 17 | 40233546 | 42348003 | 2364 | 187 | RA | HF | 0.578 | 0.043 | 1.000 | 0.334 | 0.007 | 1.000 | 0.041 | 0.355 |
| 17 | 58652810 | 60453120 | 1824 | 189 | RA | HF | -0.818 | -1.000 | -0.278 | 0.669 | 0.077 | 1.000 | 0.004 | 0.191 |
| 17 | 73741322 | 74908266 | 2215 | 215 | RA | HF | 0.544 | 0.098 | 1.000 | 0.296 | 0.013 | 1.000 | 0.021 | 0.302 |
| 18 | 6756497 | 7862479 | 2117 | 256 | RA | HF | -0.302 | -0.581 | -0.047 | 0.091 | 0.003 | 0.338 | 0.020 | 0.297 |
| 18 | 13641480 | 14439251 | 1102 | 81 | RA | HF | 0.678 | 0.153 | 1.000 | 0.460 | 0.026 | 1.000 | 0.016 | 0.285 |
| 18 | 55809316 | 56832312 | 2173 | 226 | RA | HF | -0.470 | -0.793 | -0.182 | 0.221 | 0.033 | 0.628 | 0.001 | 0.104 |
| 18 | 73568261 | 74471745 | 2239 | 256 | RA | HF | -0.526 | -0.959 | -0.177 | 0.277 | 0.032 | 0.920 | 0.004 | 0.175 |
| 19 | 2253175 | 3085446 | 1887 | 253 | RA | HF | 0.472 | 0.037 | 1.000 | 0.222 | 0.005 | 1.000 | 0.035 | 0.350 |
| 19 | 30375793 | 31763008 | 2020 | 235 | RA | HF | 0.455 | 0.080 | 0.923 | 0.207 | 0.009 | 0.853 | 0.020 | 0.297 |
| 19 | 43317283 | 43960958 | 1446 | 85 | RA | HF | 0.530 | 0.216 | 0.885 | 0.280 | 0.047 | 0.784 | 0.002 | 0.129 |
| 19 | 45893308 | 46765060 | 1543 | 185 | RA | HF | -0.688 | -1.000 | -0.181 | 0.473 | 0.035 | 1.000 | 0.011 | 0.252 |
| 19 | 46765061 | 47752727 | 1546 | 204 | RA | HF | 0.412 | 0.112 | 0.787 | 0.169 | 0.013 | 0.619 | 0.009 | 0.236 |
| 19 | 53431218 | 54042240 | 1903 | 244 | RA | HF | -0.292 | -0.491 | -0.097 | 0.085 | 0.009 | 0.241 | 0.004 | 0.187 |
| 19 | 56411087 | 57143509 | 1787 | 243 | RA | HF | -0.258 | -0.487 | -0.034 | 0.067 | 0.002 | 0.238 | 0.023 | 0.310 |
| 19 | 57143510 | 57959952 | 1828 | 230 | RA | HF | 0.352 | 0.082 | 0.632 | 0.124 | 0.007 | 0.399 | 0.011 | 0.252 |
| 20 | 17359291 | 18058524 | 1693 | 200 | RA | HF | 0.667 | 0.180 | 1.000 | 0.445 | 0.033 | 1.000 | 0.009 | 0.240 |
| 20 | 53879804 | 54743694 | 1599 | 150 | RA | HF | 0.471 | 0.050 | 1.000 | 0.222 | 0.006 | 1.000 | 0.033 | 0.350 |
| 21 | 15026793 | 15840033 | 1423 | 103 | RA | HF | 1.000 | 0.464 | 1.000 | 1.000 | 0.216 | 1.000 | 0.001 | 0.090 |
| 22 | 25282437 | 26166934 | 1544 | 207 | RA | HF | -0.332 | -0.648 | -0.047 | 0.110 | 0.003 | 0.420 | 0.027 | 0.330 |
| 22 | 38718590 | 40378783 | 2585 | 244 | RA | HF | 0.456 | 0.190 | 0.751 | 0.208 | 0.036 | 0.564 | 0.001 | 0.090 |
| 22 | 47299626 | 48269177 | 2283 | 234 | RA | HF | 0.397 | 0.062 | 0.800 | 0.158 | 0.005 | 0.640 | 0.023 | 0.308 |
| 22 | 48977539 | 49559207 | 2375 | 233 | RA | HF | -0.610 | -1.000 | -0.158 | 0.372 | 0.026 | 1.000 | 0.011 | 0.249 |
| 1 | 17557747 | 18427820 | 1907 | 202 | RA | Stroke | 0.409 | 0.054 | 0.885 | 0.167 | 0.005 | 0.783 | 0.028 | 0.304 |
| 1 | 43512670 | 45167235 | 2499 | 219 | RA | Stroke | -0.500 | -1.000 | -0.095 | 0.250 | 0.011 | 1.000 | 0.017 | 0.244 |
| 1 | 57529671 | 58387483 | 1821 | 175 | RA | Stroke | 0.730 | 0.225 | 1.000 | 0.532 | 0.051 | 1.000 | 0.008 | 0.206 |
| 1 | 62778734 | 64344226 | 2789 | 228 | RA | Stroke | -0.575 | -1.000 | -0.218 | 0.330 | 0.048 | 1.000 | 0.003 | 0.147 |
| 1 | 65894185 | 66778015 | 1765 | 104 | RA | Stroke | 0.924 | 0.215 | 1.000 | 0.854 | 0.052 | 1.000 | 0.019 | 0.251 |
| 1 | 109271450 | 110224230 | 1571 | 158 | RA | Stroke | 0.753 | 0.232 | 1.000 | 0.567 | 0.055 | 1.000 | 0.008 | 0.206 |
| 1 | 167163955 | 168179086 | 1816 | 219 | RA | Stroke | -0.579 | -1.000 | -0.124 | 0.336 | 0.017 | 1.000 | 0.016 | 0.238 |
| 1 | 201067953 | 202583884 | 2814 | 291 | RA | Stroke | 0.472 | 0.027 | 1.000 | 0.223 | 0.004 | 1.000 | 0.042 | 0.352 |
| 1 | 213958293 | 214783872 | 1504 | 179 | RA | Stroke | 0.706 | 0.153 | 1.000 | 0.499 | 0.026 | 1.000 | 0.016 | 0.238 |
| 1 | 222763598 | 223654902 | 1524 | 136 | RA | Stroke | -0.670 | -1.000 | -0.121 | 0.449 | 0.018 | 1.000 | 0.023 | 0.275 |
| 1 | 241033585 | 241813534 | 1667 | 215 | RA | Stroke | 0.646 | 0.077 | 1.000 | 0.417 | 0.010 | 1.000 | 0.028 | 0.304 |
| 1 | 244723197 | 245970505 | 2633 | 306 | RA | Stroke | 0.486 | 0.058 | 1.000 | 0.236 | 0.006 | 1.000 | 0.026 | 0.300 |
| 2 | 5568654 | 6306606 | 1845 | 192 | RA | Stroke | 0.600 | 0.127 | 1.000 | 0.360 | 0.020 | 1.000 | 0.014 | 0.238 |
| 2 | 11230350 | 11995765 | 1523 | 187 | RA | Stroke | 0.643 | 0.084 | 1.000 | 0.414 | 0.013 | 1.000 | 0.024 | 0.286 |
| 2 | 33123605 | 33735318 | 1359 | 191 | RA | Stroke | -0.602 | -1.000 | -0.035 | 0.362 | 0.005 | 1.000 | 0.043 | 0.353 |
| 2 | 38887394 | 40087418 | 1453 | 145 | RA | Stroke | 0.917 | 0.419 | 1.000 | 0.840 | 0.175 | 1.000 | 0.001 | 0.115 |
| 2 | 131760639 | 132598285 | 972 | 61 | RA | Stroke | 1.000 | 0.539 | 1.000 | 1.000 | 0.290 | 1.000 | 0.000 | 0.029 |
| 2 | 181309940 | 182307877 | 1847 | 132 | RA | Stroke | -0.724 | -1.000 | -0.241 | 0.525 | 0.058 | 1.000 | 0.006 | 0.206 |
| 2 | 241113280 | 241910366 | 2101 | 212 | RA | Stroke | 0.822 | 0.208 | 1.000 | 0.675 | 0.045 | 1.000 | 0.014 | 0.238 |
| 3 | 17182495 | 19030988 | 2793 | 160 | RA | Stroke | 0.834 | 0.269 | 1.000 | 0.696 | 0.073 | 1.000 | 0.006 | 0.206 |
| 3 | 25516194 | 26905340 | 2430 | 172 | RA | Stroke | -0.624 | -1.000 | -0.019 | 0.389 | 0.007 | 1.000 | 0.049 | 0.366 |
| 3 | 45844192 | 47588461 | 2561 | 159 | RA | Stroke | 0.679 | 0.260 | 1.000 | 0.461 | 0.067 | 1.000 | 0.003 | 0.147 |
| 3 | 100552523 | 101906989 | 2353 | 137 | RA | Stroke | 0.578 | 0.059 | 1.000 | 0.334 | 0.007 | 1.000 | 0.034 | 0.323 |
| 3 | 115649910 | 116498570 | 1502 | 123 | RA | Stroke | -0.740 | -1.000 | -0.161 | 0.548 | 0.031 | 1.000 | 0.015 | 0.238 |
| 3 | 132124019 | 132925967 | 1505 | 102 | RA | Stroke | 1.000 | 0.401 | 1.000 | 1.000 | 0.161 | 1.000 | 0.000 | 0.075 |
| 3 | 139831268 | 141034637 | 2128 | 197 | RA | Stroke | 1.000 | 0.165 | 1.000 | 1.000 | 0.038 | 1.000 | 0.008 | 0.206 |
| 3 | 148638309 | 149998411 | 2593 | 253 | RA | Stroke | 0.748 | 0.276 | 1.000 | 0.559 | 0.077 | 1.000 | 0.003 | 0.147 |
| 3 | 172004761 | 173415591 | 2440 | 247 | RA | Stroke | -0.780 | -1.000 | -0.319 | 0.609 | 0.102 | 1.000 | 0.001 | 0.115 |
| 3 | 174684708 | 175671424 | 2127 | 183 | RA | Stroke | -0.731 | -1.000 | -0.137 | 0.535 | 0.022 | 1.000 | 0.022 | 0.273 |
| 3 | 186602046 | 187939199 | 2785 | 289 | RA | Stroke | 0.612 | 0.167 | 1.000 | 0.374 | 0.028 | 1.000 | 0.010 | 0.215 |
| 4 | 37880861 | 38984838 | 2651 | 255 | RA | Stroke | -0.386 | -0.812 | -0.042 | 0.149 | 0.003 | 0.660 | 0.030 | 0.304 |
| 4 | 76497359 | 78045637 | 3317 | 205 | RA | Stroke | -0.614 | -1.000 | -0.077 | 0.378 | 0.010 | 1.000 | 0.032 | 0.310 |
| 4 | 89244555 | 90236971 | 1796 | 158 | RA | Stroke | -1.000 | -1.000 | -0.120 | 1.000 | 0.029 | 1.000 | 0.026 | 0.300 |
| 4 | 178314528 | 179233905 | 1879 | 147 | RA | Stroke | 0.756 | 0.068 | 1.000 | 0.572 | 0.014 | 1.000 | 0.036 | 0.330 |
| 4 | 186881569 | 187985393 | 2642 | 291 | RA | Stroke | 0.511 | 0.079 | 1.000 | 0.261 | 0.009 | 1.000 | 0.025 | 0.297 |
| 4 | 187985394 | 189153279 | 2821 | 268 | RA | Stroke | 0.740 | 0.135 | 1.000 | 0.548 | 0.022 | 1.000 | 0.021 | 0.267 |
| 5 | 598321 | 1206609 | 982 | 154 | RA | Stroke | -0.632 | -1.000 | -0.245 | 0.400 | 0.060 | 1.000 | 0.002 | 0.147 |
| 5 | 21211400 | 22149042 | 1109 | 84 | RA | Stroke | 0.809 | 0.126 | 1.000 | 0.655 | 0.027 | 1.000 | 0.029 | 0.304 |
| 5 | 33971057 | 35157146 | 2111 | 181 | RA | Stroke | 0.904 | 0.419 | 1.000 | 0.818 | 0.176 | 1.000 | 0.001 | 0.082 |
| 5 | 52139774 | 52837225 | 1682 | 120 | RA | Stroke | 0.915 | 0.544 | 1.000 | 0.837 | 0.296 | 1.000 | 0.000 | 0.018 |
| 5 | 55968967 | 56896890 | 1586 | 169 | RA | Stroke | -0.685 | -1.000 | -0.155 | 0.469 | 0.027 | 1.000 | 0.014 | 0.238 |
| 5 | 115117632 | 116208000 | 2638 | 231 | RA | Stroke | 0.729 | 0.052 | 1.000 | 0.531 | 0.010 | 1.000 | 0.038 | 0.338 |
| 5 | 166863414 | 168342743 | 2596 | 291 | RA | Stroke | 0.534 | 0.197 | 0.963 | 0.285 | 0.039 | 0.927 | 0.003 | 0.147 |
| 5 | 170898341 | 172285682 | 2696 | 308 | RA | Stroke | -0.404 | -0.795 | -0.075 | 0.163 | 0.007 | 0.632 | 0.020 | 0.265 |
| 5 | 172285683 | 173606995 | 2806 | 273 | RA | Stroke | 0.573 | 0.175 | 1.000 | 0.328 | 0.031 | 1.000 | 0.008 | 0.206 |
| 6 | 23192338 | 23939306 | 1925 | 190 | RA | Stroke | -0.383 | -0.802 | -0.043 | 0.147 | 0.003 | 0.643 | 0.033 | 0.317 |
| 6 | 32682214 | 32897998 | 1799 | 62 | RA | Stroke | -0.284 | -0.605 | -0.010 | 0.081 | 0.001 | 0.367 | 0.049 | 0.366 |
| 6 | 42103739 | 43770626 | 2297 | 234 | RA | Stroke | 0.593 | 0.124 | 1.000 | 0.352 | 0.017 | 1.000 | 0.017 | 0.245 |
| 6 | 131457165 | 132890694 | 2477 | 208 | RA | Stroke | 1.000 | 0.226 | 1.000 | 1.000 | 0.056 | 1.000 | 0.006 | 0.201 |
| 6 | 148184845 | 149278798 | 2710 | 277 | RA | Stroke | 0.592 | 0.034 | 1.000 | 0.350 | 0.006 | 1.000 | 0.043 | 0.353 |
| 6 | 154462011 | 155523957 | 2237 | 271 | RA | Stroke | -0.320 | -0.672 | -0.009 | 0.102 | 0.002 | 0.452 | 0.047 | 0.361 |
| 6 | 156762741 | 158220143 | 1895 | 239 | RA | Stroke | -0.452 | -1.000 | -0.051 | 0.204 | 0.005 | 1.000 | 0.030 | 0.304 |
| 6 | 158952061 | 159604052 | 1577 | 145 | RA | Stroke | -0.526 | -1.000 | -0.064 | 0.276 | 0.008 | 1.000 | 0.032 | 0.310 |
| 7 | 1366973 | 2473749 | 2526 | 193 | RA | Stroke | 0.477 | 0.031 | 1.000 | 0.228 | 0.004 | 1.000 | 0.044 | 0.353 |
| 7 | 17596579 | 19131026 | 2650 | 278 | RA | Stroke | -0.442 | -0.930 | -0.051 | 0.195 | 0.004 | 0.864 | 0.028 | 0.304 |
| 7 | 128779174 | 130418704 | 2423 | 220 | RA | Stroke | 0.656 | 0.100 | 1.000 | 0.431 | 0.015 | 1.000 | 0.030 | 0.304 |
| 7 | 130418705 | 131856481 | 2701 | 278 | RA | Stroke | 0.488 | 0.171 | 0.863 | 0.239 | 0.029 | 0.744 | 0.004 | 0.147 |
| 7 | 148622914 | 149842999 | 1894 | 177 | RA | Stroke | -0.527 | -1.000 | -0.018 | 0.277 | 0.004 | 1.000 | 0.045 | 0.354 |
| 7 | 152253295 | 153241228 | 2556 | 244 | RA | Stroke | -0.474 | -1.000 | -0.042 | 0.225 | 0.004 | 1.000 | 0.034 | 0.322 |
| 8 | 12947936 | 13395051 | 1327 | 120 | RA | Stroke | 0.944 | 0.258 | 1.000 | 0.892 | 0.072 | 1.000 | 0.013 | 0.238 |
| 8 | 18668610 | 19488888 | 2166 | 254 | RA | Stroke | -0.448 | -1.000 | -0.024 | 0.201 | 0.003 | 1.000 | 0.040 | 0.347 |
| 8 | 92876621 | 94999066 | 2834 | 236 | RA | Stroke | 0.759 | 0.274 | 1.000 | 0.577 | 0.075 | 1.000 | 0.004 | 0.147 |
| 8 | 119423299 | 120216587 | 1632 | 139 | RA | Stroke | 0.846 | 0.203 | 1.000 | 0.715 | 0.044 | 1.000 | 0.017 | 0.244 |
| 9 | 97418634 | 98885862 | 2247 | 190 | RA | Stroke | 0.782 | 0.178 | 1.000 | 0.612 | 0.035 | 1.000 | 0.018 | 0.251 |
| 9 | 117054910 | 117989017 | 2032 | 215 | RA | Stroke | -0.490 | -1.000 | -0.037 | 0.240 | 0.005 | 1.000 | 0.040 | 0.347 |
| 9 | 132999453 | 134141936 | 1944 | 270 | RA | Stroke | 0.728 | 0.300 | 1.000 | 0.530 | 0.090 | 1.000 | 0.002 | 0.118 |
| 9 | 136042491 | 136770926 | 1950 | 219 | RA | Stroke | -0.402 | -0.753 | -0.098 | 0.162 | 0.010 | 0.567 | 0.012 | 0.238 |
| 10 | 2637066 | 3225336 | 1727 | 204 | RA | Stroke | -0.874 | -1.000 | -0.242 | 0.763 | 0.061 | 1.000 | 0.010 | 0.215 |
| 10 | 25577762 | 26161974 | 1782 | 110 | RA | Stroke | -0.779 | -1.000 | -0.033 | 0.607 | 0.011 | 1.000 | 0.048 | 0.364 |
| 10 | 33655459 | 35178224 | 2707 | 238 | RA | Stroke | 1.000 | 0.567 | 1.000 | 1.000 | 0.322 | 1.000 | 0.000 | 0.005 |
| 10 | 104206838 | 106142283 | 2900 | 203 | RA | Stroke | 0.534 | 0.046 | 1.000 | 0.285 | 0.007 | 1.000 | 0.034 | 0.323 |
| 10 | 121874446 | 122803506 | 2212 | 209 | RA | Stroke | -0.528 | -1.000 | -0.015 | 0.279 | 0.005 | 1.000 | 0.044 | 0.354 |
| 10 | 130694009 | 131694738 | 2207 | 227 | RA | Stroke | -0.887 | -1.000 | -0.216 | 0.786 | 0.051 | 1.000 | 0.015 | 0.238 |
| 11 | 11632651 | 12470449 | 2083 | 267 | RA | Stroke | -0.451 | -0.975 | -0.106 | 0.203 | 0.012 | 0.950 | 0.012 | 0.238 |
| 11 | 124403478 | 125266684 | 1539 | 224 | RA | Stroke | 0.395 | 0.012 | 0.862 | 0.156 | 0.002 | 0.744 | 0.047 | 0.361 |
| 11 | 127306872 | 128262550 | 1860 | 160 | RA | Stroke | 0.533 | 0.118 | 1.000 | 0.284 | 0.016 | 1.000 | 0.015 | 0.238 |
| 12 | 4967644 | 6023700 | 2241 | 265 | RA | Stroke | 0.746 | 0.163 | 1.000 | 0.557 | 0.027 | 1.000 | 0.016 | 0.244 |
| 12 | 25058715 | 25990813 | 2154 | 198 | RA | Stroke | 0.537 | 0.117 | 1.000 | 0.289 | 0.015 | 1.000 | 0.015 | 0.238 |
| 12 | 46037745 | 47499603 | 1975 | 158 | RA | Stroke | 0.542 | 0.027 | 1.000 | 0.294 | 0.006 | 1.000 | 0.040 | 0.347 |
| 12 | 66114644 | 67183620 | 1883 | 177 | RA | Stroke | 0.931 | 0.294 | 1.000 | 0.867 | 0.088 | 1.000 | 0.009 | 0.206 |
| 12 | 96017783 | 96828314 | 1640 | 199 | RA | Stroke | -0.495 | -1.000 | -0.061 | 0.245 | 0.006 | 1.000 | 0.031 | 0.307 |
| 12 | 128372589 | 129170296 | 1995 | 261 | RA | Stroke | -0.668 | -1.000 | -0.151 | 0.446 | 0.025 | 1.000 | 0.014 | 0.238 |
| 13 | 28587126 | 30068525 | 2991 | 248 | RA | Stroke | 0.588 | 0.184 | 1.000 | 0.345 | 0.034 | 1.000 | 0.007 | 0.206 |
| 13 | 32986687 | 34722391 | 2986 | 222 | RA | Stroke | -0.592 | -1.000 | -0.032 | 0.350 | 0.007 | 1.000 | 0.043 | 0.353 |
| 13 | 48137480 | 49381727 | 2156 | 121 | RA | Stroke | 0.515 | 0.028 | 1.000 | 0.265 | 0.005 | 1.000 | 0.046 | 0.359 |
| 13 | 49381728 | 50419896 | 1893 | 159 | RA | Stroke | -0.645 | -1.000 | -0.129 | 0.416 | 0.019 | 1.000 | 0.019 | 0.251 |
| 13 | 114302562 | 115109851 | 1257 | 141 | RA | Stroke | 0.687 | 0.108 | 1.000 | 0.471 | 0.018 | 1.000 | 0.026 | 0.300 |
| 14 | 97174315 | 98268391 | 1985 | 219 | RA | Stroke | 0.880 | 0.324 | 1.000 | 0.774 | 0.106 | 1.000 | 0.003 | 0.147 |
| 14 | 99474534 | 100786189 | 2390 | 270 | RA | Stroke | 0.629 | 0.291 | 1.000 | 0.396 | 0.085 | 1.000 | 0.000 | 0.075 |
| 15 | 36583328 | 37962915 | 2551 | 260 | RA | Stroke | 0.323 | 0.022 | 0.651 | 0.105 | 0.002 | 0.424 | 0.038 | 0.338 |
| 15 | 64339946 | 66355634 | 2334 | 161 | RA | Stroke | 0.510 | 0.161 | 0.952 | 0.260 | 0.026 | 0.905 | 0.007 | 0.206 |
| 15 | 82191078 | 84209760 | 1793 | 188 | RA | Stroke | 0.476 | 0.143 | 0.865 | 0.226 | 0.021 | 0.748 | 0.008 | 0.206 |
| 15 | 84209761 | 85494083 | 1823 | 166 | RA | Stroke | 0.524 | 0.022 | 1.000 | 0.274 | 0.005 | 1.000 | 0.050 | 0.366 |
| 15 | 86341599 | 87158587 | 1936 | 182 | RA | Stroke | -0.722 | -1.000 | -0.142 | 0.521 | 0.022 | 1.000 | 0.019 | 0.251 |
| 15 | 96864279 | 98025684 | 2093 | 259 | RA | Stroke | 0.537 | 0.034 | 1.000 | 0.289 | 0.006 | 1.000 | 0.038 | 0.338 |
| 16 | 65882821 | 66738843 | 1576 | 193 | RA | Stroke | 0.569 | 0.034 | 1.000 | 0.323 | 0.006 | 1.000 | 0.044 | 0.354 |
| 16 | 72089512 | 73140781 | 1343 | 189 | RA | Stroke | 0.462 | 0.082 | 1.000 | 0.213 | 0.008 | 1.000 | 0.019 | 0.251 |
| 16 | 73140782 | 74135275 | 1881 | 273 | RA | Stroke | 0.577 | 0.049 | 1.000 | 0.333 | 0.008 | 1.000 | 0.036 | 0.330 |
| 16 | 83077870 | 83778371 | 2323 | 270 | RA | Stroke | 0.676 | 0.071 | 1.000 | 0.457 | 0.013 | 1.000 | 0.028 | 0.304 |
| 16 | 87669169 | 88387228 | 1721 | 214 | RA | Stroke | 0.723 | 0.295 | 1.000 | 0.522 | 0.087 | 1.000 | 0.001 | 0.115 |
| 17 | 36116885 | 37361178 | 1721 | 219 | RA | Stroke | -0.633 | -1.000 | -0.216 | 0.400 | 0.047 | 1.000 | 0.004 | 0.162 |
| 17 | 45883902 | 47516224 | 2772 | 189 | RA | Stroke | 0.713 | 0.247 | 1.000 | 0.508 | 0.061 | 1.000 | 0.005 | 0.194 |
| 18 | 10719 | 848350 | 1848 | 253 | RA | Stroke | 0.618 | 0.128 | 1.000 | 0.382 | 0.020 | 1.000 | 0.015 | 0.238 |
| 18 | 6756497 | 7862479 | 2162 | 264 | RA | Stroke | 0.570 | 0.270 | 0.925 | 0.325 | 0.073 | 0.856 | 0.000 | 0.075 |
| 18 | 12735559 | 13641479 | 1724 | 221 | RA | Stroke | -0.416 | -1.000 | -0.036 | 0.173 | 0.004 | 1.000 | 0.036 | 0.330 |
| 18 | 42974166 | 44300145 | 2587 | 254 | RA | Stroke | -0.519 | -1.000 | -0.085 | 0.269 | 0.010 | 1.000 | 0.021 | 0.267 |
| 19 | 8199017 | 9105577 | 1776 | 201 | RA | Stroke | -0.471 | -1.000 | -0.099 | 0.222 | 0.011 | 1.000 | 0.015 | 0.238 |
| 19 | 10028841 | 11681978 | 2611 | 268 | RA | Stroke | -0.425 | -0.832 | -0.115 | 0.180 | 0.013 | 0.693 | 0.008 | 0.206 |
| 19 | 16079007 | 17045963 | 1670 | 162 | RA | Stroke | 0.503 | 0.060 | 1.000 | 0.253 | 0.007 | 1.000 | 0.029 | 0.304 |
| 19 | 45893308 | 46765060 | 1552 | 187 | RA | Stroke | 0.964 | 0.261 | 1.000 | 0.930 | 0.074 | 1.000 | 0.011 | 0.228 |
| 19 | 50451926 | 51259178 | 1306 | 206 | RA | Stroke | -0.518 | -1.000 | -0.141 | 0.268 | 0.021 | 1.000 | 0.009 | 0.212 |
| 20 | 9279551 | 10279603 | 2166 | 238 | RA | Stroke | -0.829 | -1.000 | -0.329 | 0.687 | 0.108 | 1.000 | 0.002 | 0.147 |
| 20 | 13202336 | 14203755 | 1697 | 149 | RA | Stroke | 0.665 | 0.116 | 1.000 | 0.442 | 0.018 | 1.000 | 0.022 | 0.274 |
| 20 | 17359291 | 18058524 | 1701 | 202 | RA | Stroke | -0.685 | -1.000 | -0.202 | 0.469 | 0.042 | 1.000 | 0.007 | 0.206 |
| 20 | 43008891 | 44072210 | 1619 | 199 | RA | Stroke | -0.589 | -1.000 | -0.203 | 0.347 | 0.041 | 1.000 | 0.004 | 0.147 |
| 20 | 47203837 | 48212975 | 2012 | 139 | RA | Stroke | -0.833 | -1.000 | -0.045 | 0.694 | 0.012 | 1.000 | 0.041 | 0.352 |
| 20 | 49236419 | 50653620 | 3099 | 291 | RA | Stroke | -0.467 | -0.947 | -0.098 | 0.218 | 0.011 | 0.897 | 0.016 | 0.238 |
| 20 | 50653621 | 51533750 | 1761 | 118 | RA | Stroke | -0.504 | -1.000 | -0.130 | 0.254 | 0.017 | 1.000 | 0.011 | 0.238 |
| 21 | 33270273 | 34383794 | 1987 | 210 | RA | Stroke | 0.480 | 0.098 | 0.975 | 0.231 | 0.011 | 0.950 | 0.016 | 0.238 |
| 21 | 43081435 | 43812791 | 2232 | 309 | RA | Stroke | 0.413 | 0.157 | 0.700 | 0.171 | 0.025 | 0.490 | 0.003 | 0.147 |
| 22 | 19635655 | 20969184 | 1966 | 239 | RA | Stroke | -0.514 | -1.000 | -0.016 | 0.264 | 0.004 | 1.000 | 0.046 | 0.360 |
| 22 | 32662294 | 33501725 | 2200 | 224 | RA | Stroke | 0.581 | 0.074 | 1.000 | 0.338 | 0.010 | 1.000 | 0.029 | 0.304 |
| 22 | 48269178 | 48977538 | 2069 | 269 | RA | Stroke | -0.711 | -1.000 | -0.309 | 0.505 | 0.095 | 1.000 | 0.001 | 0.115 |

**Table S8.** The results of model fit in MeXiR analysis.

| Traits | RA and stroke | RA and HF | | RA and AF | RA and CAD |
| --- | --- | --- | --- | --- | --- |
| dice (mean) | 0.407 | 0.242 | | 0.123 | 0.23 |
| dice (std) | 0.151 | 0.108 | | 0.04 | 0.088 |
| pi1 (mean) | 1.40×10^-4^ | 1.40×10^-4^ | | 2.30×10^-4^ | 1.80×10^-4^ |
| pi1 (std) | 4.90×10^-5^ | 5.46×10^-5^ | | 2.01×10^-5^ | 3.91×10^-5^ |
| pi2 (mean) | 2.10 ×10^-4^ | 5.80 ×10^-4^ | | 1.2 ×10^-4^ | 3.5 ×10^-4^ |
| pi2 (std) | 4.73×10^-5^ | 7.59×10^-5^ | | 1.36×10^-5^ | 3.73×10^-5^ |
| pi12 (mean) | 0.00012 | 0.00012 | | 2.51×10^-5^ | 7.87×10^-5^ |
| pi12 (std) | 4.63×10^-5^ | 5.75×10^-5^ | | 8.36×10^-6^ | 3.01×10^-5^ |
| nc1@p9 (mean) | 439.526 | 451.778 | | 740.572 | 569.572 |
| nc1@p9 (std) | 156.14 | 173.972 | | 64.231 | 124.748 |
| nc2@p9 (mean) | 660.345 | 1834.84 | | 390.283 | 1112.24 |
| nc2@p9 (std) | 150.979 | 242.125 | | 43.3267 | 118.991 |
| nc12@p9 (mean) | 380.998 | 368.746 | 79.9522 | | 250.952 |
| nc12@p9 (std) | 147.666 | 183.46 | 26.648 | | 96.047 |
| rho_zero (mean) | 0.013 | 0.007 | 0.012 | | 0.009 |
| rho_zero (std) | 0.002 | 0.002 | 0.002 | | 0.002 |
| rho_beta (mean) | 0.619 | 0.494 | 0.605 | | 0.493 |
| rho_beta (std) | 0.239 | 0.219 | 0.189 | | 0.228 |
| rg (mean) | 0.22 | 0.112 | 0.071 | | 0.099 |
| rg (std) | 0.026 | 0.019 | 0.013 | | 0.015 |
| fraction_concordant_within_shared (mean) | 0.731 | 0.673 | 0.718 | | 0.674 |
| fraction_concordant_within_shared (std) | 0.115 | 0.092 | 0.089 | | 0.098 |
| best_vs_min_AIC | 4.24 | 4.25 | 5.606 | | 5.793 |
| best_vs_max_AIC | 9.023 | 7.37 | 97.812 | | 42.929 |

Abbreviations: AF, atrial fibrillation; CAD, coronary artery disease; HF, heart failure; RA, rheumatoid arthritis.

**Table S9.** The independent pleiotropic loci based on conjFDR analyses.

| Lead SNP | CHR | Lead BP | Min BP | Max BP | Trait 1 | Trait 2 | FDR |
| --- | --- | --- | --- | --- | --- | --- | --- |
| rs12022363 | 1 | 38252609 | 38226776 | 38363620 | RA | HF | 0.017875951 |
| rs62149420 | 2 | 61094810 | 60939032 | 61504416 | RA | HF | 0.013785691 |
| rs2905734 | 6 | 31453711 | 31321753 | 32136029 | RA | HF | 0.003245988 |
| rs10774624 | 12 | 111833788 | 111826477 | 112985328 | RA | HF | 4.11602E-05 |
| rs11658278 | 17 | 38031164 | 37902887 | 38089717 | RA | HF | 0.027814022 |
| rs28885132 | 1 | 38371691 | 38258644 | 38379018 | RA | AF | 0.043035864 |
| rs12126142 | 1 | 154425456 | 154395212 | 154428283 | RA | AF | 0.001122485 |
| rs62149420 | 2 | 61094810 | 60939032 | 61504416 | RA | AF | 0.014687622 |
| rs4563251 | 2 | 70251490 | 70196019 | 70368391 | RA | AF | 0.013101881 |
| rs700677 | 2 | 198702424 | 198258388 | 198954774 | RA | AF | 0.018450507 |
| rs2454429 | 3 | 12620720 | 12611269 | 12738547 | RA | AF | 0.033658204 |
| rs409558 | 6 | 31708147 | 31373260 | 33761462 | RA | AF | 0.008322996 |
| rs42034 | 7 | 92239144 | 92236164 | 92327026 | RA | AF | 0.000129087 |
| rs13277738 | 8 | 18241507 | 18239697 | 18283352 | RA | AF | 0.027498745 |
| rs7201780 | 16 | 30147265 | 30120442 | 30164488 | RA | AF | 0.024593981 |
| rs9747973 | 17 | 37905107 | 37389409 | 38119638 | RA | AF | 3.67893E-06 |
| rs2297199 | 20 | 44674743 | 44674743 | 44729089 | RA | AF | 0.019937899 |
| rs2772373 | 6 | 33429672 | 29648730 | 33472317 | RA | stroke | 0.027989452 |
| rs42039 | 7 | 92244422 | 92223518 | 92327026 | RA | stroke | 0.000156955 |
| rs7085041 | 10 | 31118426 | 31113388 | 31130241 | RA | stroke | 0.026067566 |
| rs10774624 | 12 | 111833788 | 111684461 | 112985328 | RA | stroke | 3.20243E-05 |
| rs7248558 | 19 | 10770292 | 10744807 | 10835405 | RA | stroke | 0.033338617 |
| rs6074012 | 20 | 44700166 | 44680412 | 44747947 | RA | stroke | 0.006195351 |
| rs2843151 | 1 | 2245633 | 2243518 | 2252400 | RA | CAD | 0.037701542 |
| rs11552449 | 1 | 114448389 | 114075796 | 114451425 | RA | CAD | 0.002531783 |
| rs12126142 | 1 | 154425456 | 154395212 | 154428283 | RA | CAD | 0.000782735 |
| rs35086414 | 1 | 197800965 | 197312162 | 197822172 | RA | CAD | 0.026722987 |
| rs3130683 | 6 | 31888367 | 29809860 | 32797361 | RA | CAD | 1.51579E-06 |
| rs6929470 | 6 | 34720324 | 34549699 | 34854179 | RA | CAD | 0.040364225 |
| rs13277738 | 8 | 18241507 | 18239697 | 18291590 | RA | CAD | 0.01082409 |
| rs4750517 | 10 | 6520458 | 6508377 | 6531055 | RA | CAD | 0.042866928 |
| rs4749532 | 10 | 30418323 | 30402103 | 30445666 | RA | CAD | 0.034351214 |
| rs7098414 | 10 | 82214586 | 82013134 | 82287527 | RA | CAD | 0.002535983 |
| rs10840298 | 11 | 9764832 | 9750757 | 9811616 | RA | CAD | 0.049949941 |
| rs174541 | 11 | 61565908 | 61542006 | 61624181 | RA | CAD | 0.023963274 |
| rs10774624 | 12 | 111833788 | 111708458 | 112985328 | RA | CAD | 4.24032E-05 |
| rs12919951 | 16 | 75306890 | 75304497 | 75491327 | RA | CAD | 0.02351858 |
| rs8075737 | 17 | 37727316 | 37390863 | 37745979 | RA | CAD | 0.04694739 |
| rs34891485 | 17 | 40263458 | 40244377 | 40356360 | RA | CAD | 0.018238189 |
| rs7248558 | 19 | 10770292 | 10744807 | 10835405 | RA | CAD | 0.0189025 |
| rs2421206 | 19 | 11262477 | 11224181 | 11277232 | RA | CAD | 0.002427141 |
| rs4408777 | 20 | 62706105 | 62692060 | 62712053 | RA | CAD | 0.048851794 |

Abbreviations: AF, atrial fibrillation; CAD, coronary artery disease; HF, heart failure; RA, rheumatoid arthritis.

**Table S10.** The independent pleiotropic loci based on ASSET analyses.

| SNP | CHR | BP | Trait 1 | Trait 2 | *P* |
| --- | --- | --- | --- | --- | --- |
| rs34331363 | 6 | 32568439 | RA | stroke | 0 |
| rs9271527 | 6 | 32589867 | RA | stroke | 4.38×10^-242^ |
| rs41316548 | 6 | 32797167 | RA | stroke | 1.34×10^-227^ |
| rs34562262 | 6 | 32020961 | RA | stroke | 2.98×10^-214^ |
| rs4713555 | 6 | 32575524 | RA | stroke | 1.32×10^-192^ |
| rs17421624 | 6 | 32066177 | RA | stroke | 4.08×10^-183^ |
| rs2476601 | 1 | 114377568 | RA | stroke | 4.98×10^-149^ |
| rs2071479 | 6 | 32781112 | RA | stroke | 5.35×10^-147^ |
| rs9271376 | 6 | 32587113 | RA | stroke | 5.89×10^-137^ |
| rs3819720 | 6 | 32804570 | RA | stroke | 2.39×10^-119^ |
| rs241436 | 6 | 32797876 | RA | stroke | 3.94×10^-82^ |
| rs2844509 | 6 | 31510924 | RA | stroke | 5.99×10^-80^ |
| rs1265096 | 6 | 31106489 | RA | stroke | 2.07×10^-76^ |
| rs2515919 | 6 | 31564167 | RA | stroke | 1.35×10^-74^ |
| rs1042663 | 6 | 31905130 | RA | stroke | 2.49×10^-73^ |
| rs9274623 | 6 | 32635998 | RA | stroke | 8.47×10^-72^ |
| rs2857107 | 6 | 32785515 | RA | stroke | 1.41×10^-69^ |
| rs3807039 | 6 | 32078373 | RA | stroke | 1.2×10^-66^ |
| rs453779 | 6 | 32975381 | RA | stroke | 1.98×10^-66^ |
| rs9267325 | 6 | 31461492 | RA | stroke | 4.24×10^-64^ |
| rs9378249 | 6 | 31327701 | RA | stroke | 1.41×10^-61^ |
| rs41267649 | 6 | 33384473 | RA | stroke | 2.62×10^-58^ |
| rs589428 | 6 | 31848220 | RA | stroke | 1.24×10^-54^ |
| rs3131004 | 6 | 31095294 | RA | stroke | 4.3×10^-50^ |
| rs2523615 | 6 | 31320642 | RA | stroke | 2×10^-49^ |
| rs4367411 | 6 | 32571929 | RA | stroke | 4.3×10^-47^ |
| rs28361065 | 6 | 32786291 | RA | stroke | 9.31×10^-46^ |
| rs12153855 | 6 | 32074804 | RA | stroke | 2.1×10^-44^ |
| rs481825 | 6 | 31780594 | RA | stroke | 2.48×10^-42^ |
| rs150360 | 6 | 32922078 | RA | stroke | 9.47×10^-42^ |
| rs9469556 | 6 | 33648661 | RA | stroke | 1.02×10^-40^ |
| rs41267086 | 6 | 31847636 | RA | stroke | 2.05×10^-40^ |
| rs9266075 | 6 | 31319754 | RA | stroke | 6.96×10^-39^ |
| rs17369215 | 6 | 29647715 | RA | stroke | 1.02×10^-37^ |
| rs6922431 | 6 | 31465030 | RA | stroke | 4.54×10^-34^ |
| rs2233981 | 6 | 31079578 | RA | stroke | 8.2×10^-34^ |
| rs4148876 | 6 | 32796793 | RA | stroke | 3.97×10^-33^ |
| rs2071025 | 6 | 33143756 | RA | stroke | 1.55×10^-32^ |
| rs2855812 | 6 | 31472720 | RA | stroke | 5.5×10^-32^ |
| rs1800838 | 6 | 33272541 | RA | stroke | 1.05×10^-31^ |
| rs72685677 | 1 | 113840826 | RA | stroke | 1.88×10^-31^ |
| rs241434 | 6 | 32798789 | RA | stroke | 4.19×10^-31^ |
| rs17201560 | 6 | 32047268 | RA | stroke | 2.97×10^-29^ |
| rs3129302 | 6 | 32974343 | RA | stroke | 1.09×10^-28^ |
| rs11102712 | 1 | 114537037 | RA | stroke | 2.9×10^-28^ |
| rs734181 | 6 | 33130208 | RA | stroke | 4.11×10^-27^ |
| rs41267090 | 6 | 31848440 | RA | stroke | 9.36×10^-27^ |
| rs2596496 | 6 | 31322782 | RA | stroke | 2.03×10^-26^ |
| rs9264232 | 6 | 31222243 | RA | stroke | 4.67×10^-26^ |
| rs1547669 | 6 | 33775641 | RA | stroke | 7.45×10^-26^ |
| rs12204421 | 6 | 33628863 | RA | stroke | 3.14×10^-25^ |
| rs7754218 | 6 | 33583446 | RA | stroke | 1.53×10^-23^ |
| rs3819301 | 6 | 31322229 | RA | stroke | 2.27×10^-23^ |
| rs9266193 | 6 | 31324788 | RA | stroke | 1.29×10^-22^ |
| rs10364 | 6 | 32780572 | RA | stroke | 3.97×10^-22^ |
| rs7731626 | 5 | 55444683 | RA | stroke | 1.71×10^-21^ |
| rs62391802 | 6 | 29695921 | RA | stroke | 1.68×10^-20^ |
| rs9263719 | 6 | 31096575 | RA | stroke | 1.38×10^-19^ |
| rs3087243 | 2 | 204738919 | RA | stroke | 1.82×10^-19^ |
| rs28397309 | 6 | 31234438 | RA | stroke | 2×10^-19^ |
| rs17207986 | 6 | 32079567 | RA | stroke | 2.42×10^-19^ |
| rs6922309 | 6 | 31321238 | RA | stroke | 3.25×10^-19^ |
| rs28711386 | 6 | 33132365 | RA | stroke | 1.85×10^-18^ |
| rs17264332 | 6 | 138005515 | RA | stroke | 2.02×10^-18^ |
| rs11751928 | 6 | 28335378 | RA | stroke | 2.24×10^-18^ |
| rs1217195 | 1 | 114178764 | RA | stroke | 8.55×10^-18^ |
| rs991760 | 6 | 32823567 | RA | stroke | 1.21×10^-17^ |
| rs17583244 | 6 | 32804687 | RA | stroke | 1.42×10^-17^ |
| rs10774624 | 12 | 111833788 | RA | stroke | 4.58×10^-17^ |
| rs61117627 | 6 | 138243700 | RA | stroke | 5.76×10^-17^ |
| rs17374222 | 15 | 69995344 | RA | stroke | 1.18×10^-16^ |
| rs34046593 | 4 | 26111593 | RA | stroke | 2.63×10^-16^ |
| rs72838643 | 6 | 29843390 | RA | stroke | 2.69×10^-16^ |
| rs34354079 | 6 | 32981555 | RA | stroke | 3.86×10^-16^ |
| rs68191 | 6 | 33480738 | RA | stroke | 4.26×10^-16^ |
| rs36097221 | 6 | 31847413 | RA | stroke | 5.56×10^-16^ |
| rs4810485 | 20 | 44747947 | RA | stroke | 6.38×10^-16^ |
| rs9267499 | 6 | 31537296 | RA | stroke | 8.72×10^-16^ |
| rs2235499 | 6 | 33129964 | RA | stroke | 1.25×10^-15^ |
| rs34536443 | 19 | 10463118 | RA | stroke | 1.34×10^-15^ |
| rs9368779 | 6 | 33816452 | RA | stroke | 5.17×10^-15^ |
| rs773588 | 1 | 113844824 | RA | stroke | 5.75×10^-15^ |
| rs11066283 | 12 | 112840766 | RA | stroke | 8.22×10^-15^ |
| rs11967839 | 6 | 33648144 | RA | stroke | 1.72×10^-14^ |
| rs3093017 | 6 | 167541258 | RA | stroke | 3×10^-14^ |
| rs10790268 | 11 | 118729391 | RA | stroke | 3.61×10^-14^ |
| rs3128935 | 6 | 32972404 | RA | stroke | 4.55×10^-14^ |
| rs45473295 | 6 | 33406405 | RA | stroke | 5.29×10^-14^ |
| rs3132558 | 6 | 31105466 | RA | stroke | 8.57×10^-14^ |
| rs34695944 | 2 | 61124850 | RA | stroke | 1.3×10^-13^ |
| rs28635450 | 6 | 32592633 | RA | stroke | 1.34×10^-13^ |
| rs4713711 | 6 | 33846143 | RA | stroke | 1.51×10^-13^ |
| rs45529339 | 6 | 31512936 | RA | stroke | 1.74×10^-13^ |
| rs2129977 | 4 | 111712432 | RA | stroke | 2.09×10^-13^ |
| rs55811970 | 1 | 114355237 | RA | stroke | 2.88×10^-13^ |
| rs420361 | 6 | 33555904 | RA | stroke | 6.29×10^-13^ |
| rs707937 | 6 | 31731014 | RA | stroke | 6.79×10^-13^ |
| rs3128852 | 6 | 29364135 | RA | stroke | 1.08×10^-12^ |
| rs72687965 | 1 | 114073152 | RA | stroke | 1.08×10^-12^ |
| rs71508903 | 10 | 63779871 | RA | stroke | 1.08×10^-12^ |
| rs1063478 | 6 | 32917544 | RA | stroke | 1.65×10^-12^ |
| rs2812378 | 9 | 34710260 | RA | stroke | 1.96×10^-12^ |
| rs7568275 | 2 | 191966452 | RA | stroke | 2.7×10^-12^ |
| rs61819425 | 1 | 114136618 | RA | stroke | 3.15×10^-12^ |
| rs42035 | 7 | 92239531 | RA | stroke | 4.23×10^-12^ |
| rs71393466 | 15 | 70034953 | RA | stroke | 5.42×10^-12^ |
| rs11587860 | 1 | 156156951 | RA | stroke | 5.87×10^-12^ |
| rs9653442 | 2 | 100825367 | RA | stroke | 1×10^-11^ |
| rs28703037 | 6 | 32628098 | RA | stroke | 1.15×10^-11^ |
| rs28724898 | 6 | 32803408 | RA | stroke | 1.25×10^-11^ |
| rs11968393 | 6 | 32779073 | RA | stroke | 1.29×10^-11^ |
| rs8043085 | 15 | 38828140 | RA | stroke | 1.32×10^-11^ |
| rs773566 | 1 | 113821584 | RA | stroke | 1.37×10^-11^ |
| rs706778 | 10 | 6098949 | RA | stroke | 2.02×10^-11^ |
| rs12539741 | 7 | 128596805 | RA | stroke | 3.3×10^-11^ |
| rs36229731 | 6 | 32593547 | RA | stroke | 3.44×10^-11^ |
| rs1050391 | 6 | 32917857 | RA | stroke | 5.28×10^-11^ |
| rs62405860 | 6 | 33459829 | RA | stroke | 8.63×10^-11^ |
| rs2107595 | 7 | 19049388 | RA | stroke | 9.09×10^-11^ |
| rs11085727 | 19 | 10466123 | RA | stroke | 1.84×10^-10^ |
| rs28635831 | 13 | 40319954 | RA | stroke | 2.11×10^-10^ |
| rs537544 | 10 | 8108382 | RA | stroke | 2.2×10^-10^ |
| rs6669008 | 1 | 114166561 | RA | stroke | 4.39×10^-10^ |
| rs2747429 | 6 | 29648377 | RA | stroke | 5.08×10^-10^ |
| rs7588874 | 2 | 204614508 | RA | stroke | 5.28×10^-10^ |
| rs2233959 | 6 | 31081065 | RA | stroke | 5.5×10^-10^ |
| rs4959130 | 6 | 1356916 | RA | stroke | 5.71×10^-10^ |
| rs41266809 | 6 | 26205209 | RA | stroke | 5.84×10^-10^ |
| rs4452313 | 3 | 17047032 | RA | stroke | 7.72×10^-10^ |
| rs2069235 | 22 | 39747780 | RA | stroke | 8.62×10^-10^ |
| rs2004640 | 7 | 128578301 | RA | stroke | 1.24×10^-9^ |
| rs3179004 | 6 | 31556922 | RA | stroke | 1.41×10^-9^ |
| rs9368744 | 6 | 32798299 | RA | stroke | 1.6×10^-9^ |
| rs1858037 | 2 | 65598300 | RA | stroke | 1.64×10^-9^ |
| rs2451258 | 6 | 159506600 | RA | stroke | 1.79×10^-9^ |
| rs9259913 | 6 | 29899653 | RA | stroke | 2.89×10^-9^ |
| rs10757278 | 9 | 22124477 | RA | stroke | 3.86×10^-9^ |
| rs7772638 | 6 | 29899756 | RA | stroke | 4.01×10^-9^ |
| rs2240336 | 1 | 17674402 | RA | stroke | 4.03×10^-9^ |
| rs10796038 | 10 | 6397964 | RA | stroke | 5.6×10^-9^ |
| rs17847933 | 6 | 33165780 | RA | stroke | 5.82×10^-9^ |
| rs2531830 | 6 | 28380832 | RA | stroke | 6.05×10^-9^ |
| rs547268 | 18 | 12823056 | RA | stroke | 6.19×10^-9^ |
| rs1736926 | 6 | 29692562 | RA | stroke | 7.39×10^-9^ |
| rs2561477 | 5 | 102608924 | RA | stroke | 7.73×10^-9^ |
| rs41555513 | 6 | 31323480 | RA | stroke | 9.9×10^-9^ |
| rs2872507 | 17 | 38040763 | RA | stroke | 1.02×10^-8^ |
| rs9394076 | 6 | 31522174 | RA | stroke | 1.14×10^-8^ |
| rs867435 | 1 | 2523706 | RA | stroke | 1.21×10^-8^ |
| rs28411352 | 1 | 38278579 | RA | stroke | 1.43×10^-8^ |
| rs1050388 | 6 | 31324506 | RA | stroke | 1.53×10^-8^ |
| rs1975161 | 19 | 10805160 | RA | stroke | 1.65×10^-8^ |
| rs72699046 | 1 | 113038761 | RA | stroke | 2.43×10^-8^ |
| rs62097857 | 18 | 12857758 | RA | stroke | 2.46×10^-8^ |
| rs13330176 | 16 | 86019087 | RA | stroke | 2.48×10^-8^ |
| rs210134 | 6 | 33540209 | RA | stroke | 2.53×10^-8^ |
| rs4942561 | 13 | 47209347 | RA | stroke | 2.59×10^-8^ |
| rs9405002 | 6 | 33131893 | RA | stroke | 3.67×10^-8^ |
| rs20547 | 6 | 32826233 | RA | stroke | 3.71×10^-8^ |
| rs10985070 | 9 | 123636121 | RA | stroke | 4.67×10^-8^ |
| rs225433 | 21 | 43809418 | RA | stroke | 4.88×10^-8^ |
| rs17042059 | 4 | 111641186 | RA | AF | 0 |
| rs1004095 | 6 | 32153409 | RA | AF | 0 |
| rs9368714 | 6 | 32297341 | RA | AF | 0 |
| rs2281276 | 6 | 32340106 | RA | AF | 0 |
| rs9275183 | 6 | 32654502 | RA | AF | 0 |
| rs3134976 | 6 | 32652305 | RA | AF | 1.73×10^-256^ |
| rs17219281 | 6 | 32675645 | RA | AF | 2.87×10^-238^ |
| rs8192574 | 6 | 32169145 | RA | AF | 7.56×10^-236^ |
| rs9267919 | 6 | 32206103 | RA | AF | 6.15×10^-229^ |
| rs35502919 | 6 | 31604355 | RA | AF | 1.44×10^-185^ |
| rs2261033 | 6 | 31603591 | RA | AF | 2.56×10^-181^ |
| rs35366682 | 6 | 32594354 | RA | AF | 3.73×10^-175^ |
| rs28752520 | 6 | 32584739 | RA | AF | 3.39×10^-166^ |
| rs2476601 | 1 | 114377568 | RA | AF | 4.98×10^-149^ |
| rs57912571 | 6 | 32750921 | RA | AF | 5.81×10^-148^ |
| rs9275698 | 6 | 32687973 | RA | AF | 3.37×10^-139^ |
| rs3135364 | 6 | 32389545 | RA | AF | 8.64×10^-136^ |
| rs3819720 | 6 | 32804570 | RA | AF | 3.29×10^-121^ |
| rs34492353 | 6 | 31520658 | RA | AF | 2.42×10^-119^ |
| rs7767732 | 6 | 32853277 | RA | AF | 3.93×10^-116^ |
| rs3128947 | 6 | 32965062 | RA | AF | 5.98×10^-115^ |
| rs6838973 | 4 | 111765495 | RA | AF | 1.39×10^-112^ |
| rs9267806 | 6 | 32110886 | RA | AF | 2.67×10^-107^ |
| rs440841 | 6 | 33019643 | RA | AF | 1.41×10^-105^ |
| rs3873444 | 6 | 32682724 | RA | AF | 1.89×10^-102^ |
| rs2040406 | 6 | 32603007 | RA | AF | 9.15×10^-102^ |
| rs35590025 | 6 | 30992111 | RA | AF | 2.76×10^-101^ |
| rs9501626 | 6 | 32400344 | RA | AF | 9.82×10^-97^ |
| rs2856822 | 6 | 33047432 | RA | AF | 3.35×10^-96^ |
| rs1704996 | 6 | 33182895 | RA | AF | 2×10^-95^ |
| rs2359171 | 16 | 73053022 | RA | AF | 2.44×10^-90^ |
| rs62407970 | 6 | 32936994 | RA | AF | 4.89×10^-86^ |
| rs2859094 | 6 | 32700233 | RA | AF | 3.48×10^-84^ |
| rs6457681 | 6 | 32773497 | RA | AF | 5.76×10^-83^ |
| rs2595117 | 4 | 111595516 | RA | AF | 1.8×10^-82^ |
| rs2844509 | 6 | 31510924 | RA | AF | 1.74×10^-81^ |
| rs11264280 | 1 | 154862952 | RA | AF | 8.75×10^-80^ |
| rs7754520 | 6 | 31906505 | RA | AF | 7.15×10^-75^ |
| rs3095255 | 6 | 31221581 | RA | AF | 1.58×10^-74^ |
| rs2523454 | 6 | 31367865 | RA | AF | 2.31×10^-74^ |
| rs9276831 | 6 | 32832033 | RA | AF | 2.46×10^-71^ |
| rs71205203 | 6 | 32667438 | RA | AF | 1.22×10^-67^ |
| rs9274408 | 6 | 32632850 | RA | AF | 3.93×10^-64^ |
| rs11598047 | 10 | 105342672 | RA | AF | 8.46×10^-64^ |
| rs1265093 | 6 | 31107187 | RA | AF | 3.68×10^-62^ |
| rs154977 | 6 | 32900018 | RA | AF | 5.32×10^-62^ |
| rs58110932 | 6 | 32371292 | RA | AF | 2.97×10^-61^ |
| rs17576984 | 6 | 32212985 | RA | AF | 8.64×10^-60^ |
| rs9357094 | 6 | 30167476 | RA | AF | 1.32×10^-59^ |
| rs73729070 | 6 | 32199462 | RA | AF | 4.97×10^-59^ |
| rs41267649 | 6 | 33384473 | RA | AF | 2.62×10^-58^ |
| rs7683219 | 4 | 111730447 | RA | AF | 3.75×10^-57^ |
| rs1071649 | 6 | 31239114 | RA | AF | 2.92×10^-56^ |
| rs11773845 | 7 | 116191301 | RA | AF | 3.59×10^-54^ |
| rs72700114 | 1 | 170193825 | RA | AF | 5.07×10^-54^ |
| rs3128948 | 6 | 32965085 | RA | AF | 5.17×10^-54^ |
| rs9276757 | 6 | 32772856 | RA | AF | 1.99×10^-53^ |
| rs8192585 | 6 | 32188823 | RA | AF | 3.5×10^-53^ |
| rs2524084 | 6 | 31241639 | RA | AF | 1.25×10^-52^ |
| rs9275601 | 6 | 32682664 | RA | AF | 5.99×10^-50^ |
| rs35855550 | 6 | 32418722 | RA | AF | 1.1×10^-49^ |
| rs9268909 | 6 | 32432340 | RA | AF | 3.99×10^-49^ |
| rs9267659 | 6 | 31846234 | RA | AF | 2.28×10^-45^ |
| rs9391736 | 6 | 32196656 | RA | AF | 1.12×10^-44^ |
| rs707932 | 6 | 31738343 | RA | AF | 3.28×10^-44^ |
| rs35176054 | 10 | 105480387 | RA | AF | 1.65×10^-43^ |
| rs2844698 | 6 | 30931245 | RA | AF | 4.15×10^-43^ |
| rs651386 | 1 | 170591310 | RA | AF | 8.84×10^-43^ |
| rs396090 | 6 | 32977535 | RA | AF | 9.47×10^-43^ |
| rs9277229 | 6 | 33027553 | RA | AF | 1.43×10^-42^ |
| rs11752643 | 6 | 32669373 | RA | AF | 3.78×10^-42^ |
| rs206776 | 6 | 32953711 | RA | AF | 1.08×10^-41^ |
| rs9267516 | 6 | 31569901 | RA | AF | 1.12×10^-41^ |
| rs17208314 | 6 | 32255771 | RA | AF | 6.56×10^-41^ |
| rs41265828 | 6 | 30458322 | RA | AF | 7.17×10^-41^ |
| rs9469556 | 6 | 33648661 | RA | AF | 1.11×10^-40^ |
| rs35149168 | 6 | 31635198 | RA | AF | 1.43×10^-40^ |
| rs41267086 | 6 | 31847636 | RA | AF | 1.23×10^-39^ |
| rs883079 | 12 | 114793240 | RA | AF | 1.23×10^-39^ |
| rs2256266 | 6 | 29632318 | RA | AF | 4.89×10^-39^ |
| rs28622052 | 6 | 32857086 | RA | AF | 3.83×10^-38^ |
| rs9276605 | 6 | 32735311 | RA | AF | 7.88×10^-38^ |
| rs11264273 | 1 | 154809253 | RA | AF | 8.53×10^-38^ |
| rs151719 | 6 | 32903900 | RA | AF | 3.38×10^-36^ |
| rs10800507 | 1 | 170185641 | RA | AF | 5.58×10^-36^ |
| rs13105878 | 4 | 111718147 | RA | AF | 5.65×10^-36^ |
| rs7172038 | 15 | 73667255 | RA | AF | 7.7×10^-36^ |
| rs3134928 | 6 | 32193564 | RA | AF | 2.04×10^-35^ |
| rs17171711 | 5 | 137364795 | RA | AF | 2.77×10^-35^ |
| rs6922431 | 6 | 31465030 | RA | AF | 3.82×10^-35^ |
| rs11098092 | 4 | 111798201 | RA | AF | 7.79×10^-35^ |
| rs187764 | 6 | 32902114 | RA | AF | 2.18×10^-34^ |
| rs7915134 | 10 | 75420180 | RA | AF | 2.89×10^-34^ |
| rs154973 | 6 | 32900642 | RA | AF | 8.14×10^-34^ |
| rs10821415 | 9 | 97713459 | RA | AF | 2.66×10^-33^ |
| rs17213554 | 6 | 32759694 | RA | AF | 5.43×10^-33^ |
| rs4434456 | 6 | 33101064 | RA | AF | 5.8×10^-33^ |
| rs6457718 | 6 | 33103827 | RA | AF | 3.91×10^-32^ |
| rs9405048 | 6 | 30670292 | RA | AF | 5.37×10^-32^ |
| rs9469079 | 6 | 32032421 | RA | AF | 6.46×10^-32^ |
| rs211453 | 6 | 33330131 | RA | AF | 6.76×10^-32^ |
| rs4124163 | 4 | 111745599 | RA | AF | 8.64×10^-32^ |
| rs72685677 | 1 | 113840826 | RA | AF | 1.87×10^-31^ |
| rs3097683 | 6 | 32965382 | RA | AF | 2.22×10^-31^ |
| rs3781370 | 10 | 105335156 | RA | AF | 2.83×10^-31^ |
| rs241434 | 6 | 32798789 | RA | AF | 4.17×10^-31^ |
| rs4711222 | 6 | 30533275 | RA | AF | 7.01×10^-31^ |
| rs2595082 | 4 | 111635292 | RA | AF | 7.93×10^-31^ |
| rs17615220 | 6 | 32677413 | RA | AF | 7.94×10^-31^ |
| rs3130183 | 6 | 33023996 | RA | AF | 8.18×10^-31^ |
| rs2738413 | 14 | 64679960 | RA | AF | 1.07×10^-30^ |
| rs12200756 | 6 | 33024258 | RA | AF | 2.98×10^-30^ |
| rs9262602 | 6 | 31020271 | RA | AF | 3.34×10^-30^ |
| rs7763502 | 6 | 30717637 | RA | AF | 6.49×10^-30^ |
| rs2596534 | 6 | 31445345 | RA | AF | 6.86×10^-30^ |
| rs9266635 | 6 | 31346979 | RA | AF | 1.1×10^-28^ |
| rs3095150 | 6 | 30932532 | RA | AF | 1.71×10^-28^ |
| rs1594470 | 6 | 32991987 | RA | AF | 2.19×10^-28^ |
| rs2244579 | 6 | 31436639 | RA | AF | 3.18×10^-28^ |
| rs1547670 | 6 | 33775868 | RA | AF | 1.37×10^-27^ |
| rs2857102 | 6 | 32793770 | RA | AF | 1.37×10^-27^ |
| rs12029644 | 1 | 114536780 | RA | AF | 1.55×10^-27^ |
| rs9277726 | 6 | 33091613 | RA | AF | 1.04×10^-26^ |
| rs2530696 | 6 | 30976496 | RA | AF | 1.86×10^-26^ |
| rs73402255 | 6 | 31397805 | RA | AF | 2.39×10^-26^ |
| rs41267090 | 6 | 31848440 | RA | AF | 4.3×10^-26^ |
| rs2517514 | 6 | 31029401 | RA | AF | 7.82×10^-26^ |
| rs2107202 | 6 | 30105743 | RA | AF | 9.77×10^-26^ |
| rs2878411 | 1 | 154823545 | RA | AF | 1.25×10^-25^ |
| rs62339024 | 4 | 111596577 | RA | AF | 4.01×10^-25^ |
| rs73366713 | 6 | 16415751 | RA | AF | 4.13×10^-25^ |
| rs10842383 | 12 | 24771967 | RA | AF | 7.33×10^-25^ |
| rs12204421 | 6 | 33628863 | RA | AF | 7.78×10^-25^ |
| rs3855819 | 4 | 111727163 | RA | AF | 1.46×10^-24^ |
| rs2288327 | 2 | 179411665 | RA | AF | 1.89×10^-24^ |
| rs7754218 | 6 | 33583446 | RA | AF | 3.31×10^-24^ |
| rs4642101 | 3 | 12842223 | RA | AF | 4.21×10^-24^ |
| rs9468976 | 6 | 31320473 | RA | AF | 5.39×10^-24^ |
| rs9267426 | 6 | 31476363 | RA | AF | 1.41×10^-23^ |
| rs56326533 | 2 | 201168758 | RA | AF | 2.13×10^-23^ |
| rs1131170 | 6 | 31324705 | RA | AF | 7.54×10^-23^ |
| rs876727 | 16 | 73067761 | RA | AF | 9.27×10^-23^ |
| rs3892710 | 6 | 32682862 | RA | AF | 1.02×10^-22^ |
| rs3749946 | 6 | 31448862 | RA | AF | 1.18×10^-22^ |
| rs9262661 | 6 | 31034592 | RA | AF | 1.25×10^-22^ |
| rs35647252 | 6 | 31881242 | RA | AF | 1.58×10^-22^ |
| rs35787014 | 6 | 29536498 | RA | AF | 3.43×10^-22^ |
| rs7731626 | 5 | 55444683 | RA | AF | 5.8×10^-22^ |
| rs4360170 | 6 | 31430359 | RA | AF | 1.66×10^-21^ |
| rs13149878 | 4 | 111863148 | RA | AF | 3.45×10^-21^ |
| rs3135022 | 6 | 33045966 | RA | AF | 3.92×10^-21^ |
| rs6882776 | 5 | 172664163 | RA | AF | 4.5×10^-21^ |
| rs9481842 | 6 | 118974798 | RA | AF | 5.77×10^-21^ |
| rs7508 | 8 | 17913970 | RA | AF | 7.45×10^-21^ |
| rs4757877 | 11 | 20010291 | RA | AF | 8.04×10^-21^ |
| rs17179851 | 6 | 29924440 | RA | AF | 9.63×10^-21^ |
| rs2395301 | 6 | 32968693 | RA | AF | 1.11×10^-20^ |
| rs2540949 | 2 | 65284231 | RA | AF | 1.52×10^-20^ |
| rs2291437 | 12 | 24715048 | RA | AF | 8.05×10^-20^ |
| rs6790396 | 3 | 38771925 | RA | AF | 8.63×10^-20^ |
| rs17513772 | 4 | 111884010 | RA | AF | 9.48×10^-20^ |
| rs10213638 | 4 | 111488510 | RA | AF | 1.94×10^-19^ |
| rs11156751 | 14 | 32990437 | RA | AF | 2.1×10^-19^ |
| rs4730751 | 7 | 116180850 | RA | AF | 2.26×10^-19^ |
| rs6922309 | 6 | 31321238 | RA | AF | 2.27×10^-19^ |
| rs9930504 | 16 | 73087689 | RA | AF | 2.29×10^-19^ |
| rs3087243 | 2 | 204738919 | RA | AF | 2.63×10^-19^ |
| rs17746631 | 4 | 111586444 | RA | AF | 2.85×10^-19^ |
| rs10753933 | 1 | 203026214 | RA | AF | 2.9×10^-19^ |
| rs3781339 | 10 | 105428152 | RA | AF | 3.84×10^-19^ |
| rs13201129 | 6 | 30601067 | RA | AF | 4.55×10^-19^ |
| rs12211782 | 6 | 30984878 | RA | AF | 4.9×10^-19^ |
| rs17207986 | 6 | 32079567 | RA | AF | 9.93×10^-19^ |
| rs17264332 | 6 | 138005515 | RA | AF | 1.99×10^-18^ |
| rs72966339 | 6 | 122398241 | RA | AF | 2.11×10^-18^ |
| rs11751928 | 6 | 28335378 | RA | AF | 2.21×10^-18^ |
| rs41316606 | 6 | 28862516 | RA | AF | 2.41×10^-18^ |
| rs9261819 | 6 | 30378989 | RA | AF | 2.46×10^-18^ |
| rs3765618 | 11 | 128769876 | RA | AF | 3.32×10^-18^ |
| rs10081039 | 6 | 31310056 | RA | AF | 3.45×10^-18^ |
| rs17337621 | 8 | 124542519 | RA | AF | 3.78×10^-18^ |
| rs35085781 | 6 | 33015421 | RA | AF | 5.45×10^-18^ |
| rs34969716 | 6 | 18210109 | RA | AF | 6.84×10^-18^ |
| rs9277768 | 6 | 33098569 | RA | AF | 8.42×10^-18^ |
| rs1217195 | 1 | 114178764 | RA | AF | 8.44×10^-18^ |
| rs72718146 | 1 | 170173443 | RA | AF | 8.71×10^-18^ |
| rs12527959 | 6 | 29537426 | RA | AF | 1.05×10^-17^ |
| rs12191464 | 6 | 33227518 | RA | AF | 1.64×10^-17^ |
| rs4999127 | 1 | 154714006 | RA | AF | 3.29×10^-17^ |
| rs72804710 | 5 | 142880532 | RA | AF | 4.49×10^-17^ |
| rs61117627 | 6 | 138243700 | RA | AF | 4.74×10^-17^ |
| rs11655198 | 17 | 38026169 | RA | AF | 5.31×10^-17^ |
| rs6888113 | 5 | 137012487 | RA | AF | 5.48×10^-17^ |
| rs8026898 | 15 | 69991417 | RA | AF | 6.79×10^-17^ |
| rs35215597 | 2 | 175543782 | RA | AF | 9.04×10^-17^ |
| rs2834618 | 21 | 36119111 | RA | AF | 1.01×10^-16^ |
| rs4713424 | 6 | 31002742 | RA | AF | 1.36×10^-16^ |
| rs34087996 | 6 | 31329062 | RA | AF | 1.52×10^-16^ |
| rs9262562 | 6 | 31008949 | RA | AF | 1.54×10^-16^ |
| rs210194 | 6 | 33482146 | RA | AF | 2.07×10^-16^ |
| rs11598294 | 10 | 105395501 | RA | AF | 2.54×10^-16^ |
| rs7804722 | 7 | 92288106 | RA | AF | 3.46×10^-16^ |
| rs9295939 | 6 | 30953968 | RA | AF | 4.52×10^-16^ |
| rs2240331 | 5 | 137780368 | RA | AF | 4.95×10^-16^ |
| rs73400019 | 6 | 33034166 | RA | AF | 6.31×10^-16^ |
| rs4387287 | 10 | 105677897 | RA | AF | 7.4×10^-16^ |
| rs41267134 | 6 | 32015793 | RA | AF | 1.05×10^-15^ |
| rs17220143 | 6 | 32787369 | RA | AF | 1.2×10^-15^ |
| rs34536443 | 19 | 10463118 | RA | AF | 1.32×10^-15^ |
| rs6448432 | 4 | 26098810 | RA | AF | 1.46×10^-15^ |
| rs337705 | 5 | 113737062 | RA | AF | 1.48×10^-15^ |
| rs6747542 | 2 | 70106832 | RA | AF | 2×10^-15^ |
| rs7373065 | 3 | 38710315 | RA | AF | 2.12×10^-15^ |
| rs9277009 | 6 | 32992036 | RA | AF | 2.18×10^-15^ |
| rs773588 | 1 | 113844824 | RA | AF | 2.35×10^-15^ |
| rs4947324 | 6 | 31528130 | RA | AF | 2.78×10^-15^ |
| rs12234159 | 6 | 31424086 | RA | AF | 4.26×10^-15^ |
| rs10804493 | 3 | 111554426 | RA | AF | 4.49×10^-15^ |
| rs12908004 | 15 | 80676925 | RA | AF | 4.56×10^-15^ |
| rs11974466 | 7 | 116004461 | RA | AF | 6.94×10^-15^ |
| rs17404424 | 6 | 29275298 | RA | AF | 8.26×10^-15^ |
| rs6905389 | 6 | 30319930 | RA | AF | 1.02×10^-14^ |
| rs3131051 | 6 | 30760181 | RA | AF | 1.38×10^-14^ |
| rs1571878 | 6 | 167540842 | RA | AF | 1.4×10^-14^ |
| rs9266628 | 6 | 31346811 | RA | AF | 2.04×10^-14^ |
| rs10046213 | 6 | 33704194 | RA | AF | 2.05×10^-14^ |
| rs6933672 | 6 | 28941688 | RA | AF | 2.07×10^-14^ |
| rs17401682 | 5 | 142896049 | RA | AF | 2.29×10^-14^ |
| rs6904550 | 6 | 149353758 | RA | AF | 3.95×10^-14^ |
| rs9368779 | 6 | 33816452 | RA | AF | 4.16×10^-14^ |
| rs72692270 | 1 | 51571017 | RA | AF | 4.21×10^-14^ |
| rs4711363 | 6 | 33852948 | RA | AF | 5.23×10^-14^ |
| rs11217060 | 11 | 118723748 | RA | AF | 5.91×10^-14^ |
| rs73241997 | 14 | 35173775 | RA | AF | 7.08×10^-14^ |
| rs13196770 | 6 | 32965363 | RA | AF | 8.11×10^-14^ |
| rs72694603 | 1 | 112458893 | RA | AF | 8.48×10^-14^ |
| rs707937 | 6 | 31731014 | RA | AF | 1.21×10^-13^ |
| rs3753639 | 1 | 154986091 | RA | AF | 1.32×10^-13^ |
| rs4239702 | 20 | 44749251 | RA | AF | 1.48×10^-13^ |
| rs11761443 | 7 | 115988015 | RA | AF | 2.01×10^-13^ |
| rs1908628 | 1 | 147260568 | RA | AF | 2.65×10^-13^ |
| rs55811970 | 1 | 114355237 | RA | AF | 2.83×10^-13^ |
| rs6900139 | 6 | 32955389 | RA | AF | 3.48×10^-13^ |
| rs11574914 | 9 | 34710338 | RA | AF | 4.13×10^-13^ |
| rs55754224 | 4 | 114428714 | RA | AF | 4.25×10^-13^ |
| rs11265613 | 1 | 154418415 | RA | AF | 4.37×10^-13^ |
| rs71454237 | 12 | 70013415 | RA | AF | 4.44×10^-13^ |
| rs12665140 | 6 | 29898720 | RA | AF | 4.54×10^-13^ |
| rs3758576 | 10 | 105454115 | RA | AF | 4.61×10^-13^ |
| rs3176326 | 6 | 36647289 | RA | AF | 5.01×10^-13^ |
| rs3094722 | 6 | 30774220 | RA | AF | 6.47×10^-13^ |
| rs12809354 | 12 | 32978437 | RA | AF | 7.01×10^-13^ |
| rs57321823 | 6 | 118620291 | RA | AF | 7.29×10^-13^ |
| rs17207895 | 6 | 32020512 | RA | AF | 7.5×10^-13^ |
| rs3853444 | 4 | 111734136 | RA | AF | 7.67×10^-13^ |
| rs34695944 | 2 | 61124850 | RA | AF | 7.8×10^-13^ |
| rs9261418 | 6 | 30076660 | RA | AF | 8.21×10^-13^ |
| rs297006 | 4 | 112006917 | RA | AF | 8.37×10^-13^ |
| rs1957021 | 14 | 32924505 | RA | AF | 8.89×10^-13^ |
| rs61819426 | 1 | 114137902 | RA | AF | 9.87×10^-13^ |
| rs72674110 | 4 | 112093543 | RA | AF | 9.98×10^-13^ |
| rs72687965 | 1 | 114073152 | RA | AF | 1.06×10^-12^ |
| rs71508903 | 10 | 63779871 | RA | AF | 1.06×10^-12^ |
| rs2066754 | 6 | 32945830 | RA | AF | 1.06×10^-12^ |
| rs55712499 | 4 | 174643983 | RA | AF | 1.3×10^-12^ |
| rs28705758 | 4 | 111445696 | RA | AF | 1.35×10^-12^ |
| rs13126426 | 4 | 111960374 | RA | AF | 1.36×10^-12^ |
| rs7453914 | 6 | 118692981 | RA | AF | 1.47×10^-12^ |
| rs72690473 | 1 | 51067819 | RA | AF | 1.76×10^-12^ |
| rs4073778 | 1 | 116297758 | RA | AF | 2.04×10^-12^ |
| rs7578393 | 2 | 26165528 | RA | AF | 2.15×10^-12^ |
| rs7898224 | 10 | 105516650 | RA | AF | 2.43×10^-12^ |
| rs73432769 | 6 | 30716170 | RA | AF | 2.81×10^-12^ |
| rs2860482 | 12 | 57105938 | RA | AF | 3.37×10^-12^ |
| rs71411040 | 15 | 70001013 | RA | AF | 3.91×10^-12^ |
| rs12730906 | 1 | 155414768 | RA | AF | 6.15×10^-12^ |
| rs6680785 | 1 | 170560332 | RA | AF | 6.17×10^-12^ |
| rs7900632 | 10 | 105559689 | RA | AF | 6.39×10^-12^ |
| rs12245149 | 10 | 65321147 | RA | AF | 6.43×10^-12^ |
| rs13426947 | 2 | 191933254 | RA | AF | 6.74×10^-12^ |
| rs8032939 | 15 | 38834033 | RA | AF | 6.75×10^-12^ |
| rs1563304 | 17 | 44874453 | RA | AF | 7.15×10^-12^ |
| rs11575848 | 6 | 31686943 | RA | AF | 8.34×10^-12^ |
| rs3781295 | 10 | 104140602 | RA | AF | 8.43×10^-12^ |
| rs17228353 | 5 | 137543572 | RA | AF | 9.98×10^-12^ |
| rs55947985 | 4 | 111899282 | RA | AF | 1.03×10^-11^ |
| rs3135460 | 6 | 32996773 | RA | AF | 1.05×10^-11^ |
| rs57165260 | 6 | 31836202 | RA | AF | 1.15×10^-11^ |
| rs11968393 | 6 | 32779073 | RA | AF | 1.32×10^-11^ |
| rs773566 | 1 | 113821584 | RA | AF | 1.34×10^-11^ |
| rs522573 | 3 | 111681549 | RA | AF | 1.65×10^-11^ |
| rs1247933 | 12 | 114692036 | RA | AF | 1.65×10^-11^ |
| rs9260918 | 6 | 29948751 | RA | AF | 1.74×10^-11^ |
| rs464901 | 22 | 18597502 | RA | AF | 1.87×10^-11^ |
| rs12203746 | 6 | 33728651 | RA | AF | 1.95×10^-11^ |
| rs706778 | 10 | 6098949 | RA | AF | 1.97×10^-11^ |
| rs17213728 | 6 | 32781954 | RA | AF | 2.02×10^-11^ |
| rs56297443 | 6 | 32832037 | RA | AF | 2.25×10^-11^ |
| rs56180201 | 5 | 173392960 | RA | AF | 2.37×10^-11^ |
| rs12539741 | 7 | 128596805 | RA | AF | 3.23×10^-11^ |
| rs2031522 | 6 | 87821501 | RA | AF | 4.01×10^-11^ |
| rs9653442 | 2 | 100825367 | RA | AF | 4.02×10^-11^ |
| rs12210733 | 6 | 118653075 | RA | AF | 4.89×10^-11^ |
| rs7789146 | 7 | 150661409 | RA | AF | 5.29×10^-11^ |
| rs55734480 | 7 | 14372009 | RA | AF | 5.52×10^-11^ |
| rs56193825 | 1 | 170371152 | RA | AF | 5.54×10^-11^ |
| rs41266809 | 6 | 26205209 | RA | AF | 6.87×10^-11^ |
| rs9401451 | 6 | 122099152 | RA | AF | 7.39×10^-11^ |
| rs17380837 | 12 | 26345526 | RA | AF | 7.62×10^-11^ |
| rs739703 | 5 | 136738924 | RA | AF | 7.97×10^-11^ |
| rs3796903 | 4 | 111538382 | RA | AF | 8.54×10^-11^ |
| rs28359997 | 6 | 29717911 | RA | AF | 8.79×10^-11^ |
| rs61811879 | 1 | 154849661 | RA | AF | 1.04×10^-10^ |
| rs9953366 | 18 | 46474192 | RA | AF | 1.2×10^-10^ |
| rs35544454 | 2 | 213266003 | RA | AF | 1.22×10^-10^ |
| rs6948592 | 7 | 28408309 | RA | AF | 1.27×10^-10^ |
| rs1411465 | 1 | 170097894 | RA | AF | 1.37×10^-10^ |
| rs10883939 | 10 | 105649572 | RA | AF | 1.45×10^-10^ |
| rs2535240 | 6 | 29643877 | RA | AF | 1.45×10^-10^ |
| rs35801 | 5 | 102608213 | RA | AF | 1.45×10^-10^ |
| rs6931228 | 6 | 29359170 | RA | AF | 1.5×10^-10^ |
| rs28387148 | 2 | 127433465 | RA | AF | 1.51×10^-10^ |
| rs10213171 | 4 | 148937537 | RA | AF | 1.64×10^-10^ |
| rs12023499 | 1 | 155031376 | RA | AF | 1.67×10^-10^ |
| rs11085727 | 19 | 10466123 | RA | AF | 1.8×10^-10^ |
| rs12663679 | 6 | 28990049 | RA | AF | 1.88×10^-10^ |
| rs2308622 | 6 | 31238029 | RA | AF | 1.95×10^-10^ |
| rs72811294 | 17 | 12618680 | RA | AF | 1.96×10^-10^ |
| rs537544 | 10 | 8108382 | RA | AF | 2.15×10^-10^ |
| rs34080181 | 3 | 66454191 | RA | AF | 2.4×10^-10^ |
| rs56144236 | 6 | 32720219 | RA | AF | 2.43×10^-10^ |
| rs6771054 | 3 | 89489529 | RA | AF | 2.51×10^-10^ |
| rs58667488 | 6 | 32431785 | RA | AF | 3.02×10^-10^ |
| rs11751198 | 6 | 31753526 | RA | AF | 3.89×10^-10^ |
| rs2274115 | 9 | 139094773 | RA | AF | 4.05×10^-10^ |
| rs4965430 | 15 | 99268850 | RA | AF | 4.51×10^-10^ |
| rs3179004 | 6 | 31556922 | RA | AF | 4.73×10^-10^ |
| rs72926475 | 2 | 86594487 | RA | AF | 5.23×10^-10^ |
| rs7753474 | 6 | 29341308 | RA | AF | 5.26×10^-10^ |
| rs28635831 | 13 | 40319954 | RA | AF | 5.33×10^-10^ |
| rs133902 | 22 | 26164079 | RA | AF | 5.89×10^-10^ |
| rs1264704 | 6 | 30065319 | RA | AF | 6.95×10^-10^ |
| rs10520260 | 4 | 174447349 | RA | AF | 7.08×10^-10^ |
| rs6669008 | 1 | 114166561 | RA | AF | 7.28×10^-10^ |
| rs4452313 | 3 | 17047032 | RA | AF | 7.54×10^-10^ |
| rs10796038 | 10 | 6397964 | RA | AF | 7.59×10^-10^ |
| rs35005436 | 7 | 74134911 | RA | AF | 7.66×10^-10^ |
| rs2069235 | 22 | 39747780 | RA | AF | 8.41×10^-10^ |
| rs4673266 | 2 | 204634569 | RA | AF | 8.78×10^-10^ |
| rs775439 | 12 | 70096374 | RA | AF | 9.45×10^-10^ |
| rs10919470 | 1 | 170762680 | RA | AF | 9.97×10^-10^ |
| rs2004640 | 7 | 128578301 | RA | AF | 1.21×10^-9^ |
| rs452850 | 6 | 29799481 | RA | AF | 1.3×10^-9^ |
| rs6818418 | 4 | 103890430 | RA | AF | 1.32×10^-9^ |
| rs7019540 | 9 | 97598150 | RA | AF | 1.43×10^-9^ |
| rs1248051 | 12 | 114854929 | RA | AF | 1.6×10^-9^ |
| rs1858037 | 2 | 65598300 | RA | AF | 1.61×10^-9^ |
| rs10458660 | 10 | 77936576 | RA | AF | 1.83×10^-9^ |
| rs17608766 | 17 | 45013271 | RA | AF | 2.04×10^-9^ |
| rs3782464 | 12 | 114804580 | RA | AF | 2.31×10^-9^ |
| rs62407562 | 6 | 33530346 | RA | AF | 2.4×10^-9^ |
| rs897393 | 12 | 123312051 | RA | AF | 2.48×10^-9^ |
| rs284277 | 1 | 10790797 | RA | AF | 2.6×10^-9^ |
| rs9368696 | 6 | 31554382 | RA | AF | 2.76×10^-9^ |
| rs17840113 | 6 | 32300740 | RA | AF | 2.97×10^-9^ |
| rs1979409 | 15 | 73465477 | RA | AF | 3.07×10^-9^ |
| rs17847933 | 6 | 33165780 | RA | AF | 3.15×10^-9^ |
| rs7834729 | 8 | 21821778 | RA | AF | 3.34×10^-9^ |
| rs4716067 | 6 | 16413669 | RA | AF | 3.45×10^-9^ |
| rs73041705 | 3 | 24463235 | RA | AF | 3.5×10^-9^ |
| rs17005647 | 3 | 69406181 | RA | AF | 3.5×10^-9^ |
| rs1218550 | 1 | 154801247 | RA | AF | 3.52×10^-9^ |
| rs8069451 | 17 | 37504933 | RA | AF | 3.55×10^-9^ |
| rs2240336 | 1 | 17674402 | RA | AF | 3.93×10^-9^ |
| rs6811162 | 4 | 111440808 | RA | AF | 4.03×10^-9^ |
| rs9899183 | 17 | 7452977 | RA | AF | 4.52×10^-9^ |
| rs7529220 | 1 | 22282619 | RA | AF | 4.53×10^-9^ |
| rs11589091 | 1 | 170708980 | RA | AF | 4.66×10^-9^ |
| rs12621647 | 2 | 145734946 | RA | AF | 4.71×10^-9^ |
| rs6994744 | 8 | 141740868 | RA | AF | 5.03×10^-9^ |
| rs365990 | 14 | 23861811 | RA | AF | 5.26×10^-9^ |
| rs2451258 | 6 | 159506600 | RA | AF | 5.37×10^-9^ |
| rs9506925 | 13 | 23368943 | RA | AF | 5.78×10^-9^ |
| rs2531830 | 6 | 28380832 | RA | AF | 5.9×10^-9^ |
| rs2853920 | 6 | 31267394 | RA | AF | 6.05×10^-9^ |
| rs524788 | 4 | 111467459 | RA | AF | 6.08×10^-09^ |
| rs6733446 | 2 | 61806999 | RA | AF | 6.19×10^-09^ |
| rs11102343 | 1 | 112382671 | RA | AF | 6.28×10^-09^ |
| rs9276964 | 6 | 32964967 | RA | AF | 6.43×10^-9^ |
| rs12188351 | 5 | 168386089 | RA | AF | 6.58×10^-9^ |
| rs2759301 | 15 | 80994288 | RA | AF | 6.95×10^-9^ |
| rs2885697 | 1 | 41544279 | RA | AF | 7.35×10^-9^ |
| rs10752747 | 1 | 2524915 | RA | AF | 7.56×10^-9^ |
| rs6596717 | 5 | 106427609 | RA | AF | 7.62×10^-9^ |
| rs2815301 | 16 | 2004718 | RA | AF | 8.19×10^-9^ |
| rs62407980 | 6 | 33013458 | RA | AF | 8.43×10^-9^ |
| rs28986304 | 6 | 29566082 | RA | AF | 8.49×10^-9^ |
| rs1374471 | 10 | 103659601 | RA | AF | 8.49×10^-9^ |
| rs2156664 | 11 | 121645005 | RA | AF | 8.98×10^-9^ |
| rs6890182 | 5 | 172600288 | RA | AF | 9.11×10^-9^ |
| rs5752235 | 22 | 26249277 | RA | AF | 9.44×10^-9^ |
| rs182424 | 2 | 201103706 | RA | AF | 9.56×10^-9^ |
| rs2033570 | 2 | 198952637 | RA | AF | 9.99×10^-9^ |
| rs1035407 | 5 | 128181345 | RA | AF | 1.01×10^-8^ |
| rs592390 | 18 | 12822314 | RA | AF | 1.01×10^-8^ |
| rs5024505 | 4 | 112204259 | RA | AF | 1.09×10^-8^ |
| rs2071342 | 16 | 28915433 | RA | AF | 1.11×10^-8^ |
| rs2110552 | 14 | 73355131 | RA | AF | 1.14×10^-8^ |
| rs7612445 | 3 | 179172979 | RA | AF | 1.18×10^-8^ |
| rs899268 | 9 | 97799129 | RA | AF | 1.27×10^-8^ |
| rs7088041 | 10 | 105297976 | RA | AF | 1.34×10^-8^ |
| rs11203203 | 21 | 43836186 | RA | AF | 1.34×10^-8^ |
| rs210636 | 6 | 117884738 | RA | AF | 1.36×10^-8^ |
| rs12426679 | 12 | 76237987 | RA | AF | 1.44×10^-8^ |
| rs1125753 | 9 | 98103576 | RA | AF | 1.44×10^-8^ |
| rs8007348 | 14 | 73272730 | RA | AF | 1.46×10^-8^ |
| rs4076057 | 5 | 114386094 | RA | AF | 1.56×10^-8^ |
| rs13213216 | 6 | 31327738 | RA | AF | 1.58×10^-8^ |
| rs8133843 | 21 | 36738242 | RA | AF | 1.61×10^-8^ |
| rs2105325 | 1 | 173349725 | RA | AF | 1.61×10^-8^ |
| rs10873298 | 14 | 77426525 | RA | AF | 1.66×10^-8^ |
| rs11758136 | 6 | 29764647 | RA | AF | 1.71×10^-8^ |
| rs527888 | 3 | 136031358 | RA | AF | 1.8×10^-8^ |
| rs921153 | 2 | 61563408 | RA | AF | 1.9×10^-8^ |
| rs55985730 | 7 | 128417044 | RA | AF | 2.32×10^-8^ |
| rs2231478 | 5 | 137527917 | RA | AF | 2.35×10^-8^ |
| rs7225165 | 17 | 1309850 | RA | AF | 2.36×10^-8^ |
| rs62097857 | 18 | 12857758 | RA | AF | 2.39×10^-8^ |
| rs13330176 | 16 | 86019087 | RA | AF | 2.41×10^-8^ |
| rs61826205 | 1 | 170559626 | RA | AF | 2.52×10^-8^ |
| rs1918786 | 17 | 44279101 | RA | AF | 2.53×10^-8^ |
| rs617328 | 6 | 138000398 | RA | AF | 2.65×10^-8^ |
| rs11755295 | 6 | 119013402 | RA | AF | 3.21×10^-8^ |
| rs6926568 | 6 | 33597079 | RA | AF | 3.32×10^-8^ |
| rs6480769 | 10 | 76851503 | RA | AF | 3.36×10^-8^ |
| rs1458038 | 4 | 81164723 | RA | AF | 3.42×10^-8^ |
| rs2072412 | 7 | 150647970 | RA | AF | 3.78×10^-8^ |
| rs6665349 | 1 | 170789600 | RA | AF | 3.9×10^-8^ |
| rs13097780 | 3 | 38683021 | RA | AF | 3.99×10^-8^ |
| rs57718550 | 6 | 31978635 | RA | AF | 4.04×10^-8^ |
| rs12640611 | 4 | 10104478 | RA | AF | 4.13×10^-8^ |
| rs4414093 | 1 | 170185362 | RA | AF | 4.19×10^-8^ |
| rs10927010 | 1 | 243578812 | RA | AF | 4.22×10^-8^ |
| rs2297301 | 14 | 64644205 | RA | AF | 4.35×10^-8^ |
| rs10985070 | 9 | 123636121 | RA | AF | 4.55×10^-8^ |
| rs2613813 | 19 | 5174936 | RA | AF | 4.59×10^-8^ |
| rs12049646 | 10 | 69623984 | RA | AF | 4.66×10^-8^ |
| rs60902112 | 3 | 194800853 | RA | AF | 4.71×10^-8^ |
| rs1004095 | 6 | 32153409 | RA | HF | 0 |
| rs9267954 | 6 | 32213052 | RA | HF | 0 |
| rs2281276 | 6 | 32340106 | RA | HF | 0 |
| rs9268560 | 6 | 32389512 | RA | HF | 0 |
| rs9275183 | 6 | 32654502 | RA | HF | 0 |
| rs17219281 | 6 | 32675645 | RA | HF | 2.87×10^-238^ |
| rs41268928 | 6 | 32147157 | RA | HF | 6.6×10^-227^ |
| rs9275563 | 6 | 32677912 | RA | HF | 5.74×10^-220^ |
| rs35502919 | 6 | 31604355 | RA | HF | 1.44×10^-185^ |
| rs2261033 | 6 | 31603591 | RA | HF | 1.28×10^-180^ |
| rs35366682 | 6 | 32594354 | RA | HF | 7.41×10^-177^ |
| rs3132949 | 6 | 32195935 | RA | HF | 5.83×10^-166^ |
| rs2894381 | 6 | 32682228 | RA | HF | 6.16×10^-154^ |
| rs2476601 | 1 | 114377568 | RA | HF | 4.98×10^-149^ |
| rs57912571 | 6 | 32750921 | RA | HF | 1.48×10^-148^ |
| rs9275698 | 6 | 32687973 | RA | HF | 3.37×10^-139^ |
| rs3819720 | 6 | 32804570 | RA | HF | 1.22×10^-119^ |
| rs34492353 | 6 | 31520658 | RA | HF | 1.69×10^-119^ |
| rs7767732 | 6 | 32853277 | RA | HF | 4×10^-116^ |
| rs3128947 | 6 | 32965062 | RA | HF | 5.86×10^-114^ |
| rs9275553 | 6 | 32676988 | RA | HF | 6.4×10^-112^ |
| rs9267806 | 6 | 32110886 | RA | HF | 2.67×10^-107^ |
| rs440841 | 6 | 33019643 | RA | HF | 8.26×10^-106^ |
| rs35590025 | 6 | 30992111 | RA | HF | 2.76×10^-101^ |
| rs62404579 | 6 | 32376019 | RA | HF | 4.13×10^-100^ |
| rs2856822 | 6 | 33047432 | RA | HF | 3.35×10^-96^ |
| rs7775228 | 6 | 32658079 | RA | HF | 2.42×10^-95^ |
| rs1704996 | 6 | 33182895 | RA | HF | 8.8×10^-94^ |
| rs62407970 | 6 | 32936994 | RA | HF | 1.64×10^-84^ |
| rs2859094 | 6 | 32700233 | RA | HF | 3.48×10^-84^ |
| rs547921 | 6 | 31944320 | RA | HF | 3.73×10^-82^ |
| rs241436 | 6 | 32797876 | RA | HF | 3.94×10^-82^ |
| rs2844509 | 6 | 31510924 | RA | HF | 1.74×10^-81^ |
| rs3132489 | 6 | 31242674 | RA | HF | 2×10^-73^ |
| rs9276831 | 6 | 32832033 | RA | HF | 2.46×10^-71^ |
| rs3132963 | 6 | 32320153 | RA | HF | 7.88×10^-71^ |
| rs9267860 | 6 | 32196641 | RA | HF | 1.8×10^-62^ |
| rs154977 | 6 | 32900018 | RA | HF | 2.5×10^-62^ |
| rs9357094 | 6 | 30167476 | RA | HF | 1.32×10^-59^ |
| rs17576984 | 6 | 32212985 | RA | HF | 1.82×10^-58^ |
| rs41267649 | 6 | 33384473 | RA | HF | 3.99×10^-57^ |
| rs1480383 | 6 | 32740856 | RA | HF | 1.82×10^-54^ |
| rs3128948 | 6 | 32965085 | RA | HF | 5.17×10^-54^ |
| rs9264568 | 6 | 31235383 | RA | HF | 5.37×10^-53^ |
| rs3887152 | 6 | 31176335 | RA | HF | 6.85×10^-52^ |
| rs2428494 | 6 | 31322197 | RA | HF | 1.34×10^-51^ |
| rs8192585 | 6 | 32188823 | RA | HF | 1.66×10^-50^ |
| rs9275601 | 6 | 32682664 | RA | HF | 7.13×10^-50^ |
| rs9268909 | 6 | 32432340 | RA | HF | 3.99×10^-49^ |
| rs35795092 | 6 | 32151420 | RA | HF | 4.58×10^-48^ |
| rs204991 | 6 | 32161366 | RA | HF | 9.79×10^-47^ |
| rs4367411 | 6 | 32571929 | RA | HF | 4.96×10^-46^ |
| rs707932 | 6 | 31738343 | RA | HF | 3.29×10^-44^ |
| rs34673422 | 6 | 32332661 | RA | HF | 8.05×10^-44^ |
| rs2844698 | 6 | 30931245 | RA | HF | 4.16×10^-43^ |
| rs396090 | 6 | 32977535 | RA | HF | 9.1×10^-43^ |
| rs9267516 | 6 | 31569901 | RA | HF | 1.12×10^-41^ |
| rs206776 | 6 | 32953711 | RA | HF | 2.09×10^-41^ |
| rs9469556 | 6 | 33648661 | RA | HF | 4.39×10^-41^ |
| rs35149168 | 6 | 31635198 | RA | HF | 1.43×10^-40^ |
| rs41267086 | 6 | 31847636 | RA | HF | 2.16×10^-40^ |
| rs34976781 | 6 | 32587869 | RA | HF | 3.8×10^-39^ |
| rs2256266 | 6 | 29632318 | RA | HF | 4.9×10^-39^ |
| rs9262603 | 6 | 31020543 | RA | HF | 9.13×10^-39^ |
| rs28622052 | 6 | 32857086 | RA | HF | 3.84×10^-38^ |
| rs151719 | 6 | 32903900 | RA | HF | 8.86×10^-37^ |
| rs6922431 | 6 | 31465030 | RA | HF | 3.84×10^-35^ |
| rs9265169 | 6 | 31289272 | RA | HF | 4.37×10^-35^ |
| rs1264372 | 6 | 30769726 | RA | HF | 5.05×10^-35^ |
| rs187764 | 6 | 32902114 | RA | HF | 2.99×10^-34^ |
| rs2523484 | 6 | 31353639 | RA | HF | 6.68×10^-34^ |
| rs4148876 | 6 | 32796793 | RA | HF | 3.98×10^-33^ |
| rs2395490 | 6 | 31458993 | RA | HF | 5.12×10^-33^ |
| rs154973 | 6 | 32900642 | RA | HF | 5.23×10^-33^ |
| rs4434456 | 6 | 33101064 | RA | HF | 5.83×10^-33^ |
| rs9276931 | 6 | 32928984 | RA | HF | 8.89×10^-33^ |
| rs6457718 | 6 | 33103827 | RA | HF | 3.93×10^-32^ |
| rs3130183 | 6 | 33023996 | RA | HF | 9.01×10^-32^ |
| rs1800838 | 6 | 33272541 | RA | HF | 1.05×10^-31^ |
| rs72685677 | 1 | 113840826 | RA | HF | 1.88×10^-31^ |
| rs28744296 | 6 | 33440416 | RA | HF | 2.35×10^-31^ |
| rs4713470 | 6 | 31472821 | RA | HF | 3.91×10^-31^ |
| rs241434 | 6 | 32798789 | RA | HF | 4.2×10^-31^ |
| rs4711222 | 6 | 30533275 | RA | HF | 2.83×10^-30^ |
| rs7763502 | 6 | 30717637 | RA | HF | 9.2×10^-30^ |
| rs12200756 | 6 | 33024258 | RA | HF | 3.77×10^-29^ |
| rs2857102 | 6 | 32793770 | RA | HF | 2.43×10^-28^ |
| rs2244579 | 6 | 31436639 | RA | HF | 3.2×10^-28^ |
| rs9267573 | 6 | 31809866 | RA | HF | 4.23×10^-28^ |
| rs1632862 | 6 | 30969003 | RA | HF | 2.19×10^-27^ |
| rs12192933 | 6 | 32969390 | RA | HF | 2.31×10^-27^ |
| rs41267090 | 6 | 31848440 | RA | HF | 2.36×10^-27^ |
| rs1547669 | 6 | 33775641 | RA | HF | 4.91×10^-27^ |
| rs11102712 | 1 | 114537037 | RA | HF | 5×10^-27^ |
| rs9277726 | 6 | 33091613 | RA | HF | 1.05×10^-26^ |
| rs17219974 | 6 | 32762756 | RA | HF | 2.97×10^-26^ |
| rs12204421 | 6 | 33628863 | RA | HF | 4.61×10^-26^ |
| rs7739491 | 6 | 30979203 | RA | HF | 4.84×10^-26^ |
| rs2517514 | 6 | 31029401 | RA | HF | 7.89×10^-26^ |
| rs652888 | 6 | 31851234 | RA | HF | 1.38×10^-25^ |
| rs2107202 | 6 | 30105743 | RA | HF | 1.69×10^-24^ |
| rs2050189 | 6 | 32339647 | RA | HF | 2.29×10^-24^ |
| rs7754218 | 6 | 33583446 | RA | HF | 2.11×10^-23^ |
| rs3749946 | 6 | 31448862 | RA | HF | 1.2×10^-22^ |
| rs7731626 | 5 | 55444683 | RA | HF | 2.43×10^-22^ |
| rs35787014 | 6 | 29536498 | RA | HF | 3.48×10^-22^ |
| rs28367846 | 6 | 31309057 | RA | HF | 6.56×10^-22^ |
| rs6922309 | 6 | 31321238 | RA | HF | 1.07×10^-21^ |
| rs4360170 | 6 | 31430359 | RA | HF | 1.69×10^-21^ |
| rs9468873 | 6 | 31101880 | RA | HF | 6.62×10^-21^ |
| rs17179851 | 6 | 29924440 | RA | HF | 9.79×10^-21^ |
| rs3135022 | 6 | 33045966 | RA | HF | 6.49×10^-20^ |
| rs3087243 | 2 | 204738919 | RA | HF | 2.67×10^-19^ |
| rs13201129 | 6 | 30601067 | RA | HF | 3.55×10^-19^ |
| rs1906618 | 4 | 111695422 | RA | HF | 3.73×10^-19^ |
| rs11757201 | 6 | 138003822 | RA | HF | 1.54×10^-18^ |
| rs17207986 | 6 | 32079567 | RA | HF | 2.14×10^-18^ |
| rs11751928 | 6 | 28335378 | RA | HF | 2.26×10^-18^ |
| rs35085781 | 6 | 33015421 | RA | HF | 3.68×10^-18^ |
| rs9261819 | 6 | 30378989 | RA | HF | 7.59×10^-18^ |
| rs12527959 | 6 | 29537426 | RA | HF | 1.07×10^-17^ |
| rs41316606 | 6 | 28862516 | RA | HF | 1.76×10^-17^ |
| rs68191 | 6 | 33480738 | RA | HF | 3.53×10^-17^ |
| rs7752903 | 6 | 138227364 | RA | HF | 4.02×10^-17^ |
| rs8026898 | 15 | 69991417 | RA | HF | 6.95×10^-17^ |
| rs34287387 | 6 | 31807682 | RA | HF | 9.81×10^-17^ |
| rs1217195 | 1 | 114178764 | RA | HF | 1.05×10^-16^ |
| rs34087996 | 6 | 31329062 | RA | HF | 1.56×10^-16^ |
| rs9262562 | 6 | 31008949 | RA | HF | 1.57×10^-16^ |
| rs2736426 | 6 | 31745284 | RA | HF | 3.91×10^-16^ |
| rs9295939 | 6 | 30953968 | RA | HF | 4.64×10^-16^ |
| rs1537371 | 9 | 22099568 | RA | HF | 5.74×10^-16^ |
| rs73400019 | 6 | 33034166 | RA | HF | 6.47×10^-16^ |
| rs3130160 | 6 | 33124972 | RA | HF | 8.16×10^-16^ |
| rs4713424 | 6 | 31002742 | RA | HF | 9.84×10^-16^ |
| rs6448432 | 4 | 26098810 | RA | HF | 1.01×10^-15^ |
| rs34536443 | 19 | 10463118 | RA | HF | 1.36×10^-15^ |
| rs2856995 | 6 | 32783337 | RA | HF | 2.04×10^-15^ |
| rs773588 | 1 | 113844824 | RA | HF | 2.41×10^-15^ |
| rs17404424 | 6 | 29275298 | RA | HF | 3.08×10^-15^ |
| rs11967839 | 6 | 33648144 | RA | HF | 3.26×10^-15^ |
| rs11217044 | 11 | 118696022 | RA | HF | 5.9×10^-15^ |
| rs3132471 | 6 | 31419201 | RA | HF | 7.54×10^-15^ |
| rs6905389 | 6 | 30319930 | RA | HF | 1.05×10^-14^ |
| rs1571878 | 6 | 167540842 | RA | HF | 1.44×10^-14^ |
| rs9277009 | 6 | 32992036 | RA | HF | 1.99×10^-14^ |
| rs6933672 | 6 | 28941688 | RA | HF | 2.13×10^-14^ |
| rs7740525 | 6 | 30760698 | RA | HF | 3.48×10^-14^ |
| rs17200698 | 6 | 31483700 | RA | HF | 3.55×10^-14^ |
| rs45473295 | 6 | 33406405 | RA | HF | 3.66×10^-14^ |
| rs13214608 | 6 | 30068613 | RA | HF | 4.82×10^-14^ |
| rs9261408 | 6 | 30074749 | RA | HF | 5.92×10^-14^ |
| rs9368779 | 6 | 33816452 | RA | HF | 5.98×10^-14^ |
| rs2394898 | 6 | 31213583 | RA | HF | 7.76×10^-14^ |
| rs13196770 | 6 | 32965363 | RA | HF | 8.36×10^-14^ |
| rs4711363 | 6 | 33852948 | RA | HF | 1.68×10^-13^ |
| rs55811970 | 1 | 114355237 | RA | HF | 2.91×10^-13^ |
| rs6900139 | 6 | 32955389 | RA | HF | 3.6×10^-13^ |
| rs55963299 | 2 | 61097583 | RA | HF | 4.49×10^-13^ |
| rs4239702 | 20 | 44749251 | RA | HF | 5.41×10^-13^ |
| rs12764378 | 10 | 63800004 | RA | HF | 5.7×10^-13^ |
| rs3909134 | 6 | 29699253 | RA | HF | 6.9×10^-13^ |
| rs17207895 | 6 | 32020512 | RA | HF | 7.74×10^-13^ |
| rs11574914 | 9 | 34710338 | RA | HF | 9.92×10^-13^ |
| rs72687965 | 1 | 114073152 | RA | HF | 1.09×10^-12^ |
| rs10774624 | 12 | 111833788 | RA | HF | 1.55×10^-12^ |
| rs419261 | 6 | 33554147 | RA | HF | 1.98×10^-12^ |
| rs61819425 | 1 | 114136618 | RA | HF | 3.2×10^-12^ |
| rs3094722 | 6 | 30774220 | RA | HF | 3.3×10^-12^ |
| rs12525100 | 6 | 31673895 | RA | HF | 5.79×10^-12^ |
| rs73432769 | 6 | 30716170 | RA | HF | 6.22×10^-12^ |
| rs8032939 | 15 | 38834033 | RA | HF | 6.99×10^-12^ |
| rs3135460 | 6 | 32996773 | RA | HF | 7.13×10^-12^ |
| rs7162669 | 15 | 70022164 | RA | HF | 1.02×10^-11^ |
| rs6712515 | 2 | 100806514 | RA | HF | 1.37×10^-11^ |
| rs773566 | 1 | 113821584 | RA | HF | 1.39×10^-11^ |
| rs7568275 | 2 | 191966452 | RA | HF | 1.46×10^-11^ |
| rs11968393 | 6 | 32779073 | RA | HF | 1.49×10^-11^ |
| rs9260918 | 6 | 29948751 | RA | HF | 1.8×10^-11^ |
| rs706778 | 10 | 6098949 | RA | HF | 2.05×10^-11^ |
| rs9295986 | 6 | 31338528 | RA | HF | 2.06×10^-11^ |
| rs17213728 | 6 | 32781954 | RA | HF | 2.1×10^-11^ |
| rs56297443 | 6 | 32832037 | RA | HF | 2.34×10^-11^ |
| rs7763192 | 6 | 32338885 | RA | HF | 2.73×10^-11^ |
| rs12539741 | 7 | 128596805 | RA | HF | 3.35×10^-11^ |
| rs12663679 | 6 | 28990049 | RA | HF | 4.1×10^-11^ |
| rs2535240 | 6 | 29643877 | RA | HF | 4.89×10^-11^ |
| rs7746553 | 6 | 31895973 | RA | HF | 6.18×10^-11^ |
| rs9903250 | 17 | 38031030 | RA | HF | 6.9×10^-11^ |
| rs41266809 | 6 | 26205209 | RA | HF | 7.13×10^-11^ |
| rs28359997 | 6 | 29717911 | RA | HF | 9.14×10^-11^ |
| rs9276964 | 6 | 32964967 | RA | HF | 9.72×10^-11^ |
| rs6669008 | 1 | 114166561 | RA | HF | 1.25×10^-10^ |
| rs4673266 | 2 | 204634569 | RA | HF | 1.28×10^-10^ |
| rs55730499 | 6 | 161005610 | RA | HF | 1.41×10^-10^ |
| rs11085727 | 19 | 10466123 | RA | HF | 1.87×10^-10^ |
| rs452850 | 6 | 29799481 | RA | HF | 2.01×10^-10^ |
| rs28635831 | 13 | 40319954 | RA | HF | 2.14×10^-10^ |
| rs537544 | 10 | 8108382 | RA | HF | 2.24×10^-10^ |
| rs56144236 | 6 | 32720219 | RA | HF | 2.53×10^-10^ |
| rs58667488 | 6 | 32431785 | RA | HF | 3.43×10^-10^ |
| rs2517554 | 6 | 30993499 | RA | HF | 4.16×10^-10^ |
| rs3179004 | 6 | 31556922 | RA | HF | 4.93×10^-10^ |
| rs17696736 | 12 | 112486818 | RA | HF | 5.47×10^-10^ |
| rs7753474 | 6 | 29341308 | RA | HF | 5.48×10^-10^ |
| rs1264704 | 6 | 30065319 | RA | HF | 7.25×10^-10^ |
| rs4452313 | 3 | 17047032 | RA | HF | 7.86×10^-10^ |
| rs660240 | 1 | 109817838 | RA | HF | 8.53×10^-10^ |
| rs2069235 | 22 | 39747780 | RA | HF | 8.77×10^-10^ |
| rs17508548 | 6 | 29563067 | RA | HF | 1.03×10^-9^ |
| rs17208209 | 6 | 32195376 | RA | HF | 1.26×10^-9^ |
| rs2004640 | 7 | 128578301 | RA | HF | 1.26×10^-9^ |
| rs62407980 | 6 | 33013458 | RA | HF | 1.27×10^-9^ |
| rs3869097 | 6 | 30984470 | RA | HF | 1.39×10^-9^ |
| rs2561477 | 5 | 102608924 | RA | HF | 1.49×10^-9^ |
| rs35267732 | 6 | 31326779 | RA | HF | 1.57×10^-9^ |
| rs9368744 | 6 | 32798299 | RA | HF | 1.63×10^-9^ |
| rs1858037 | 2 | 65598300 | RA | HF | 1.67×10^-9^ |
| rs4746140 | 10 | 75417249 | RA | HF | 2.82×10^-9^ |
| rs17847933 | 6 | 33165780 | RA | HF | 2.9×10^-9^ |
| rs9295128 | 6 | 160751531 | RA | HF | 2.95×10^-9^ |
| rs10796038 | 10 | 6397964 | RA | HF | 5.71×10^-9^ |
| rs2531830 | 6 | 28380832 | RA | HF | 6.16×10^-9^ |
| rs2485363 | 6 | 159506121 | RA | HF | 6.34×10^-9^ |
| rs3094666 | 6 | 31104100 | RA | HF | 7.09×10^-9^ |
| rs28986321 | 6 | 32170980 | RA | HF | 8.63×10^-9^ |
| rs28411352 | 1 | 38278579 | RA | HF | 8.71×10^-9^ |
| rs56111157 | 6 | 31571894 | RA | HF | 9.32×10^-9^ |
| rs17617337 | 10 | 121426884 | RA | HF | 9.9×10^-9^ |
| rs592390 | 18 | 12822314 | RA | HF | 1.06×10^-8^ |
| rs3909112 | 6 | 31017256 | RA | HF | 1.12×10^-8^ |
| rs60733400 | 1 | 2516781 | RA | HF | 1.33×10^-8^ |
| rs9366829 | 6 | 33773199 | RA | HF | 1.39×10^-8^ |
| rs733590 | 6 | 36645203 | RA | HF | 1.53×10^-8^ |
| rs2301888 | 1 | 17672730 | RA | HF | 1.55×10^-8^ |
| rs42036 | 7 | 92241451 | RA | HF | 1.59×10^-8^ |
| rs8133843 | 21 | 36738242 | RA | HF | 1.68×10^-8^ |
| rs11758136 | 6 | 29764647 | RA | HF | 1.79×10^-8^ |
| rs2139492 | 16 | 86021627 | RA | HF | 2.69×10^-8^ |
| rs2105325 | 1 | 173349725 | RA | HF | 2.86×10^-8^ |
| rs600038 | 9 | 136151806 | RA | HF | 3.39×10^-8^ |
| rs73194058 | 21 | 34764288 | RA | HF | 4.52×10^-8^ |
| rs10985070 | 9 | 123636121 | RA | HF | 4.76×10^-8^ |
| rs2071538 | 6 | 32818678 | RA | HF | 4.81×10^-8^ |
| rs9267951 | 6 | 32212655 | RA | CAD | 0 |
| rs2281276 | 6 | 32340106 | RA | CAD | 0 |
| rs9268560 | 6 | 32389512 | RA | CAD | 0 |
| rs9275289 | 6 | 32663208 | RA | CAD | 0 |
| rs9271325 | 6 | 32582513 | RA | CAD | 7.02×10^-258^ |
| rs34422230 | 6 | 32776995 | RA | CAD | 1.75×10^-234^ |
| rs2856437 | 6 | 32157364 | RA | CAD | 1.1×10^-223^ |
| rs1794269 | 6 | 32673894 | RA | CAD | 3.54×10^-223^ |
| rs35502919 | 6 | 31604355 | RA | CAD | 1.24×10^-185^ |
| rs2073045 | 6 | 32339548 | RA | CAD | 5.78×10^-185^ |
| rs17421624 | 6 | 32066177 | RA | CAD | 3.52×10^-183^ |
| rs9272254 | 6 | 32603098 | RA | CAD | 3.59×10^-166^ |
| rs3132949 | 6 | 32195935 | RA | CAD | 5.06×10^-166^ |
| rs57912571 | 6 | 32750921 | RA | CAD | 7.6×10^-150^ |
| rs2476601 | 1 | 114377568 | RA | CAD | 4.35×10^-149^ |
| rs34492353 | 6 | 31520658 | RA | CAD | 7.06×10^-121^ |
| rs7767732 | 6 | 32853277 | RA | CAD | 5.72×10^-118^ |
| rs3128947 | 6 | 32965062 | RA | CAD | 5.32×10^-115^ |
| rs440841 | 6 | 33019643 | RA | CAD | 1.97×10^-107^ |
| rs2891168 | 9 | 22098619 | RA | CAD | 2.32×10^-101^ |
| rs35590025 | 6 | 30992111 | RA | CAD | 2.47×10^-101^ |
| rs3873444 | 6 | 32682724 | RA | CAD | 1.26×10^-100^ |
| rs2856822 | 6 | 33047432 | RA | CAD | 3×10^-96^ |
| rs7775228 | 6 | 32658079 | RA | CAD | 2.18×10^-95^ |
| rs3819716 | 6 | 32804284 | RA | CAD | 6.61×10^-94^ |
| rs3763355 | 6 | 32786882 | RA | CAD | 1.45×10^-93^ |
| rs9469427 | 6 | 33429275 | RA | CAD | 2.98×10^-92^ |
| rs1475865 | 6 | 31657413 | RA | CAD | 1.54×10^-87^ |
| rs62407970 | 6 | 32936994 | RA | CAD | 4.42×10^-86^ |
| rs2844509 | 6 | 31510924 | RA | CAD | 1.58×10^-81^ |
| rs41270488 | 6 | 32407906 | RA | CAD | 5.79×10^-81^ |
| rs2515919 | 6 | 31564167 | RA | CAD | 1.22×10^-74^ |
| rs56125791 | 6 | 32174743 | RA | CAD | 1.93×10^-74^ |
| rs2523454 | 6 | 31367865 | RA | CAD | 2.1×10^-74^ |
| rs9276831 | 6 | 32832033 | RA | CAD | 2.24×10^-71^ |
| rs11757367 | 6 | 32962882 | RA | CAD | 3.65×10^-64^ |
| rs1265093 | 6 | 31107187 | RA | CAD | 3.38×10^-62^ |
| rs2844503 | 6 | 31442731 | RA | CAD | 6.63×10^-61^ |
| rs9357094 | 6 | 30167476 | RA | CAD | 1.72×10^-58^ |
| rs41267649 | 6 | 33384473 | RA | CAD | 2.41×10^-58^ |
| rs9276757 | 6 | 32772856 | RA | CAD | 5.07×10^-55^ |
| rs28732209 | 6 | 32389543 | RA | CAD | 6.87×10^-54^ |
| rs3128948 | 6 | 32965085 | RA | CAD | 5.41×10^-53^ |
| rs2524084 | 6 | 31241639 | RA | CAD | 7.52×10^-52^ |
| rs11756897 | 6 | 32899139 | RA | CAD | 7.81×10^-52^ |
| rs9469120 | 6 | 32433057 | RA | CAD | 7.85×10^-52^ |
| rs8192585 | 6 | 32188823 | RA | CAD | 9.78×10^-52^ |
| rs9275601 | 6 | 32682664 | RA | CAD | 2.38×10^-51^ |
| rs35855550 | 6 | 32418722 | RA | CAD | 1.02×10^-49^ |
| rs9268909 | 6 | 32432340 | RA | CAD | 3.7×10^-49^ |
| rs10455872 | 6 | 161010118 | RA | CAD | 8.74×10^-48^ |
| rs35795092 | 6 | 32151420 | RA | CAD | 1×10^-46^ |
| rs6927872 | 6 | 31585624 | RA | CAD | 1.27×10^-44^ |
| rs552412 | 6 | 31777077 | RA | CAD | 1.09×10^-43^ |
| rs9391736 | 6 | 32196656 | RA | CAD | 1.18×10^-43^ |
| rs9275726 | 6 | 32688539 | RA | CAD | 1.19×10^-43^ |
| rs2844698 | 6 | 30931245 | RA | CAD | 3.87×10^-43^ |
| rs396090 | 6 | 32977535 | RA | CAD | 8.54×10^-43^ |
| rs206776 | 6 | 32953711 | RA | CAD | 1.01×10^-41^ |
| rs9266658 | 6 | 31347644 | RA | CAD | 3.03×10^-41^ |
| rs4713669 | 6 | 33697552 | RA | CAD | 4.18×10^-41^ |
| rs1318631 | 6 | 29635508 | RA | CAD | 4.96×10^-39^ |
| rs41267086 | 6 | 31847636 | RA | CAD | 5.58×10^-39^ |
| rs28622052 | 6 | 32857086 | RA | CAD | 3.58×10^-38^ |
| rs3130435 | 6 | 31219805 | RA | CAD | 1.48×10^-36^ |
| rs151719 | 6 | 32903900 | RA | CAD | 2.71×10^-36^ |
| rs187764 | 6 | 32902114 | RA | CAD | 2.79×10^-35^ |
| rs6922431 | 6 | 31465030 | RA | CAD | 3.59×10^-35^ |
| rs9349379 | 6 | 12903957 | RA | CAD | 1.06×10^-34^ |
| rs204892 | 6 | 32097891 | RA | CAD | 5.45×10^-34^ |
| rs4434456 | 6 | 33101064 | RA | CAD | 5.45×10^-33^ |
| rs17213554 | 6 | 32759694 | RA | CAD | 6.04×10^-33^ |
| rs3130048 | 6 | 31613739 | RA | CAD | 6.77×10^-33^ |
| rs154973 | 6 | 32900642 | RA | CAD | 8.27×10^-33^ |
| rs2894232 | 6 | 32011644 | RA | CAD | 3.23×10^-32^ |
| rs9405048 | 6 | 30670292 | RA | CAD | 5.05×10^-32^ |
| rs211453 | 6 | 33330131 | RA | CAD | 6.37×10^-32^ |
| rs3798220 | 6 | 160961137 | RA | CAD | 1.4×10^-31^ |
| rs72685677 | 1 | 113840826 | RA | CAD | 1.76×10^-31^ |
| rs3097683 | 6 | 32965382 | RA | CAD | 2.09×10^-31^ |
| rs28744296 | 6 | 33440416 | RA | CAD | 2.2×10^-31^ |
| rs4713470 | 6 | 31472821 | RA | CAD | 6.13×10^-31^ |
| rs6457718 | 6 | 33103827 | RA | CAD | 6.84×10^-31^ |
| rs3130183 | 6 | 33023996 | RA | CAD | 1.09×10^-30^ |
| rs12200756 | 6 | 33024258 | RA | CAD | 2.81×10^-30^ |
| rs9262602 | 6 | 31020271 | RA | CAD | 3.15×10^-30^ |
| rs9277648 | 6 | 33083816 | RA | CAD | 4.97×10^-30^ |
| rs9266635 | 6 | 31346979 | RA | CAD | 5.67×10^-30^ |
| rs7763502 | 6 | 30717637 | RA | CAD | 6.12×10^-30^ |
| rs4711222 | 6 | 30533275 | RA | CAD | 1.44×10^-29^ |
| rs12663370 | 6 | 33607373 | RA | CAD | 6.45×10^-29^ |
| rs3095150 | 6 | 30932532 | RA | CAD | 1.61×10^-28^ |
| rs11102712 | 1 | 114537037 | RA | CAD | 2.73×10^-28^ |
| rs12526104 | 6 | 32208882 | RA | CAD | 3.64×10^-28^ |
| rs2244839 | 6 | 31438368 | RA | CAD | 4.31×10^-28^ |
| rs12192933 | 6 | 32969390 | RA | CAD | 7.52×10^-28^ |
| rs3093998 | 6 | 31485174 | RA | CAD | 6.83×10^-27^ |
| rs7528419 | 1 | 109817192 | RA | CAD | 1.02×10^-26^ |
| rs2530696 | 6 | 30976496 | RA | CAD | 1.76×10^-26^ |
| rs2517514 | 6 | 31029401 | RA | CAD | 7.4×10^-26^ |
| rs2107202 | 6 | 30105743 | RA | CAD | 9.25×10^-26^ |
| rs73402255 | 6 | 31397805 | RA | CAD | 2.05×10^-25^ |
| rs9468976 | 6 | 31320473 | RA | CAD | 1.98×10^-24^ |
| rs7754218 | 6 | 33583446 | RA | CAD | 3.14×10^-24^ |
| rs9262661 | 6 | 31034592 | RA | CAD | 7.98×10^-24^ |
| rs41308311 | 6 | 31598675 | RA | CAD | 2.24×10^-23^ |
| rs3892710 | 6 | 32682862 | RA | CAD | 5.28×10^-23^ |
| rs28451064 | 21 | 35593827 | RA | CAD | 6.76×10^-23^ |
| rs3749946 | 6 | 31448862 | RA | CAD | 1.13×10^-22^ |
| rs35787014 | 6 | 29536498 | RA | CAD | 3.26×10^-22^ |
| rs3129987 | 6 | 30766204 | RA | CAD | 3.36×10^-22^ |
| rs2239800 | 6 | 32713267 | RA | CAD | 1.1×10^-21^ |
| rs7731626 | 5 | 55444683 | RA | CAD | 1.24×10^-21^ |
| rs4360170 | 6 | 31430359 | RA | CAD | 1.58×10^-21^ |
| rs6511720 | 19 | 11202306 | RA | CAD | 2.08×10^-21^ |
| rs3135022 | 6 | 33045966 | RA | CAD | 3.73×10^-21^ |
| rs35520989 | 6 | 31308887 | RA | CAD | 5.66×10^-21^ |
| rs6911704 | 6 | 31276245 | RA | CAD | 6.11×10^-21^ |
| rs4318925 | 6 | 32761506 | RA | CAD | 1.44×10^-20^ |
| rs13201129 | 6 | 30601067 | RA | CAD | 4.03×10^-20^ |
| rs13194698 | 6 | 31914673 | RA | CAD | 1.07×10^-19^ |
| rs17179851 | 6 | 29924440 | RA | CAD | 1.37×10^-19^ |
| rs17207986 | 6 | 32079567 | RA | CAD | 2.1×10^-19^ |
| rs12211782 | 6 | 30984878 | RA | CAD | 4.68×10^-19^ |
| rs11755596 | 6 | 32691500 | RA | CAD | 4.71×10^-19^ |
| rs7412 | 19 | 45412079 | RA | CAD | 5.63×10^-19^ |
| rs6922309 | 6 | 31321238 | RA | CAD | 6.03×10^-19^ |
| rs3087243 | 2 | 204738919 | RA | CAD | 9.85×10^-19^ |
| rs17264332 | 6 | 138005515 | RA | CAD | 1.9×10^-18^ |
| rs72934765 | 2 | 203758551 | RA | CAD | 2.11×10^-18^ |
| rs41316606 | 6 | 28862516 | RA | CAD | 2.3×10^-18^ |
| rs9261819 | 6 | 30378989 | RA | CAD | 2.35×10^-18^ |
| rs10774625 | 12 | 111910219 | RA | CAD | 2.97×10^-18^ |
| rs35085781 | 6 | 33015421 | RA | CAD | 3.84×10^-18^ |
| rs58721818 | 6 | 138243739 | RA | CAD | 6.73×10^-18^ |
| rs1217195 | 1 | 114178764 | RA | CAD | 8.07×10^-18^ |
| rs12527959 | 6 | 29537426 | RA | CAD | 1.01×10^-17^ |
| rs11751928 | 6 | 28335378 | RA | CAD | 1.57×10^-17^ |
| rs7170107 | 15 | 70010647 | RA | CAD | 7.59×10^-17^ |
| rs7164479 | 15 | 79123054 | RA | CAD | 9.17×10^-17^ |
| rs35404844 | 6 | 33269881 | RA | CAD | 9.53×10^-17^ |
| rs34087996 | 6 | 31329062 | RA | CAD | 1.46×10^-16^ |
| rs9262562 | 6 | 31008949 | RA | CAD | 1.47×10^-16^ |
| rs34046593 | 4 | 26111593 | RA | CAD | 2.49×10^-16^ |
| rs73400019 | 6 | 33034166 | RA | CAD | 6.05×10^-16^ |
| rs4713424 | 6 | 31002742 | RA | CAD | 8.6×10^-16^ |
| rs2246942 | 10 | 91004886 | RA | CAD | 8.87×10^-16^ |
| rs34536443 | 19 | 10463118 | RA | CAD | 1.27×10^-15^ |
| rs210194 | 6 | 33482146 | RA | CAD | 1.69×10^-15^ |
| rs773588 | 1 | 113844824 | RA | CAD | 2.25×10^-15^ |
| rs4947324 | 6 | 31528130 | RA | CAD | 3.86×10^-15^ |
| rs10456419 | 6 | 33214857 | RA | CAD | 4.99×10^-15^ |
| rs17404424 | 6 | 29275298 | RA | CAD | 5.1×10^-15^ |
| rs9295939 | 6 | 30953968 | RA | CAD | 5.59×10^-15^ |
| rs9277009 | 6 | 32992036 | RA | CAD | 8.72×10^-15^ |
| rs10790268 | 11 | 118729391 | RA | CAD | 9.1×10^-15^ |
| rs35329444 | 6 | 33588985 | RA | CAD | 9.8×10^-15^ |
| rs6905389 | 6 | 30319930 | RA | CAD | 9.81×10^-15^ |
| rs1571878 | 6 | 167540842 | RA | CAD | 1.35×10^-14^ |
| rs7761859 | 6 | 33804137 | RA | CAD | 1.88×10^-14^ |
| rs6933672 | 6 | 28941688 | RA | CAD | 4.82×10^-14^ |
| rs17200698 | 6 | 31483700 | RA | CAD | 6.19×10^-14^ |
| rs9457761 | 6 | 160292603 | RA | CAD | 6.53×10^-14^ |
| rs4711363 | 6 | 33852948 | RA | CAD | 6.66×10^-14^ |
| rs1250229 | 2 | 216304384 | RA | CAD | 1.14×10^-13^ |
| rs9457995 | 6 | 161102643 | RA | CAD | 1.14×10^-13^ |
| rs4239702 | 20 | 44749251 | RA | CAD | 1.14×10^-13^ |
| rs34695944 | 2 | 61124850 | RA | CAD | 1.23×10^-13^ |
| rs56131196 | 19 | 45422846 | RA | CAD | 1.57×10^-13^ |
| rs17696736 | 12 | 112486818 | RA | CAD | 1.89×10^-13^ |
| rs67180937 | 1 | 222823743 | RA | CAD | 2.15×10^-13^ |
| rs55811970 | 1 | 114355237 | RA | CAD | 2.72×10^-13^ |
| rs34018127 | 6 | 30122978 | RA | CAD | 3.01×10^-13^ |
| rs72743461 | 15 | 67441750 | RA | CAD | 3.21×10^-13^ |
| rs9261408 | 6 | 30074749 | RA | CAD | 3.29×10^-13^ |
| rs12202017 | 6 | 134173151 | RA | CAD | 4.82×10^-13^ |
| rs35187679 | 15 | 69982695 | RA | CAD | 5.98×10^-13^ |
| rs73432769 | 6 | 30716170 | RA | CAD | 6.32×10^-13^ |
| rs13196770 | 6 | 32965363 | RA | CAD | 7.02×10^-13^ |
| rs17207895 | 6 | 32020512 | RA | CAD | 7.22×10^-13^ |
| rs7177201 | 15 | 79065380 | RA | CAD | 8.06×10^-13^ |
| rs2107595 | 7 | 19049388 | RA | CAD | 8.82×10^-13^ |
| rs707937 | 6 | 31731014 | RA | CAD | 1.03×10^-12^ |
| rs11575848 | 6 | 31686943 | RA | CAD | 1.28×10^-12^ |
| rs2071382 | 15 | 91428197 | RA | CAD | 1.85×10^-12^ |
| rs3135460 | 6 | 32996773 | RA | CAD | 1.95×10^-12^ |
| rs13191067 | 6 | 32925407 | RA | CAD | 2.02×10^-12^ |
| rs17184883 | 6 | 29720175 | RA | CAD | 2.14×10^-12^ |
| rs11574914 | 9 | 34710338 | RA | CAD | 2.28×10^-12^ |
| rs6905073 | 6 | 161067892 | RA | CAD | 2.72×10^-12^ |
| rs7568458 | 2 | 85788175 | RA | CAD | 2.77×10^-12^ |
| rs17114046 | 1 | 56966350 | RA | CAD | 3.21×10^-12^ |
| rs2954029 | 8 | 126490972 | RA | CAD | 3.6×10^-12^ |
| rs71508903 | 10 | 63779871 | RA | CAD | 3.64×10^-12^ |
| rs3918226 | 7 | 150690176 | RA | CAD | 4.17×10^-12^ |
| rs72881217 | 6 | 33119305 | RA | CAD | 5.11×10^-12^ |
| rs13426947 | 2 | 191933254 | RA | CAD | 6.5×10^-12^ |
| rs8032939 | 15 | 38834033 | RA | CAD | 6.51×10^-12^ |
| rs3131059 | 6 | 30763004 | RA | CAD | 6.82×10^-12^ |
| rs1565660 | 10 | 44538672 | RA | CAD | 7.79×10^-12^ |
| rs11968393 | 6 | 32779073 | RA | CAD | 8.36×10^-12^ |
| rs9653442 | 2 | 100825367 | RA | CAD | 9.46×10^-12^ |
| rs61819426 | 1 | 114137902 | RA | CAD | 9.7×10^-12^ |
| rs11556924 | 7 | 129663496 | RA | CAD | 9.84×10^-12^ |
| rs773566 | 1 | 113821584 | RA | CAD | 1.29×10^-11^ |
| rs56297443 | 6 | 32832037 | RA | CAD | 2.18×10^-11^ |
| rs507666 | 9 | 136149399 | RA | CAD | 2.74×10^-11^ |
| rs9260918 | 6 | 29948751 | RA | CAD | 3.38×10^-11^ |
| rs1887318 | 10 | 30321598 | RA | CAD | 3.7×10^-11^ |
| rs2535240 | 6 | 29643877 | RA | CAD | 4.55×10^-11^ |
| rs34381587 | 7 | 128666825 | RA | CAD | 4.8×10^-11^ |
| rs7746977 | 6 | 33842481 | RA | CAD | 6.05×10^-11^ |
| rs1039765 | 2 | 65614362 | RA | CAD | 6.43×10^-11^ |
| rs1746050 | 10 | 44777188 | RA | CAD | 6.61×10^-11^ |
| rs41266809 | 6 | 26205209 | RA | CAD | 6.64×10^-11^ |
| rs706778 | 10 | 6098949 | RA | CAD | 7.38×10^-11^ |
| rs4546922 | 1 | 114066063 | RA | CAD | 1.07×10^-10^ |
| rs360157 | 11 | 9754221 | RA | CAD | 1.23×10^-10^ |
| rs58667488 | 6 | 32431785 | RA | CAD | 1.24×10^-10^ |
| rs9603608 | 13 | 40318819 | RA | CAD | 1.28×10^-10^ |
| rs17213728 | 6 | 32781954 | RA | CAD | 1.39×10^-10^ |
| rs11085727 | 19 | 10466123 | RA | CAD | 1.74×10^-10^ |
| rs452850 | 6 | 29799481 | RA | CAD | 1.87×10^-10^ |
| rs12663679 | 6 | 28990049 | RA | CAD | 1.89×10^-10^ |
| rs2681472 | 12 | 90008959 | RA | CAD | 1.97×10^-10^ |
| rs707922 | 6 | 31625507 | RA | CAD | 2.26×10^-10^ |
| rs56144236 | 6 | 32720219 | RA | CAD | 2.36×10^-10^ |
| rs1980421 | 2 | 204610004 | RA | CAD | 3.05×10^-10^ |
| rs185244 | 3 | 138092889 | RA | CAD | 4.32×10^-10^ |
| rs180803 | 22 | 24658858 | RA | CAD | 4.37×10^-10^ |
| rs11838776 | 13 | 111040681 | RA | CAD | 4.5×10^-10^ |
| rs2839812 | 11 | 103673294 | RA | CAD | 4.68×10^-10^ |
| rs13288666 | 9 | 21973857 | RA | CAD | 4.77×10^-10^ |
| rs7753474 | 6 | 29341308 | RA | CAD | 5.09×10^-10^ |
| rs7897792 | 10 | 8108290 | RA | CAD | 5.09×10^-10^ |
| rs2421206 | 19 | 11262477 | RA | CAD | 5.18×10^-10^ |
| rs28359997 | 6 | 29717911 | RA | CAD | 6.79×10^-10^ |
| rs4452313 | 3 | 17047032 | RA | CAD | 7.3×10^-10^ |
| rs11591147 | 1 | 55505647 | RA | CAD | 7.31×10^-10^ |
| rs4576655 | 1 | 154418749 | RA | CAD | 8.85×10^-10^ |
| rs130068 | 6 | 31116246 | RA | CAD | 9.87×10^-10^ |
| rs2069235 | 22 | 39747780 | RA | CAD | 1.06×10^-9^ |
| rs2004640 | 7 | 128578301 | RA | CAD | 1.17×10^-9^ |
| rs6841581 | 4 | 148401190 | RA | CAD | 1.19×10^-9^ |
| rs2561477 | 5 | 102608924 | RA | CAD | 1.39×10^-9^ |
| rs1264704 | 6 | 30065319 | RA | CAD | 1.54×10^-9^ |
| rs624249 | 6 | 160679400 | RA | CAD | 1.58×10^-9^ |
| rs2451258 | 6 | 159506600 | RA | CAD | 1.69×10^-9^ |
| rs2308622 | 6 | 31238029 | RA | CAD | 2.04×10^-9^ |
| rs4773141 | 13 | 110954353 | RA | CAD | 2.4×10^-9^ |
| rs11617955 | 13 | 110818102 | RA | CAD | 2.88×10^-9^ |
| rs73399211 | 6 | 32432464 | RA | CAD | 2.95×10^-9^ |
| rs8068952 | 17 | 59286644 | RA | CAD | 2.96×10^-9^ |
| rs7098414 | 10 | 82214586 | RA | CAD | 3.08×10^-9^ |
| rs12693989 | 2 | 204271511 | RA | CAD | 3.14×10^-9^ |
| rs2306556 | 4 | 156638573 | RA | CAD | 3.16×10^-9^ |
| rs60733400 | 1 | 2516781 | RA | CAD | 3.3×10^-9^ |
| rs9366829 | 6 | 33773199 | RA | CAD | 3.33×10^-9^ |
| rs12493885 | 3 | 153839866 | RA | CAD | 3.73×10^-9^ |
| rs10796038 | 10 | 6397964 | RA | CAD | 5.29×10^-9^ |
| rs9368744 | 6 | 32798299 | RA | CAD | 5.65×10^-9^ |
| rs362518 | 6 | 29567638 | RA | CAD | 7.05×10^-9^ |
| rs16986953 | 2 | 19942473 | RA | CAD | 7.1×10^-9^ |
| rs4299376 | 2 | 44072576 | RA | CAD | 7.12×10^-9^ |
| rs56111157 | 6 | 31571894 | RA | CAD | 8.64×10^-9^ |
| rs1002985 | 6 | 32852448 | RA | CAD | 9.03×10^-9^ |
| rs2244608 | 12 | 121416988 | RA | CAD | 9.09×10^-9^ |
| rs2531830 | 6 | 28380832 | RA | CAD | 9.53×10^-9^ |
| rs7623687 | 3 | 49448566 | RA | CAD | 9.56×10^-9^ |
| rs592390 | 18 | 12822314 | RA | CAD | 9.81×10^-9^ |
| rs7500448 | 16 | 83045790 | RA | CAD | 9.98×10^-9^ |
| rs34073687 | 17 | 38007323 | RA | CAD | 1.15×10^-8^ |
| rs28411352 | 1 | 38278579 | RA | CAD | 1.21×10^-8^ |
| rs11191416 | 10 | 104604916 | RA | CAD | 1.41×10^-8^ |
| rs2301888 | 1 | 17672730 | RA | CAD | 1.44×10^-8^ |
| rs13213216 | 6 | 31327738 | RA | CAD | 1.54×10^-8^ |
| rs8133843 | 21 | 36738242 | RA | CAD | 1.56×10^-8^ |
| rs28986321 | 6 | 32170980 | RA | CAD | 1.56×10^-8^ |
| rs36228834 | 9 | 21975319 | RA | CAD | 1.71×10^-8^ |
| rs12928974 | 16 | 75304957 | RA | CAD | 1.94×10^-8^ |
| rs1633360 | 12 | 58108052 | RA | CAD | 1.97×10^-8^ |
| rs62407980 | 6 | 33013458 | RA | CAD | 2.14×10^-8^ |
| rs61828284 | 1 | 173299743 | RA | CAD | 2.26×10^-8^ |
| rs62097857 | 18 | 12857758 | RA | CAD | 2.32×10^-8^ |
| rs395671 | 6 | 33553677 | RA | CAD | 2.33×10^-8^ |
| rs12663194 | 6 | 33811207 | RA | CAD | 2.39×10^-8^ |
| rs62265630 | 3 | 124475201 | RA | CAD | 2.51×10^-8^ |
| rs617328 | 6 | 138000398 | RA | CAD | 2.57×10^-8^ |
| rs531819 | 2 | 21263639 | RA | CAD | 2.77×10^-8^ |
| rs28780086 | 6 | 30105154 | RA | CAD | 2.97×10^-8^ |
| rs10139550 | 14 | 100145710 | RA | CAD | 2.98×10^-8^ |
| rs9276964 | 6 | 32964967 | RA | CAD | 3.16×10^-8^ |
| rs1855185 | 9 | 22073996 | RA | CAD | 3.61×10^-8^ |
| rs11057830 | 12 | 125307053 | RA | CAD | 3.85×10^-8^ |
| rs56132765 | 2 | 44078853 | RA | CAD | 3.93×10^-8^ |
| rs2760740 | 17 | 2020989 | RA | CAD | 4.56×10^-8^ |
| rs17087335 | 4 | 57838583 | RA | CAD | 4.63×10^-8^ |
| rs13200993 | 6 | 22612912 | RA | CAD | 4.80×10^-8^ |
| rs2139493 | 16 | 86021624 | RA | CAD | 4.81×10^-8^ |
| rs4593108 | 4 | 148281001 | RA | CAD | 4.84×10^-8^ |

Abbreviations: AF, atrial fibrillation; CAD, coronary artery disease; HF, heart failure; RA, rheumatoid arthritis.

**Table S11.** The results of histone modification and enhancer enrichment for lead SNPs.

| SNP | symbol | Source | EnhID |
| --- | --- | --- | --- |
| rs42034 | Ctcf | GM12878 | NA |
| rs4750517 | Ctcf | GM12878 | NA |
| rs7098414 | Ctcf | GM12878 | NA |
| rs12022363 | Ezh239875 | GM12878 | NA |
| rs11552449 | Ezh239875 | GM12878 | NA |
| rs12126142 | Ezh239875 | GM12878 | NA |
| rs62149420 | Ezh239875 | GM12878 | NA |
| rs700677 | Ezh239875 | GM12878 | NA |
| rs42034 | Ezh239875 | GM12878 | NA |
| rs42039 | Ezh239875 | GM12878 | NA |
| rs4750517 | Ezh239875 | GM12878 | NA |
| rs10840298 | Ezh239875 | GM12878 | NA |
| rs9747973 | Ezh239875 | GM12878 | NA |
| rs11658278 | Ezh239875 | GM12878 | NA |
| rs2297199 | Ezh239875 | GM12878 | NA |
| rs6074012 | Ezh239875 | GM12878 | NA |
| rs11552449 | H2az | GM12878 | NA |
| rs11552449 | H2az | GM12878 | NA |
| rs62149420 | H2az | GM12878 | NA |
| rs700677 | H2az | GM12878 | NA |
| rs409558 | H2az | GM12878 | NA |
| rs7098414 | H2az | GM12878 | NA |
| rs11552449 | H3k4me1 | GM12878 | NA |
| rs62149420 | H3k4me1 | GM12878 | NA |
| rs62149420 | H3k4me1 | GM12878 | NA |
| rs409558 | H3k4me1 | GM12878 | NA |
| rs42034 | H3k4me1 | GM12878 | NA |
| rs42034 | H3k4me1 | GM12878 | NA |
| rs42039 | H3k4me1 | GM12878 | NA |
| rs42039 | H3k4me1 | GM12878 | NA |
| rs10840298 | H3k4me1 | GM12878 | NA |
| rs10774624 | H3k4me1 | GM12878 | NA |
| rs12919951 | H3k4me1 | GM12878 | NA |
| rs12919951 | H3k4me1 | GM12878 | NA |
| rs9747973 | H3k4me1 | GM12878 | NA |
| rs7248558 | H3k4me1 | GM12878 | NA |
| rs7248558 | H3k4me1 | GM12878 | NA |
| rs2843151 | H3k4me2 | GM12878 | NA |
| rs11552449 | H3k4me2 | GM12878 | NA |
| rs409558 | H3k4me2 | GM12878 | NA |
| rs7098414 | H3k4me2 | GM12878 | NA |
| rs9747973 | H3k4me2 | GM12878 | NA |
| rs11658278 | H3k4me2 | GM12878 | NA |
| rs7248558 | H3k4me2 | GM12878 | NA |
| rs2843151 | H3k4me3 | GM12878 | NA |
| rs11552449 | H3k4me3 | GM12878 | NA |
| rs11552449 | H3k4me3 | GM12878 | NA |
| rs62149420 | H3k4me3 | GM12878 | NA |
| rs700677 | H3k4me3 | GM12878 | NA |
| rs409558 | H3k4me3 | GM12878 | NA |
| rs42039 | H3k4me3 | GM12878 | NA |
| rs7098414 | H3k4me3 | GM12878 | NA |
| rs7098414 | H3k4me3 | GM12878 | NA |
| rs9747973 | H3k4me3 | GM12878 | NA |
| rs11658278 | H3k4me3 | GM12878 | NA |
| rs7248558 | H3k4me3 | GM12878 | NA |
| rs62149420 | H3k9me3 | GM12878 | NA |
| rs62149420 | H3k9me3 | GM12878 | NA |
| rs42039 | H3k9me3 | GM12878 | NA |
| rs4750517 | H3k9me3 | GM12878 | NA |
| rs7098414 | H3k9me3 | GM12878 | NA |
| rs10840298 | H3k9me3 | GM12878 | NA |
| rs8075737 | H3k9me3 | GM12878 | NA |
| rs11658278 | H3k9me3 | GM12878 | NA |
| rs11552449 | H3k27ac | GM12878 | NA |
| rs409558 | H3k27ac | GM12878 | NA |
| rs42034 | H3k27ac | GM12878 | NA |
| rs42039 | H3k27ac | GM12878 | NA |
| rs7098414 | H3k27ac | GM12878 | NA |
| rs12919951 | H3k27ac | GM12878 | NA |
| rs11658278 | H3k27ac | GM12878 | NA |
| rs700677 | H3k27me3 | GM12878 | NA |
| rs4750517 | H3k27me3 | GM12878 | NA |
| rs4749532 | H3k27me3 | GM12878 | NA |
| rs2297199 | H3k27me3 | GM12878 | NA |
| rs11552449 | H3k36me3 | GM12878 | NA |
| rs12126142 | H3k36me3 | GM12878 | NA |
| rs62149420 | H3k36me3 | GM12878 | NA |
| rs4563251 | H3k36me3 | GM12878 | NA |
| rs2454429 | H3k36me3 | GM12878 | NA |
| rs409558 | H3k36me3 | GM12878 | NA |
| rs42034 | H3k36me3 | GM12878 | NA |
| rs42039 | H3k36me3 | GM12878 | NA |
| rs7098414 | H3k36me3 | GM12878 | NA |
| rs10840298 | H3k36me3 | GM12878 | NA |
| rs8075737 | H3k36me3 | GM12878 | NA |
| rs7248558 | H3k36me3 | GM12878 | NA |
| rs11552449 | H3k79me2 | GM12878 | NA |
| rs2454429 | H3k79me2 | GM12878 | NA |
| rs409558 | H3k79me2 | GM12878 | NA |
| rs42034 | H3k79me2 | GM12878 | NA |
| rs42039 | H3k79me2 | GM12878 | NA |
| rs7098414 | H3k79me2 | GM12878 | NA |
| rs10840298 | H3k79me2 | GM12878 | NA |
| rs10774624 | H3k79me2 | GM12878 | NA |
| rs12919951 | H3k79me2 | GM12878 | NA |
| rs9747973 | H3k79me2 | GM12878 | NA |
| rs11658278 | H3k79me2 | GM12878 | NA |
| rs7248558 | H3k79me2 | GM12878 | NA |
| rs2421206 | H3k79me2 | GM12878 | NA |
| rs12126142 | H4k20me1 | GM12878 | NA |
| rs2454429 | H4k20me1 | GM12878 | NA |
| rs2905734 | H4k20me1 | GM12878 | NA |
| rs7098414 | H4k20me1 | GM12878 | NA |
| rs8075737 | H4k20me1 | GM12878 | NA |
| rs7248558 | H4k20me1 | GM12878 | NA |
| rs2297199 | H4k20me1 | GM12878 | NA |
| rs6074012 | H4k20me1 | GM12878 | NA |
| rs2843151 | Ctcf | Huvec | NA |
| rs2454429 | Ctcf | Huvec | NA |
| rs2905734 | Ctcf | Huvec | NA |
| rs2905734 | Ctcf | Huvec | NA |
| rs7098414 | Ctcf | Huvec | NA |
| rs2454429 | Ezh239875 | Huvec | NA |
| rs9747973 | Ezh239875 | Huvec | NA |
| rs2843151 | H3k4me1 | Huvec | NA |
| rs11552449 | H3k4me1 | Huvec | NA |
| rs11552449 | H3k4me1 | Huvec | NA |
| rs62149420 | H3k4me1 | Huvec | NA |
| rs409558 | H3k4me1 | Huvec | NA |
| rs7098414 | H3k4me1 | Huvec | NA |
| rs10774624 | H3k4me1 | Huvec | NA |
| rs12919951 | H3k4me1 | Huvec | NA |
| rs2843151 | H3k4me2 | Huvec | NA |
| rs11552449 | H3k4me2 | Huvec | NA |
| rs62149420 | H3k4me2 | Huvec | NA |
| rs409558 | H3k4me2 | Huvec | NA |
| rs7098414 | H3k4me2 | Huvec | NA |
| rs7098414 | H3k4me2 | Huvec | NA |
| rs2843151 | H3k4me3 | Huvec | NA |
| rs11552449 | H3k4me3 | Huvec | NA |
| rs409558 | H3k4me3 | Huvec | NA |
| rs7098414 | H3k4me3 | Huvec | NA |
| rs2843151 | H3k9ac | Huvec | NA |
| rs2843151 | H3k9ac | Huvec | NA |
| rs11552449 | H3k9ac | Huvec | NA |
| rs409558 | H3k9ac | Huvec | NA |
| rs7098414 | H3k9ac | Huvec | NA |
| rs7098414 | H3k9ac | Huvec | NA |
| rs12022363 | H3k9me1 | Huvec | NA |
| rs11552449 | H3k9me1 | Huvec | NA |
| rs62149420 | H3k9me1 | Huvec | NA |
| rs4563251 | H3k9me1 | Huvec | NA |
| rs700677 | H3k9me1 | Huvec | NA |
| rs2454429 | H3k9me1 | Huvec | NA |
| rs2905734 | H3k9me1 | Huvec | NA |
| rs409558 | H3k9me1 | Huvec | NA |
| rs42034 | H3k9me1 | Huvec | NA |
| rs42039 | H3k9me1 | Huvec | NA |
| rs4749532 | H3k9me1 | Huvec | NA |
| rs7098414 | H3k9me1 | Huvec | NA |
| rs10774624 | H3k9me1 | Huvec | NA |
| rs12919951 | H3k9me1 | Huvec | NA |
| rs8075737 | H3k9me1 | Huvec | NA |
| rs7248558 | H3k9me1 | Huvec | NA |
| rs6074012 | H3k9me1 | Huvec | NA |
| rs2843151 | H3k27ac | Huvec | NA |
| rs11552449 | H3k27ac | Huvec | NA |
| rs409558 | H3k27ac | Huvec | NA |
| rs409558 | H3k27ac | Huvec | NA |
| rs7098414 | H3k27ac | Huvec | NA |
| rs10840298 | H3k27ac | Huvec | NA |
| rs10774624 | H3k27ac | Huvec | NA |
| rs12022363 | H3k27me3 | Huvec | NA |
| rs2905734 | H3k27me3 | Huvec | NA |
| rs3130683 | H3k27me3 | Huvec | NA |
| rs13277738 | H3k27me3 | Huvec | NA |
| rs9747973 | H3k27me3 | Huvec | NA |
| rs11658278 | H3k27me3 | Huvec | NA |
| rs2843151 | H3k36me3 | Huvec | NA |
| rs2454429 | H3k36me3 | Huvec | NA |
| rs2454429 | H3k36me3 | Huvec | NA |
| rs42034 | H3k36me3 | Huvec | NA |
| rs42039 | H3k36me3 | Huvec | NA |
| rs7098414 | H3k36me3 | Huvec | NA |
| rs10840298 | H3k36me3 | Huvec | NA |
| rs7248558 | H3k36me3 | Huvec | NA |
| rs6074012 | H3k36me3 | Huvec | NA |
| rs2843151 | H3k79me2 | Huvec | NA |
| rs11552449 | H3k79me2 | Huvec | NA |
| rs2905734 | H3k79me2 | Huvec | NA |
| rs409558 | H3k79me2 | Huvec | NA |
| rs7098414 | H3k79me2 | Huvec | NA |
| rs10840298 | H3k79me2 | Huvec | NA |
| rs12919951 | H3k79me2 | Huvec | NA |
| rs7248558 | H3k79me2 | Huvec | NA |
| rs2421206 | H3k79me2 | Huvec | NA |
| rs6074012 | H3k79me2 | Huvec | NA |
| rs700677 | H4k20me1 | Huvec | NA |
| rs2454429 | H4k20me1 | Huvec | NA |
| rs42034 | H4k20me1 | Huvec | NA |
| rs42039 | H4k20me1 | Huvec | NA |
| rs7098414 | H4k20me1 | Huvec | NA |
| rs10840298 | H4k20me1 | Huvec | NA |
| rs7248558 | H4k20me1 | Huvec | NA |
| rs6074012 | H4k20me1 | Huvec | NA |
| rs2843151 | Pol2b | Huvec | NA |
| rs2454429 | Pol2b | Huvec | NA |
| rs409558 | Pol2b | Huvec | NA |
| rs409558 | Pol2b | Huvec | NA |
| rs42034 | Pol2b | Huvec | NA |
| rs7098414 | Pol2b | Huvec | NA |
| rs10840298 | Pol2b | Huvec | NA |
| rs10774624 | Pol2b | Huvec | NA |
| rs7248558 | Pol2b | Huvec | NA |
| rs7248558 | Pol2b | Huvec | NA |
| rs2421206 | Pol2b | Huvec | NA |
| rs2421206 | Pol2b | Huvec | NA |
| rs2843151 | Enhancer | ENCODE | 669 |
| rs2843151 | Enhancer | Roadmap | 466424 |
| rs4563251 | Enhancer | ENCODE | 49983 |
| rs42039 | Enhancer | Roadmap | 1231198 |
| rs13277738 | Enhancer | Roadmap | 1311411 |

**Table S12.** The results of summary-based Mendelian randomization analyses and colocalization results

| Candidate gene | beta_SMR | se_SMR | *P*_SMR | *P*_HEIDI | No. of SNPs for HEIDI test | Traits | Source(tissue) | PP.H0.abf | PP.H1.abf | PP.H2.abf | PP.H3.abf | PP.H4.abf |
| --- | --- | --- | --- | --- | --- | --- | --- | --- | --- | --- | --- | --- |
| MKRN2 | 0.07 | 0.02 | 9.43×10^-5^ | 0.177 | 20 | AF | eQTLGEN | 1.53×10^-319^ | 3.8×10^-302^ | 4.0×10^-18^ | 1.0 | 3.3×10^-17^ |
| MKRN2 | 0.05 | 0.02 | 1.62×10^-2^ | 0.149 | 20 | AF | GTEx  (artery aorta) | 1.7×10^-26^ | 2.9×10^-9^ | 5.9×10^-18^ | 1.0 | 3.1×10^-10^ |
| MKRN2 | 0.14 | 0.04 | 1.38×10^-3^ | 0.345 | 14 | AF | GTEx  (blood) | 2.3×10^-18^ | 4.0×10^-1^ | 3.4×10^-18^ | 0.58 | 0.028 |
| MKRN2 | 0.07 | 0.02 | 4.83×10^-3^ | 0.612 | 20 | AF | GTEx  (heart artial appendage) | 5.9×10^-22^ | 1.0×10^-4^ | 5.9×10^-18^ | 1.0 | 4.4×10^-4^ |
| MKRN2 | 0.06 | 0.02 | 4.97×10^-3^ | 0.414 | 20 | AF | GTEx  ( Heart left ventricle) | 2.9×10^-26^ | 5.0×10^-9^ | 5.9×10^-18^ | 1.0 | 5.7×10^-8^ |
| MKRN2 | -0.11 | 0.05 | 3.34×10^-2^ | 0.071 | 20 | RA | eQTLGEN | 4.2×10^-302^ | 8.1×10^-302^ | 0.33 | 0.63 | 0.043 |
| MKRN2 | -0.23 | 0.11 | 4.73×10^-2^ | 0.597 | 6 | RA | GTEx  (blood) | 1.9×10^-1^ | 2.4×10^-1^ | 0.24 | 0.3 | 0.041 |
| MKRN2 | 0.06 | 0.03 | 3.02×10^-2^ | 0.396 | 20 | Stroke | GTEx  (Heart left ventricle) | 4.6×10^-9^ | 6.3×10^-10^ | 0.88 | 0.12 | 4.4×10^-3^ |
| PHETA1 | -0.21 | 0.06 | 1.45×10^-3^ | 0.066 | 20 | CAD | eQTLGEN | 7.9×10^-60^ | 3.0×10^-53^ | 2.6×10^-7^ | 1.0 | 1.0×10^-6^ |
| PHETA1 | -0.23 | 0.06 | 3.93×10^-4^ | 0.254 | 20 | HF | eQTLGEN | 5.3×10^-55^ | 2.8×10^-53^ | 0.18 | 0.92 | 0.062 |
| PHETA1 | -0.35 | 0.13 | 6.29×10^-3^ | 0.132 | 20 | RA | eQTLGEN | 1.4×10^-54^ | 2.8×10^-53^ | 0.048 | 0.93 | 0.022 |
| PHETA1 | -0.35 | 0.08 | 3.83×10^-6^ | 0.42 | 20 | Stroke | eQTLGEN | 1.5×10^-58^ | 3.0×10^-53^ | 5.1×10^-6^ | 1.0 | 1.4×10^-3^ |
| PLCL1 | 0.06 | 0.01 | 5.76×10^-5^ | 0.063 | 20 | AF | eQTLGEN | 4.8×10^-307^ | 3.8×10^-305^ | 6.7×10^-3^ | 0.52 | 0.47 |
| PLCL1 | -0.15 | 0.04 | 1.77×10^-4^ | 0.107 | 20 | RA | eQTLGEN | 4.1×10^-306^ | 1.7×10^-305^ | 0.053 | 0.22 | 0.73 |
| SWAP70 | -0.16 | 0.03 | 1.97×10^-7^ | 0.055 | 20 | CAD | eQTLGEN | 4.8×10^-261^ | 2.2×10^-258^ | 2.9×10^-4^ | 0.13 | 0.87 |
| SWAP70 | -0.1 | 0.03 | 6.24×10^-4^ | 0.065 | 20 | HF | eQTLGEN | 6.9×10^-258^ | 1.2×10^-258^ | 0.41 | 0.068 | 0.52 |
| SWAP70 | -0.24 | 0.07 | 1.27×10^-3^ | 0.491 | 20 | RA | eQTLGEN | 6.0×10^-258^ | 1.4×10^-258^ | 0.36 | 0.083 | 0.56 |

Abbreviations: AF, atrial fibrillation; CAD, coronary artery disease; HF, heart failure; RA, rheumatoid arthritis.

**Table S13.** The enriched GO terms of the mapped genes.

| Term | ID | GO domains | Input number | Background number | *P* | FDR | Input |
| --- | --- | --- | --- | --- | --- | --- | --- |
| actin cytoskeleton | GO:0015629 | CC | 5 | 245 | 7.23×10^-7^ | 2.76E-4 | C2\|SWAP70\|BCAR1\|ILF3\|NCOA5 |
| protein homodimerization activity | GO:0042803 | MF | 6 | 660 | 5.08×10^-6^ | 0.001 | BAK1\|C2\|IL6R\|DCLRE1B\|IKZF3\|PHETA1 |
| protein binding | GO:0005515 | MF | 19 | 11779 | 1.81×10^-5^ | 0.002 | ILF3\|NCOA5\|CDK12\|DCLRE1B\|ZBTB9\|BAK1\|TSPAN14\|IL6R\|MKRN2\|SH2B3\|CDK6\|NAT2\|PRKCQ\|C2\|SWAP70\|SPC24\|BCAR1\|IKZF3\|PHETA1 |
| RNA polymerase II CTD heptapeptide repeat kinase activity | GO:0008353 | MF | 2 | 12 | 4.12×10^-5^ | 0.004 | CDK12\|CDK6 |
| regulation of cell cycle | GO:0051726 | BP | 3 | 121 | 8.50×10^-5^ | 0.006 | CDK12\|CDK6\|BAK1 |
| endocrine pancreas development | GO:0031018 | MF | 2 | 24 | 1.46×10^-4^ | 0.009 | BAK1\|IL6R |
| cyclin-dependent protein serine/threonine kinase activity | GO:0004693 | CC | 2 | 29 | 2.09×10^-4^ | 0.009 | CDK12\|CDK6 |
| cyclin-dependent protein kinase holoenzyme complex | GO:0000307 | MF | 2 | 30 | 2.23×10^-4^ | 0.009 | CDK12\|CDK6 |
| cyclin binding | GO:0030332 | BP | 2 | 30 | 2.23×10^-4^ | 0.009 | CDK12\|CDK6 |
| negative regulation of insulin receptor signaling pathway | GO:0046627 | MF | 2 | 32 | 2.52×10^-4^ | 0.010 | PRKCQ\|NCOA5 |
| single-stranded RNA binding | GO:0003727 | CC | 2 | 43 | 4.43×10^-4^ | 0.015 | C2\|ILF3 |
| plasma membrane | GO:0005886 | BP | 10 | 4619 | 6.44×10^-4^ | 0.021 | NAT2\|TSPAN14\|IL6R\|SLC12A5\|SH2B3\|PRKCQ\|SWAP70\|PLCL1\|BCAR1\|IKZF3 |
| cell chemotaxis | GO:0060326 | BP | 2 | 67 | 0.001 | 0.029 | PRKCQ\|BCAR1 |
| regulation of cell growth | GO:0001558 | CC | 2 | 68 | 0.001 | 0.029 | PRKCQ\|BCAR1 |
| cytosol | GO:0005829 | CC | 10 | 5095 | 0.001 | 0.033 | ILF3\|BAK1\|SH2B3\|CDK6\|PRKCQ\|C2\|PHETA1\|BCAR1\|IKZF3\|SPC24 |
| condensed chromosome kinetochore | GO:0000777 | BP | 2 | 86 | 0.002 | 0.033 | CFDP1\|SPC24 |
| cell division | GO:0051301 | BP | 3 | 346 | 0.002 | 0.033 | BCAR1\|CDK6\|SPC24 |
| intracellular signal transduction | GO:0035556 | CC | 3 | 369 | 0.002 | 0.033 | PRKCQ\|SH2B3\|PLCL1 |
| ruffle | GO:0001726 | CC | 2 | 98 | 0.002 | 0.033 | BCAR1\|CDK6 |
| nucleoplasm | GO:0005654 | BP | 8 | 3630 | 0.002 | 0.033 | NCOA5\|CDK12\|ILF3\|CDK6\|C2\|DCLRE1B\|IKZF3\|SPC24 |
| negative regulation of cell population proliferation | GO:0008285 | BP | 3 | 394 | 0.003 | 0.033 | SH2B3\|CDK6\|BAK1 |
| transmembrane receptor protein tyrosine kinase signaling pathway | GO:0007169 | BP | 2 | 123 | 0.003 | 0.033 | BCAR1\|SH2B3 |
| protein phosphorylation | GO:0006468 | BP | 3 | 451 | 0.004 | 0.033 | CDK12\|ILF3\|CDK6 |
| positive regulation of glomerular mesangial cell proliferation | GO:0072126 | BP | 1 | 5 | 0.004 | 0.033 | IL6R |
| neutrophil homeostasis | GO:0001780 | BP | 1 | 5 | 0.004 | 0.033 | SH2B3 |
| cellular ion homeostasis | GO:0006873 | BP | 1 | 5 | 0.004 | 0.033 | SLC12A5 |
| negative regulation of immunoglobulin production | GO:0002638 | MF | 1 | 5 | 0.004 | 0.033 | ZPBP2 |
| U1 snRNP binding | GO:1990446 | BP | 1 | 5 | 0.004 | 0.033 | C2 |
| thrombopoietin-mediated signaling pathway | GO:0038163 | BP | 1 | 5 | 0.004 | 0.033 | SH2B3 |
| cell dedifferentiation | GO:0043697 | BP | 1 | 5 | 0.004 | 0.033 | CDK6 |
| ciliary neurotrophic factor-mediated signaling pathway | GO:0070120 | BP | 1 | 5 | 0.004 | 0.033 | IL6R |
| apoptotic process involved in blood vessel morphogenesis | GO:1902262 | BP | 1 | 5 | 0.004 | 0.033 | BAK1 |
| thymocyte apoptotic process | GO:0070242 | CC | 1 | 5 | 0.004 | 0.033 | BAK1 |
| external side of apical plasma membrane | GO:0098591 | CC | 1 | 5 | 0.004 | 0.033 | NAT2 |
| commitment complex | GO:0000243 | BP | 1 | 5 | 0.004 | 0.033 | C2 |
| positive regulation of endoplasmic reticulum unfolded protein response | GO:1900103 | BP | 1 | 5 | 0.004 | 0.033 | BAK1 |
| cellular chloride ion homeostasis | GO:0030644 | BP | 1 | 5 | 0.004 | 0.033 | SLC12A5 |
| cellular response to interleukin-3 | GO:0036016 | BP | 1 | 6 | 0.005 | 0.033 | SH2B3 |
| negative regulation of response to cytokine stimulus | GO:0060761 | BP | 1 | 6 | 0.005 | 0.033 | SH2B3 |
| regulation of erythrocyte differentiation | GO:0045646 | BP | 1 | 6 | 0.005 | 0.033 | CDK6 |
| response to fungus | GO:0009620 | BP | 1 | 6 | 0.005 | 0.033 | BAK1 |
| positive regulation of mast cell chemotaxis | GO:0060754 | BP | 1 | 6 | 0.005 | 0.033 | SWAP70 |
| antigen receptor-mediated signaling pathway | GO:0050851 | BP | 1 | 6 | 0.005 | 0.033 | BCAR1 |
| regulation of platelet aggregation | GO:0090330 | BP | 1 | 6 | 0.005 | 0.033 | PRKCQ |
| gliogenesis | GO:0042063 | CC | 1 | 6 | 0.005 | 0.033 | CDK6 |
| Bcl-2 family protein complex | GO:0097136 | BP | 1 | 6 | 0.005 | 0.033 | BAK1 |
| mRNA 5'-splice site recognition | GO:0000395 | BP | 1 | 6 | 0.005 | 0.033 | C2 |
| positive regulation of T-helper 17 type immune response | GO:2000318 | MF | 1 | 6 | 0.005 | 0.033 | PRKCQ |
| interleukin-6 receptor binding | GO:0005138 | BP | 1 | 7 | 0.005 | 0.033 | IL6R |
| positive regulation of IRE1-mediated unfolded protein response | GO:1903896 | BP | 1 | 7 | 0.005 | 0.033 | BAK1 |
| negative regulation of chemokine-mediated signaling pathway | GO:0070100 | BP | 1 | 7 | 0.005 | 0.033 | SH2B3 |
| positive regulation of receptor binding | GO:1900122 | BP | 1 | 7 | 0.005 | 0.033 | PLCL1 |
| activation of cysteine-type endopeptidase activity involved in apoptotic process by cytochrome c | GO:0008635 | BP | 1 | 7 | 0.005 | 0.033 | BAK1 |
| regulation of peptidyl-serine phosphorylation | GO:0033135 | MF | 1 | 7 | 0.005 | 0.033 | PLCL1 |
| N6-methyladenosine-containing RNA binding | GO:1990247 | BP | 1 | 7 | 0.005 | 0.033 | C2 |
| negative regulation of fibroblast apoptotic process | GO:2000270 | BP | 1 | 7 | 0.005 | 0.033 | CFDP1 |
| negative regulation of monocyte differentiation | GO:0045656 | BP | 1 | 7 | 0.005 | 0.033 | CDK6 |
| protection from non-homologous end joining at telomere | GO:0031848 | BP | 1 | 7 | 0.005 | 0.033 | DCLRE1B |
| vascular endothelial growth factor production | GO:0010573 | BP | 1 | 7 | 0.005 | 0.033 | IL6R |
| endothelin receptor signaling pathway | GO:0086100 | BP | 1 | 7 | 0.005 | 0.033 | BCAR1 |
| positive regulation of activation of Janus kinase activity | GO:0010536 | BP | 1 | 7 | 0.005 | 0.033 | IL6R |
| generation of neurons | GO:0048699 | BP | 1 | 7 | 0.005 | 0.033 | CDK6 |
| RNA splicing | GO:0008380 | BP | 2 | 160 | 0.006 | 0.033 | CDK12\|C2 |
| negative regulation of T cell apoptotic process | GO:0070233 | BP | 1 | 8 | 0.006 | 0.033 | PRKCQ |
| B cell apoptotic process | GO:0001783 | BP | 1 | 8 | 0.006 | 0.033 | BAK1 |
| negative regulation of endoplasmic reticulum calcium ion concentration | GO:0032471 | MF | 1 | 8 | 0.006 | 0.033 | BAK1 |
| U1 snRNA binding | GO:0030619 | MF | 1 | 8 | 0.006 | 0.033 | C2 |
| L-glutamine transmembrane transporter activity | GO:0015186 | BP | 1 | 8 | 0.006 | 0.033 | NAT2 |
| glutamine transport | GO:0006868 | CC | 1 | 8 | 0.006 | 0.033 | NAT2 |
| zona pellucida receptor complex | GO:0002199 | BP | 1 | 8 | 0.006 | 0.033 | ZPBP2 |
| telomere maintenance via telomere lengthening | GO:0010833 | CC | 1 | 8 | 0.006 | 0.033 | DCLRE1B |
| cyclin/CDK positive transcription elongation factor complex | GO:0008024 | BP | 1 | 8 | 0.006 | 0.033 | CDK12 |
| T cell receptor signaling pathway | GO:0050852 | BP | 2 | 174 | 0.007 | 0.034 | PRKCQ\|BCAR1 |
| phosphorylation of RNA polymerase II C-terminal domain | GO:0070816 | BP | 1 | 9 | 0.007 | 0.034 | CDK12 |
| myeloid cell homeostasis | GO:0002262 | MF | 1 | 9 | 0.007 | 0.034 | BAK1 |
| potassium:chloride symporter activity | GO:0015379 | BP | 1 | 9 | 0.007 | 0.034 | SLC12A5 |
| actin filament reorganization | GO:0090527 | CC | 1 | 9 | 0.007 | 0.034 | BCAR1 |
| lamellipodium | GO:0030027 | BP | 2 | 185 | 0.007 | 0.034 | SWAP70\|BCAR1 |
| positive regulation of mitochondrial outer membrane permeabilization involved in apoptotic signaling pathway | GO:1901030 | MF | 1 | 10 | 0.008 | 0.034 | BAK1 |
| phospholipase C activity | GO:0004629 | BP | 1 | 10 | 0.008 | 0.034 | PLCL1 |
| regulation of B cell differentiation | GO:0045577 | BP | 1 | 10 | 0.008 | 0.034 | IKZF3 |
| response to UV-C | GO:0010225 | BP | 1 | 10 | 0.008 | 0.034 | BAK1 |
| regulation of mitochondrial membrane permeability | GO:0046902 | CC | 1 | 10 | 0.008 | 0.034 | BAK1 |
| tetraspanin-enriched microdomain | GO:0097197 | BP | 1 | 10 | 0.008 | 0.034 | TSPAN14 |
| vagina development | GO:0060068 | BP | 1 | 10 | 0.008 | 0.034 | BAK1 |
| biological_process | GO:0008150 | BP | 3 | 594 | 0.008 | 0.034 | MKRN2\|CFDP1\|ILF3 |
| receptor recycling | GO:0001881 | MF | 1 | 11 | 0.008 | 0.034 | PHETA1 |
| neutral amino acid transmembrane transporter activity | GO:0015175 | BP | 1 | 11 | 0.008 | 0.034 | NAT2 |
| chloride ion homeostasis | GO:0055064 | BP | 1 | 11 | 0.008 | 0.034 | SLC12A5 |
| regulation of apoptotic process | GO:0042981 | BP | 2 | 197 | 0.008 | 0.034 | BCAR1\|IKZF3 |
| lateral ventricle development | GO:0021670 | BP | 1 | 12 | 0.009 | 0.034 | CDK6 |
| negative regulation of tyrosine phosphorylation of STAT protein | GO:0042532 | BP | 1 | 12 | 0.009 | 0.034 | SH2B3 |
| positive regulation of calcium ion transport into cytosol | GO:0010524 | MF | 1 | 12 | 0.009 | 0.034 | BAK1 |
| 5'-3' exonuclease activity | GO:0008409 | BP | 1 | 12 | 0.009 | 0.034 | DCLRE1B |
| regulation of MAP kinase activity | GO:0043405 | BP | 1 | 12 | 0.009 | 0.034 | CDK12 |
| type B pancreatic cell development | GO:0003323 | BP | 1 | 12 | 0.009 | 0.034 | CDK6 |
| hepatocyte growth factor receptor signaling pathway | GO:0048012 | BP | 1 | 12 | 0.009 | 0.034 | BCAR1 |
| protein localization to synapse | GO:0035418 | BP | 1 | 12 | 0.009 | 0.034 | ILF3 |
| negative regulation of platelet aggregation | GO:0090331 | BP | 1 | 12 | 0.009 | 0.034 | SH2B3 |
| neurotransmitter uptake | GO:0001504 | MF | 1 | 12 | 0.009 | 0.034 | NAT2 |
| transmembrane receptor protein tyrosine kinase adaptor activity | GO:0005068 | BP | 1 | 13 | 0.010 | 0.034 | SH2B3 |
| neutral amino acid transport | GO:0015804 | BP | 1 | 13 | 0.010 | 0.034 | NAT2 |
| negative regulation of receptor signaling pathway via JAK-STAT | GO:0046426 | BP | 1 | 13 | 0.010 | 0.034 | SH2B3 |
| positive regulation of actin filament bundle assembly | GO:0032233 | CC | 1 | 13 | 0.010 | 0.034 | SWAP70 |
| pore complex | GO:0046930 | MF | 1 | 13 | 0.010 | 0.034 | BAK1 |
| inositol 1,4,5 trisphosphate binding | GO:0070679 | BP | 1 | 13 | 0.010 | 0.034 | PLCL1 |
| positive regulation of transcription elongation from RNA polymerase II promoter | GO:0032968 | BP | 1 | 13 | 0.010 | 0.034 | CDK12 |
| positive regulation of leukocyte chemotaxis | GO:0002690 | MF | 1 | 13 | 0.010 | 0.034 | IL6R |
| protein kinase activity | GO:0004672 | CC | 2 | 216 | 0.010 | 0.034 | PRKCQ\|CDK12 |
| basolateral plasma membrane | GO:0016323 | BP | 2 | 216 | 0.010 | 0.034 | NAT2\|IL6R |
| establishment or maintenance of transmembrane electrochemical gradient | GO:0010248 | BP | 1 | 14 | 0.010 | 0.035 | BAK1 |
| limb morphogenesis | GO:0035108 | BP | 1 | 14 | 0.010 | 0.035 | BAK1 |
| L-alpha-amino acid transmembrane transport | GO:1902475 | BP | 1 | 14 | 0.010 | 0.035 | NAT2 |
| activation of cysteine-type endopeptidase activity | GO:0097202 | BP | 1 | 15 | 0.011 | 0.035 | BAK1 |
| dendritic spine development | GO:0060996 | BP | 1 | 15 | 0.011 | 0.035 | SLC12A5 |
| telomere capping | GO:0016233 | BP | 1 | 15 | 0.011 | 0.035 | DCLRE1B |
| regulation of synaptic transmission, GABAergic | GO:0032228 | BP | 1 | 15 | 0.011 | 0.035 | PLCL1 |
| negative regulation of stem cell differentiation | GO:2000737 | BP | 1 | 15 | 0.011 | 0.035 | CDK12 |
| cellular response to chemokine | GO:1990869 | BP | 1 | 16 | 0.012 | 0.035 | SH2B3 |
| astrocyte development | GO:0014002 | MF | 1 | 16 | 0.012 | 0.035 | CDK6 |
| calcium-dependent protein kinase C activity | GO:0004698 | MF | 1 | 16 | 0.012 | 0.035 | PRKCQ |
| L-amino acid transmembrane transporter activity | GO:0015179 | BP | 1 | 16 | 0.012 | 0.035 | NAT2 |
| dentate gyrus development | GO:0021542 | BP | 1 | 16 | 0.012 | 0.035 | CDK6 |
| positive regulation of T cell activation | GO:0050870 | BP | 1 | 16 | 0.012 | 0.035 | PRKCQ |
| potassium ion homeostasis | GO:0055075 | BP | 1 | 16 | 0.012 | 0.035 | SLC12A5 |
| hematopoietic stem cell differentiation | GO:0060218 | BP | 1 | 16 | 0.012 | 0.035 | CDK6 |
| cellular response to hepatocyte growth factor stimulus | GO:0035729 | CC | 1 | 16 | 0.012 | 0.035 | BCAR1 |
| U2-type prespliceosome | GO:0071004 | BP | 1 | 17 | 0.012 | 0.035 | C2 |
| mitochondrial fusion | GO:0008053 | MF | 1 | 17 | 0.012 | 0.035 | BAK1 |
| chloride transmembrane transporter activity | GO:0015108 | BP | 1 | 17 | 0.012 | 0.035 | SLC12A5 |
| endoplasmic reticulum calcium ion homeostasis | GO:0032469 | BP | 1 | 17 | 0.012 | 0.035 | BAK1 |
| positive regulation of telomere capping | GO:1904355 | BP | 1 | 17 | 0.012 | 0.035 | PRKCQ |
| isotype switching | GO:0045190 | BP | 1 | 17 | 0.012 | 0.035 | SWAP70 |
| megakaryocyte development | GO:0035855 | BP | 1 | 17 | 0.012 | 0.035 | SH2B3 |
| interleukin-6-mediated signaling pathway | GO:0070102 | BP | 1 | 18 | 0.013 | 0.035 | IL6R |
| positive regulation of interleukin-17 production | GO:0032740 | BP | 1 | 18 | 0.013 | 0.035 | PRKCQ |
| acrosome assembly | GO:0001675 | MF | 1 | 18 | 0.013 | 0.035 | ZPBP2 |
| GABA receptor binding | GO:0050811 | BP | 1 | 18 | 0.013 | 0.035 | PLCL1 |
| embryonic hemopoiesis | GO:0035162 | BP | 1 | 18 | 0.013 | 0.035 | SH2B3 |
| negative regulation of myeloid cell differentiation | GO:0045638 | MF | 1 | 18 | 0.013 | 0.035 | CDK6 |
| RNA binding | GO:0003723 | CC | 4 | 1366 | 0.014 | 0.035 | C2\|MKRN2\|ILF3\|NCOA5 |
| U1 snRNP | GO:0005685 | BP | 1 | 19 | 0.014 | 0.035 | C2 |
| cell volume homeostasis | GO:0006884 | BP | 1 | 19 | 0.014 | 0.035 | SLC12A5 |
| erythrocyte development | GO:0048821 | BP | 1 | 19 | 0.014 | 0.035 | SH2B3 |
| positive regulation of chemokine production | GO:0032722 | BP | 1 | 19 | 0.014 | 0.035 | IL6R |
| negative regulation of cellular senescence | GO:2000773 | BP | 1 | 19 | 0.014 | 0.035 | CDK6 |
| protein maturation | GO:0051604 | BP | 1 | 19 | 0.014 | 0.035 | TSPAN14 |
| 3'-UTR-mediated mRNA stabilization | GO:0070935 | BP | 1 | 19 | 0.014 | 0.035 | C2 |
| negative regulation of telomere maintenance via telomerase | GO:0032211 | BP | 1 | 20 | 0.014 | 0.036 | C2 |
| positive regulation of proteolysis | GO:0045862 | CC | 1 | 20 | 0.014 | 0.036 | BAK1 |
| telomerase holoenzyme complex | GO:0005697 | BP | 1 | 20 | 0.014 | 0.036 | C2 |
| neurotrophin TRK receptor signaling pathway | GO:0048011 | BP | 1 | 20 | 0.014 | 0.036 | BCAR1 |
| B cell homeostasis | GO:0001782 | MF | 1 | 21 | 0.015 | 0.038 | BAK1 |
| telomerase RNA binding | GO:0070034 | BP | 1 | 22 | 0.016 | 0.039 | C2 |
| release of cytochrome c from mitochondria | GO:0001836 | BP | 1 | 22 | 0.016 | 0.039 | BAK1 |
| response to drug | GO:0042493 | BP | 2 | 280 | 0.016 | 0.039 | SLC12A5\|BAK1 |
| membrane protein ectodomain proteolysis | GO:0006509 | CC | 1 | 23 | 0.016 | 0.040 | PRKCQ |
| integral component of mitochondrial outer membrane | GO:0031307 | MF | 1 | 23 | 0.016 | 0.040 | BAK1 |
| identical protein binding | GO:0042802 | BP | 4 | 1456 | 0.017 | 0.041 | C2\|ZBTB9\|IKZF3\|BAK1 |
| positive regulation of interleukin-4 production | GO:0032753 | MF | 1 | 24 | 0.017 | 0.041 | PRKCQ |
| ATP binding | GO:0005524 | BP | 4 | 1463 | 0.017 | 0.041 | PRKCQ\|CDK12\|SWAP70\|CDK6 |
| negative regulation of peptidyl-serine phosphorylation | GO:0033137 | BP | 1 | 25 | 0.018 | 0.042 | BAK1 |
| sphingolipid metabolic process | GO:0006665 | MF | 1 | 25 | 0.018 | 0.042 | ZPBP2 |
| mRNA 3'-UTR AU-rich region binding | GO:0035925 | MF | 1 | 26 | 0.018 | 0.042 | ILF3 |
| phosphatidylinositol phospholipase C activity | GO:0004435 | MF | 1 | 26 | 0.018 | 0.042 | PLCL1 |
| poly(U) RNA binding | GO:0008266 | BP | 1 | 26 | 0.018 | 0.042 | C2 |
| cellular response to unfolded protein | GO:0034620 | MF | 1 | 26 | 0.018 | 0.042 | BAK1 |
| metal ion binding | GO:0046872 | BP | 5 | 2298 | 0.019 | 0.043 | PRKCQ\|ZBTB9\|MKRN2\|IKZF3\|BAK1 |
| response to gamma radiation | GO:0010332 | BP | 1 | 27 | 0.019 | 0.043 | BAK1 |
| positive regulation of cell-matrix adhesion | GO:0001954 | BP | 1 | 27 | 0.019 | 0.043 | CDK6 |
| gamma-aminobutyric acid signaling pathway | GO:0007214 | BP | 1 | 27 | 0.019 | 0.043 | PLCL1 |
| positive regulation of release of cytochrome c from mitochondria | GO:0090200 | BP | 1 | 28 | 0.020 | 0.043 | BAK1 |
| spliceosomal snRNP assembly | GO:0000387 | BP | 1 | 28 | 0.020 | 0.043 | C2 |
| regulation of mitochondrial membrane potential | GO:0051881 | BP | 1 | 28 | 0.020 | 0.043 | BAK1 |
| regulation of cell motility | GO:2000145 | MF | 1 | 29 | 0.020 | 0.044 | CDK6 |
| signaling receptor complex adaptor activity | GO:0030159 | CC | 1 | 29 | 0.020 | 0.044 | SH2B3 |
| nucleus | GO:0005634 | BP | 8 | 5208 | 0.021 | 0.044 | CDK12\|ZBTB9\|ILF3\|CDK6\|C2\|SWAP70\|IKZF3\|ZPBP2 |
| mesoderm development | GO:0007498 | BP | 1 | 30 | 0.021 | 0.045 | IKZF3 |
| platelet-derived growth factor receptor signaling pathway | GO:0048008 | MF | 1 | 30 | 0.021 | 0.045 | BCAR1 |
| protein tyrosine kinase binding | GO:1990782 | BP | 1 | 30 | 0.021 | 0.045 | SH2B3 |
| mitotic cell cycle checkpoint | GO:0007093 | BP | 1 | 31 | 0.022 | 0.046 | DCLRE1B |
| T cell differentiation in thymus | GO:0033077 | BP | 1 | 31 | 0.022 | 0.046 | CDK6 |
| blood vessel remodeling | GO:0001974 | BP | 1 | 32 | 0.022 | 0.047 | BAK1 |
| B cell activation | GO:0042113 | BP | 1 | 32 | 0.022 | 0.047 | IKZF3 |
| extrinsic apoptotic signaling pathway in absence of ligand | GO:0097192 | BP | 1 | 33 | 0.023 | 0.048 | BAK1 |
| positive regulation of telomere maintenance via telomerase | GO:0032212 | BP | 1 | 34 | 0.024 | 0.048 | PRKCQ |
| amino acid transmembrane transport | GO:0003333 | BP | 1 | 34 | 0.024 | 0.048 | NAT2 |
| intrinsic apoptotic signaling pathway in response to endoplasmic reticulum stress | GO:0070059 | BP | 1 | 34 | 0.024 | 0.048 | BAK1 |
| ATP-dependent chromatin remodeling | GO:0043044 | BP | 1 | 34 | 0.024 | 0.048 | C2 |
| positive regulation of telomerase activity | GO:0051973 | MF | 1 | 34 | 0.024 | 0.048 | PRKCQ |
| protein-containing complex binding | GO:0044877 | BP | 2 | 349 | 0.024 | 0.048 | DCLRE1B\|BAK1 |
| actin filament bundle assembly | GO:0051017 | MF | 1 | 35 | 0.024 | 0.049 | SWAP70 |
| enzyme binding | GO:0019899 | BP | 2 | 353 | 0.025 | 0.049 | TSPAN14\|IL6R |
| acute-phase response | GO:0006953 | BP | 1 | 36 | 0.025 | 0.049 | IL6R |

Abbreviations: CC, cellular component; BP, biological process; MF, molecular function.

**Table S14.** The enriched KEGG pathway terms of the mapped genes.

| Term | ID | Input number | Background number | *P* | FDR | Input |
| --- | --- | --- | --- | --- | --- | --- |
| Human cytomegalovirus infection | hsa05163 | 4 | 225 | 1.78×10^-5^ | 0.002 | BAK1\|BCAR1\|CDK6\|IL6R |
| GABAergic synapse | hsa04727 | 3 | 89 | 3.49×10^-5^ | 0.003 | SLC12A5\|NAT2\|PLCL1 |
| Non-small cell lung cancer | hsa05223 | 2 | 66 | 0.001 | 0.028 | CDK6\|BAK1 |
| Melanoma | hsa05218 | 2 | 72 | 0.001 | 0.028 | CDK6\|BAK1 |
| Glioma | hsa05214 | 2 | 75 | 0.001 | 0.028 | CDK6\|BAK1 |
| Pancreatic cancer | hsa05212 | 2 | 75 | 0.001 | 0.028 | CDK6\|BAK1 |
| Chronic myeloid leukemia | hsa05220 | 2 | 76 | 0.001 | 0.028 | CDK6\|BAK1 |
| Small cell lung cancer | hsa05222 | 2 | 93 | 0.002 | 0.032 | CDK6\|BAK1 |
| Th17 cell differentiation | hsa04659 | 2 | 107 | 0.003 | 0.032 | PRKCQ\|IL6R |
| Measles | hsa05162 | 2 | 138 | 0.004 | 0.032 | CDK6\|BAK1 |
| Breast cancer | hsa05224 | 2 | 147 | 0.005 | 0.032 | CDK6\|BAK1 |
| Hepatitis C | hsa05160 | 2 | 155 | 0.005 | 0.032 | CDK6\|BAK1 |
| Pathways in cancer | hsa05200 | 3 | 530 | 0.006 | 0.032 | BAK1\|CDK6\|IL6R |
| Influenza A | hsa05164 | 2 | 167 | 0.006 | 0.032 | CDK6\|BAK1 |
| Hepatocellular carcinoma | hsa05225 | 2 | 168 | 0.006 | 0.032 | CDK6\|BAK1 |
| Kaposi sarcoma-associated herpesvirus infection | hsa05167 | 2 | 186 | 0.007 | 0.033 | CDK6\|BAK1 |
| Viral carcinogenesis | hsa05203 | 2 | 201 | 0.009 | 0.034 | CDK6\|BAK1 |
| Epstein-Barr virus infection | hsa05169 | 2 | 201 | 0.009 | 0.034 | CDK6\|BAK1 |
| MicroRNAs in cancer | hsa05206 | 2 | 299 | 0.018 | 0.044 | CDK6\|BAK1 |
| Human papillomavirus infection | hsa05165 | 2 | 330 | 0.022 | 0.048 | CDK6\|BAK1 |
| Apoptosis - multiple species | hsa04215 | 1 | 33 | 0.023 | 0.050 | BAK1 |

**Table S15.** The effects of different combinations of rheumatoid arthritis/cardiovascular diseases status and overlapped modifiable risk factors (diastolic blood pressure)

|  |  |  |  | Model 1^*^ | | |  | Model 2^#^ | | |
| --- | --- | --- | --- | --- | --- | --- | --- | --- | --- | --- |
|  | Individuals | Events |  | HR | 95% CI | *P* |  | HR | 95% CI | *P* |
| **RA-CVD**^¶^ |  |  |  |  |  |  |  |  |  |  |
| RA (-) DBP (-) | 273391 | 31562 |  | Reference |  |  |  | Reference |  |  |
| RA (+) DBP (-) | 84192 | 12955 |  | 1.16 | 1.14-1.19 | <2.20×10^-16^ |  | 1.05 | 1.02-1.07 | 4.43×10^-5^ |
| RA (-) DBP (+) | 5415 | 1005 |  | 1.35 | 1.27-1.44 | <2.20×10^-16^ |  | 1.20 | 1.13-1.28 | 3.24×10^-8^ |
| RA (+) DBP (+) | 1665 | 351 |  | 1.49 | 1.34-1.65 | 1.47×10^-13^ |  | 1.22 | 1.09-1.36 | 4.80×10^-4^ |
| *P_trend_* |  |  |  | <2.20×10^-16^ |  |  |  | 9.09×10^-12^ |  |  |
|  |  |  |  |  |  |  |  |  |  |  |
| **CVD-RA**^§^ |  |  |  |  |  |  |  |  |  |  |
| CVD (-) DBP (-) | 273452 | 2676 |  | Reference |  |  |  | Reference |  |  |
| CVD (+) DBP (-) | 53357 | 731 |  | 1.19 | 1.10-1.30 | 4.87×10^-5^ |  | 1.04 | 0.96-1.14 | 0.343 |
| CVD (-) DBP (+) | 84210 | 833 |  | 1.05 | 0.97-1.13 | 0.267 |  | 0.96 | 0.89-1.04 | 0.358 |
| CVD (+) DBP (+) | 18358 | 219 |  | 1.13 | 0.98-1.30 | 0.095 |  | 0.96 | 0.84-1.11 | 0.603 |
| *P_trend_* |  |  |  | 0.029 |  |  |  | 0.401 |  |  |

*Model 1: adjusted for sex and age.

^#^Model 2: adjusted for sex, age, body mass index, smoking status, alcohol drinking frequency, education levels, physical activity, type 2 diabetes and total cholesterol when evaluated the combined effects of rheumatoid arthritis and diabolic blood pressure in the risk of cardiovascular disease; adjusted for sex, age, body mass index, smoking status, alcohol drinking frequency, education levels and physical activity when evaluated the combined effects of cardiovascular diseases and diabolic blood pressure in the risk of rheumatoid arthritis.

^¶^ Participants were grouped on the basis of their diabolic blood pressure levels (normal [< 90 mmHg] vs. high [≥ 90 mmHg]) and rheumatoid arthritis (no vs. yes) ：RA(-)DBP (-), RA(+) DBP(-), RA(-) DBP(+) and RA(+) DBP(+).

^§^ Participants were grouped on the basis of their diabolic blood pressure levels (normal [< 90 mmHg] vs. high [≥ 90 mmHg]) and cardiovascular disease (no vs. yes) ：CVD(-)DBP (-), CVD(+) DBP(-), CVD(-) DBP(+) and CVD(+) DBP(+).

Abbreviations: CVD, cardiovascular diseases; DBP, diabolic blood pressure; HR, hazard ratio; RA, rheumatoid arthritis.
